# Do Combined Resistance and Aerobic Exercise Programs Cause an Interference Effect in Women with Breast Cancer? A Systematic Review and Network Meta-analysis

**DOI:** 10.1007/s40279-026-02402-x

**Published:** 2026-02-20

**Authors:** Pedro Lopez, Anderson Rech, Maria Petropoulou, Caroline B. Silveira, Talita Molinari, Cindranne Torres Muller, Priscila Casara, Francesco Bettariga, Favil Singh, Régis Radaelli

**Affiliations:** 1 Grupo de Pesquisa Em Exercício Para Populações Clínicas (GPCLIN), Universidade de Caxias do Sul, Caxias do Sul, Rio Grande do Sul Brazil; 2 Programa de Pós-Graduação em Ciências da Saúde, Universidade de Caxias do Sul, Caxias do Sul, Rio Grande do Sul Brazil; 3 Pleural Medicine Unit, Institute for Respiratory Health, Perth, Western Australia Australia; 4 School of Medical and Health Sciences, Edith Cowan University, Joondalup, Western Australia Australia; 5 Curso de Educação Física, Universidade de Caxias do Sul, Caxias do Sul, Rio Grande do Sul Brazil; 6 Institute of Medical Biometry and Statistics, Faculty of Medicine and Medical Center, University of Freiburg, Freiburg, Germany; 7 Laboratório de Pesquisa do Exercício (LAPEX), Escola de Educação Física, Fisioterapia e Dança, Universidade Federal do Rio Grande do Sul, Porto Alegre, Rio Grande do Sul Brazil; 8 Curso de Medicina, Universidade de Caxias do Sul, Caxias do Sul, Rio Grande do Sul Brazil; 9 Exercise Medicine Research Institute, Edith Cowan University, Joondalup, Western Australia Australia; 10 Egas Moniz Center for Interdisciplinary Research (CiiEM), Egas Moniz School of Health and Science, Caparica, Almada Portugal

## Abstract

**Background:**

Exercise medicine has gained significant recognition owing to its demonstrated benefits throughout the breast cancer treatment continuum. While resistance exercise (RE) promotes improvements in lean mass and muscle strength, aerobic exercise (AE) enhances cardiorespiratory fitness, with several studies investigating both exercise modes in patients with breast cancer. However, because of an effect often referred to as interference effect, it is hypothesised that combining resistance and aerobic exercise (COMB) may compromise gains in lean mass and muscle strength and other outcomes in cancer populations.

**Objective:**

This study aims to investigate the presence of an interference effect from prescribing COMB compared with RE on fatigue, lean mass, physical function and muscle strength in women with breast cancer. In addition, it examines a range of demographic, clinical and exercise prescription moderators within this population.

**Methods:**

We searched seven databases from inception to January 2024 (PROSPERO CRD42023491118), with an updated search in April 2025. Eligible trials examined the effects of RE, AE and/or COMB in women diagnosed with breast cancer. The primary outcomes for this review were cancer-related fatigue, lean mass, physical function and/or lower-limb muscle strength. A random-effects network meta-analysis was undertaken to examine the effect of different exercise programs and controls, with specific focus on the comparisons between RE and COMB. Differences between RE and COMB above 0.20 standardised mean difference (SMD) were an indicative of a potential interference effect.

**Results:**

We included a total of 131 articles describing 116 randomised trials (*n* = 9206). Both RE (SMD − 0.52, 95% CI − 0.83 to − 0.21, *p* = 0.001) and COMB (SMD − 0.47, 95% CI − 0.65 to − 0.29, *p* < 0.001) similarly reduced fatigue compared with controls. However, a potential interference effect was observed on fatigue during surgery (SMD − 0.23, 95% CI − 0.58 to 0.12, *p* = 0.191) and chemotherapy (SMD − 0.22, 95% CI − 0.59 to 0.16, *p* = 0.257), with RE showing greater benefits than COMB. For physical function, both RE (SMD 0.86, 95% CI 0.41–1.30, *p* < 0.001) and COMB (SMD 0.90, 95% CI 0.58–1.22, *p* < 0.001) improved outcomes compared with controls, though RE was superior to COMB in patients receiving hormone therapy (SMD 0.62, 95% CI − 0.56 to 1.81, *p* = 0.303). Differences between RE and COMB in lean mass (MD 0.18 kg, 95% CI − 0.17–0.53 kg, *p* = 0.303) and muscle strength (SMD 0.43, 95% CI − 0.01–0.86, *p* = 0.056) were not significant in the main analyses; however, sensitivity analyses omitting the outliers indicated significant differences favouring RE for lean mass (MD 0.36 kg, 95% CI 0.15–0.57 kg, *p* < 0.001) and muscle strength (SMD 0.40, 95% CI 0.08–0.71, *p* = 0.014).

**Conclusions:**

Our findings emphasise the importance of personalised exercise medicine targeted to treatment characteristics in women with breast cancer. While COMB may offer benefits across the outcomes investigated, prescribing RE alone may be preferable to minimize potential interference effects on lean mass and muscle strength, regardless of demographic, clinical and exercise prescription characteristics, as well as fatigue and physical function tests, particularly during adjuvant and hormonal therapies.

**Supplementary Information:**

The online version contains supplementary material available at 10.1007/s40279-026-02402-x.

## Key Points

**Table:** 

It has been hypothesised that combining resistance and aerobic exercise may compromise fatigue, lean mass, physical function and muscle strength improvements in women with breast cancer.
Combined resistance and aerobic exercise can result in a potential interference effect on fatigue and physical function compared with resistance exercise alone when prescribed during adjuvant and hormone therapy, respectively.
Combined resistance and aerobic exercise can result in a potential interference effect on lean mass and muscle strength compared with resistance exercise alone, irrespective of demographic, clinical and exercise prescription characteristics.
Resistance exercise alone may be preferred to better target lean mass and muscle strength and minimize the potential interference effect in fatigue and physical function compared with combined resistance and aerobic exercise.

## Introduction

Female breast cancer is the most diagnosed cancer worldwide with 2.3 million new cases and 660,000 deaths worldwide in 2022 [1]. Treatments for stages I–III usually include breast surgery (mastectomy or breast-conserving), radiotherapy, adjuvant or neoadjuvant chemotherapy and hormone therapy, with targeted- and immunotherapy recommended for specific cases. The choice of treatment depends on many factors such as type, stage, tumour characteristics, lymph node involvement and presence of metastasis. While breast cancer treatment regimens improve survival rates for patients with localised and regional disease, patients often experience a range of neural, musculoskeletal and metabolic side effects, including severe fatigue [2–4], loss of lean/muscle mass [5], impaired physical function [6] and reduced muscle strength [7]. These side effects contribute to a high symptom burden, diminished quality of life and an increased risk of mortality and cancer recurrence [2–7].

Exercise medicine has indeed gained significant recognition for its role in cancer management, endorsed by numerous professional organisations [8–13] owing to its demonstrated benefits on physical fitness, body composition, quality of life and psychological well-being throughout the cancer treatment continuum. It has also been hypothesised that exercise is associated with reduced mortality risk [14, 15]. In recent years, studies have specifically targeted symptoms and health needs to refine exercise programs and enhance the precision of exercise medicine for women with breast cancer [16–20]. These benefits are achieved through the application of key exercise training principles such as *specificity*, *progression*, *overload*, *initial values*, *reversibility* and *diminishing returns* [21]. Regarding *specificity*, it is well known that resistance exercise (RE; i.e., anabolic exercise; performing sets of repeated movements against an external load) promotes improvements in lean mass and muscle strength, while aerobic exercise (AE; i.e., activity involving large muscle groups and performed in a continuous or intermittent fashion over an extended period of time, such as cycling, swimming, jogging or running) enhances cardiorespiratory fitness, with several studies investigating both exercise modes in patients with breast cancer [16, 22, 23]. Combining RE and AE (i.e., combined exercise or concurrent training) within a periodised exercise regimen has been considered an effective approach to achieve simultaneous benefits in neuromuscular, cardiorespiratory, psychological well-being and quality of life in the oncology setting [8–13].

Interestingly, multiple studies suggest that combining RE and AE (COMB) may compromise gains in lean mass and muscle strength compared with RE alone in healthy adults [24–26], while it may affect cardiorespiratory gains depending on training status [26]. This consequence, often referred to as the *interference effect* or *concurrent training effect*, has been attributed to various mechanisms [27–29], including the molecular profiles generated by RE and AE, and acute and chronic residual fatigue. Given the complex interactions between treatment regimens, cancer-related fatigue and exercise stimuli, it may well be that women with breast cancer may experience diminished benefits from COMB compared with RE alone on fatigue, lean mass, physical function and muscle strength. This has been also hypothesised in other cancer populations [30, 31]. On the other hand, differences in cardiorespiratory fitness were previously considered minimal between COMB and AE alone in patients with cancer [32]. Although previous exercise guidelines [11, 12] recommend tailoring AE or RE on the basis of patient needs and goals, no studies have specifically examined the potential interference effect of prescribing COMB in women with breast cancer. As a result, a comprehensive assessment of the literature is needed to clarify this effect and provide more targeted exercise recommendations for this population.

Numerous demographic and clinical (e.g., age, body mass index [BMI], cancer stage, metastasis, treatment status, treatment regimen) and exercise prescription factors (e.g., duration, delivery, volume, intensity) can influence the interference effect of prescribing COMB in women with breast cancer. Exercise medicine plays varying roles throughout the cancer care continuum, including prehabilitation, intrahabilitation and rehabilitation, while interacting with different treatment modalities (e.g., surgery, radiation, chemotherapy, hormone therapy) and scheduling and sequence (e.g., treatment modality, dose, frequency, length) [33]. This complex landscape also necessitates a comprehensive assessment of exercise mode, frequency, dosage and intensity. Therefore, we aimed to compare the effects of COMB, RE alone, AE alone on fatigue, lean mass, physical function and muscle strength to investigate the presence of an interference effect from prescribing COMB in women diagnosed with breast cancer. In addition, we examined a range of demographic, clinical and exercise prescription moderators in this group. Given the similar adaptations between COMB and AE and minimal interference effect on cardiorespiratory fitness [32], the focus of this systematic review and network meta-analysis was on the comparisons between RE and COMB on fatigue, lean mass, physical function and muscle strength.

## Methods

All procedures undertaken in the present study were reported according to the Cochrane Back Review Group (CRBG) [34], the Implementing Prisma in Exercise, Rehabilitation, Sport medicine and SporTs science (PERSiST) [35] and the Preferred Reporting Items for Systematic Reviews and Meta-Analyses for Network Meta-analyses (PRISMA-NMA) [36] statement. This systematic review and network meta-analysis was prospectively registered on the International Register of Systematic Review Protocols (PROSPERO identifier: CRD42023491118).

### Study Eligibility Criteria

Studies were included when meeting the following criteria regarding participants, intervention, comparator, outcomes and study design (PICOS): i) studies involving and reporting outcomes for women aged ≥ 18 years old with primary breast cancer of any type, stage and grade; ii) studies undertaking AE, RE or COMB programs, delivered as supervised, unsupervised or hybrid programs following breast cancer diagnosis; iii) studies comparing AE, RE or COMB programs with or without non-exposed control groups, usual care, no formal intervention, waitlist or minimal exposure control group (i.e., walking group); iv) studies measuring cancer-related fatigue or vitality, lean mass, muscle hypertrophy, physical function (i.e., 30-s chair rise, chair rise test, 400-m walk test, 6-min walk test and/or or timed-up and go) and/or lower-limb muscle strength (i.e., leg press and/or knee extension one-repetition maximum, or isokinetic knee extension test (isometric and dynamic at 60° s^−1^ and 120° s^−1^)); and v) randomised controlled trials.

The exclusion criteria were: (i) women with breast cancer randomised to exercise interventions lasting < 4 weeks; (ii) women with breast cancer randomised to exercise interventions combined with any nutritional approach (e.g., healthy diet, caloric restriction, protein supplementation); and iii) studies written in a language other than English, Portuguese or Spanish.

### Search Strategy and Study Selection Process

A systematic search was conducted by a researcher (PL) using CINAHL, Embase, LILACS, PubMed, Scielo, SPORTDiscus and Web of Science databases from inception to 10 January 2024. The search strategy was undertaken using controlled vocabulary and free-text terms as presented in the Electronic Supplementary Material (ESM) Appendix S1. A manual search of references in previous systematic reviews was performed to detect potentially eligible articles for inclusion. In addition, an updated search was performed in PubMed in April 2025. During the screening phase, titles and abstracts were independently assessed according to the eligibility criteria. Eligibility was independently assessed in triplicate (P.L., C.B.S. and T.M.) with differences resolved by consensus when disagreements occurred. Abstracts that did not provide sufficient information were selected for full-text evaluation. Full-text articles that met the criteria were retrieved and read independently by the researchers (P.L., C.B.S. and T.M.) and assessed for study inclusion.

### Data Extraction and Endpoints

Two researchers (P.L., C.B.S.) extracted data using a standardised form. Relevant information extracted from studies included publication information (i.e., authors, year of publication), demographic and clinical characteristics such as age (continuous, and categories), body mass index (BMI; continuous and categories), treatment status (i.e., during primary treatment, following primary treatment, mixed, not treated), percentage of patients with advanced disease (stage III–IV), percentage of patients with metastasis and percentage of patients undertaking breast surgery, radiotherapy, chemotherapy and/or hormone therapy during or following primary treatment. Exercise prescription characteristics such as mode (AE, RE and/or COMB), delivery (supervised, non-supervised, hybrid), duration, frequency, dosage (or volume) and intensity were also extracted. These characteristics were categorized as below or equal to 50% of the sample, or above 50% of the sample for further analysis.

The primary outcomes were cancer-related fatigue or vitality assessed through valid questionnaires, lean mass or muscle mass measured by dual-energy x-ray absorptiometry (DEXA), plethysmography, hydrostatic weighing, D3-creatine dilution or skinfold thickness; muscle hypertrophy measured by a regional assessment of muscle hypertrophy from the lower limbs such as muscle thickness, cross-sectional area, muscle volume through muscle ultrasound, computerized tomography scans or magnetic resonance imaging; physical function measured by 30-s chair rise, chair rise test, 400-m or 6-min walk test, or timed-up and go; and lower-limb muscle strength measured by leg press and/or knee extension one-repetition maximum, or isokinetic knee extension test (isometric and dynamic at 60°.sec^−1^ and 120°.sec^−1^). Mean and dispersion values (i.e., standard deviation [SD], standard error [SE] and/or 95% confidence intervals [95% CI]) were extracted from baseline and post-assessment timepoints as well as from within- and between-group reporting. If authors of the identified articles did not include dispersion values of change, the SD of the change was calculated assuming a correlation of *r* = 0.5 between the baseline and post-intervention assessment measures [37].

### Risk of Bias Assessment and Certainty of Evidence (GRADE)

The risk of bias on the outcome level was evaluated according to the Cochrane risk-of-bias tool 2.0 (RoB 2) [38]. The study quality assessment for all included studies was performed independently by two researchers (C.T.M. and P.C.), with disagreements resolved by a third researcher (P.L.). The certainty of evidence for the network of interventions was assessed using the Grading of Recommendations Assessment, Development and Evaluation (GRADE) approach for network meta-analysis [39].

### Data Synthesis and Analysis

Continuous outcome data in both pairwise and network meta-analyses (NMA) were summarised as standardised mean differences (SMDs) and 95% confidence intervals (95% CI), except for lean mass, which was summarised as mean difference (MD) and 95% CI. These are presented in the ESM Tables S1–S4. The frequentist NMA model was performed following the current PRISMA guideline for NMA [40, 41]. All NMA analyses were conducted using the R package ‘netmeta’ [42, 43]. A random-effects model was undertaken to examine the effect of different exercise programs (i.e., aerobic, resistance, COMB) and controls, with a specific focus on the comparisons between RE and COMB. The between-study variability (i.e., heterogeneity) of the intervention effects within each intervention comparison was assessed by I^2^ and the magnitude of the between-study variance (τ^2^) and estimated using the generalised DerSimonian and Laird estimator and the Q-profile approach. For each NMA, we assessed a priori the transitivity assumption, which implies that the distribution of the potential treatment effect modifiers is balanced across the available direct comparisons [44]. We used age, BMI and overall risk of bias as potential intervention effect modifiers. We also evaluated each network for inconsistency globally using the random-effects design-by-treatment interaction model [45]. If the global test suggested inconsistency, we assessed inconsistency locally by splitting the direct and indirect evidence [46]. We assessed comparisons only when at least two studies provided data for a given exercise group. Comparisons were made when more than one study was included for each comparator. According to Hedges’ *g* [47], SMD values of 0.0 to ≤ 0.5 indicate small; 0.51–0.79, medium or moderate; and ≥ 0.8, large effects. Intervention effects were ranked according to *P scores* [48]. Extreme-study effects (i.e., outliers) were explored with the forward search (FS) algorithm [49] using the R package ‘NMAoutlier’ [50]. Obvious outliers detected were excluded in the sensitivity analysis to assess the robustness of results. A comparison-adjusted funnel plot was created to assess publication bias and small study-effects when more than ten studies were available [51].

Subgroup NMA analyses were provided for demographic and clinical characteristics such as age groups (middle-aged, older), BMI groups (overweight, obese), percentage of sample with stage III–IV (≤ 50%, > 50%), percentage of sample with metastasis (≤ 50%, > 50%), treatment status (current treatment, previous treatment), percentage of sample who had a previous breast surgery (≤ 50%, > 50%), percentage of sample who had previous radiotherapy (≤ 50%, > 50%), percentage of sample who had previous chemotherapy (≤ 50%, > 50%), percentage of sample undertaking hormone therapy (≤ 50%, > 50%), percentage of sample undergoing breast surgery (≤ 50%, > 50%), percentage of sample undergoing radiotherapy (≤ 50%, > 50%) and percentage of sample undergoing chemotherapy (≤ 50%, > 50%). For exercise prescription characteristics, subgroup NMA were provided for exercise program duration (short-term [< 24 weeks], medium-to-long term [≥ 24 weeks]), delivery (non-supervised, supervised), RE weekly volume (≤ 42 sets, > 42 sets), RE intensity (< 80% of 1-RM, ≥ 80% of 1-RM), AE mode (moderate-intensity continuous, high-intensity interval [HIIT]), AE weekly volume (< 90 min, ≥ 90 min) and AE intensity (moderate, vigorous). Given the lack of minimal clinically important difference (MCID) for the outcomes investigated, differences between RE and COMB above SMD 0.20 or MD 0.20 kg were indicative of a potential interference effect.

## Results

**Fig. 1:**
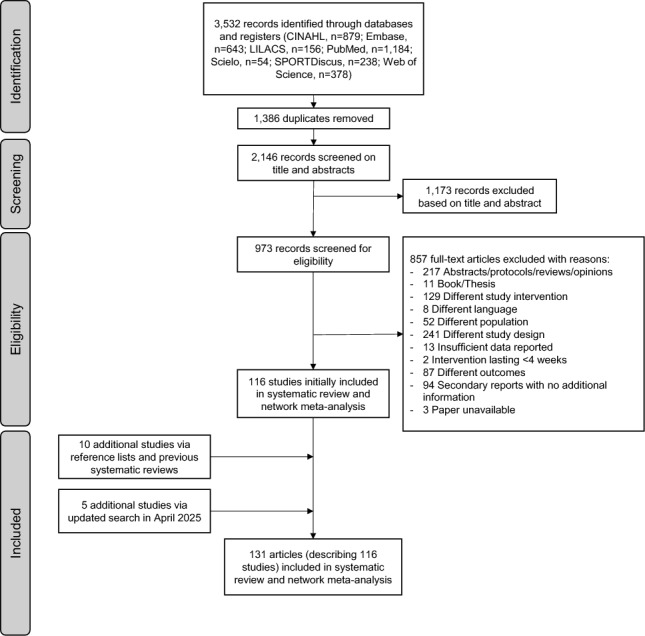
Flow chart of study selection process

A total of 3532 records were identified through databases searches. After removing duplicates, 2146 records were screened based on title and abstract. Of these, 1173 records were excluded as irrelevant to the research question, resulting in 973 records eligible for full-text review. A total of 857 reports met the exclusion criteria, while 10 [52–61] and 5 [29, 62–65] additional studies were identified through reference lists and updated search, respectively. Finally, 131 articles [16, 17, 22, 23, 29, 52–177] describing 116 randomised trials were included in this systematic review and network meta-analysis. The selection process is illustrated in Fig. 1.

### Study, Participant, and Intervention Characteristics

A total of 9206 women with breast cancer were included, with a median age of 52.4 (interquartile range [IQR] = 49.2; 55.2) years and median BMI of 27.9 (IQR = 26.0; 29.5) kg.m^−2^ at study level. The majority of the studies had included women with early-stage breast cancer (stage 0–II), while only two studies (1.7%) included patients with metastatic disease. Most studies targeted patients following primary treatment (56.9%), which commonly included breast surgery (median of 100%, IQR = 98.0%; 100%), radiotherapy (median of 78.5%, IQR = 63.6%; 90.0%) and chemotherapy (median of 77.8%, IQR = 50.9%; 94.7%). In addition, a median of 68.6% (IQR = 53.6%; 85.1%) were undergoing hormone therapy. In studies targeting patients during primary treatment (40.5%), the majority were receiving breast surgery (median of 100%, IQR = 100%; 100%), radiotherapy (median of 72.7%, IQR = 38.6%; 100%) and chemotherapy (median of 100%, IQR = 74.5%; 100%).

A total of 141 exercise programs were analysed, with the majority prescribing COMB (41.8%), followed by AE (38.3%) and RE (19.9%). The majority of the studies were supervised (62.4%) and had a median duration of 12 (IQR = 9; 16) weeks. COMB had a median of 32 (IQR = 24; 48) sessions, with 42 (IQR = 29; 52) weekly sets at 75% (IQR = 65%; 78%) of 1-RM of RE and 90 (IQR = 64; 146) min per week at moderate-to-vigorous intensity of AE (54.4%). AE alone had a median of 34 (IQR = 24; 36) sessions, with 90 (IQR = 70; 120) min at moderate-to-vigorous intensity of AE (58.5%), while for RE alone, a median of 36 (IQR = 24; 52) sessions, with 45 (IQR = 36; 60) weekly sets at 75% (IQR = 70%; 80%) of 1-RM was prescribed. Most control groups were non-intervention, usual care or waitlist/delayed control groups (87.1%), while the remaining had some type of minimal exposure such as stretching, relaxation, education, physiotherapy and physical activity counselling. The characteristics of the individual studies are presented in ESM Table S5. The risk of bias assessment for each outcome is presented in ESM Tables S6–S9.

### Fatigue

#### Main Model

Both RE [number of comparisons (*k*) = 79; SMD − 0.52, 95% CI − 0.83 to − 0.21, *p* = 0.001; *P*-score = 80.8%] and COMB (*k* = 79; SMD − 0.47, 95% CI − 0.65 to − 0.29, *p* < 0.001; *p-*score = 74.8%) significantly reduced cancer-related fatigue compared with controls (Table 1). AE also significantly reduced cancer-related fatigue compared with controls (SMD − 0.32, 95% CI − 0.55 to − 0.10, *p* = 0.005). Differences between RE and COMB (SMD 0.05, 95% CI − 0.31 to 0.41, *p* = 0.791) and RE and AE (SMD − 0.19, 95% CI − 0.56 to 0.17, *p* = 0.299) were not statistically significant in the overall analysis. The certainty of evidence was graded very low. The heterogeneity I^2^ was 87%. The design-by-treatment interaction model showed no evidence of statistically significant inconsistency in the NMA for fatigue (*Q* = 0.74, *p* = 0.981). Local side-split analyses are presented in ESM Table S10. Visual assessment of comparison-adjusted funnel plots (ESM Fig. S1) suggested evidence of small-study effects (*p* = 0.054), while outliers were not detected by the FS method.

**Table 1:** Network meta-analysis results for fatigue

Comparisons	SMD (95% CI)	*P*-value	I^2^	*P*-score	Certainty
**Fatigue**^**a**^	***k* = 79**				
AE versus CTR	− 0.32 (− 0.55 to − 0.10)	0.005	87%	RE: 81.8% COMB: 74.8% AE: 43.3%	⊕ ⊝ ⊝ ⊝ Very low^b,c,d^
RE versus CTR	− 0.52 (− 0.83 to − 0.21)	0.001			
COMB versus CTR	− 0.47 (− 0.65 to − 0.29)	< 0.001			
AE versus RE	0.19 (− 0.17 to 0.56)	0.299			
AE versus COMB	0.15 (− 0.13 to 0.42)	0.303			
RE versus COMB	0.05 (− 0.31 to 0.41)	0.791			

*95% CI* 95% confidence interval, *AE* aerobic exercise, *COMB* combined resistance and aerobic exercise, *CTR* controls, *k* number of comparisons, *RE* resistance exercise, *SMD* standardised mean difference

^a^Lower values indicate better outcome

^b^Certainty of evidence downgraded due to study limitations, with most studies (> 50%) presenting with high risk in the risk of bias assessment

^c^Certainty of evidence downgraded due to imprecision, with confidence intervals from interventions crossing null values or including values favouring both interventions tested

^d^Certainty of evidence downgraded due to publication bias, with visual assessment of comparison-adjusted funnel plots suggesting evidence of small-study effects

#### Subgroup Analyses

**Fig. 2:**
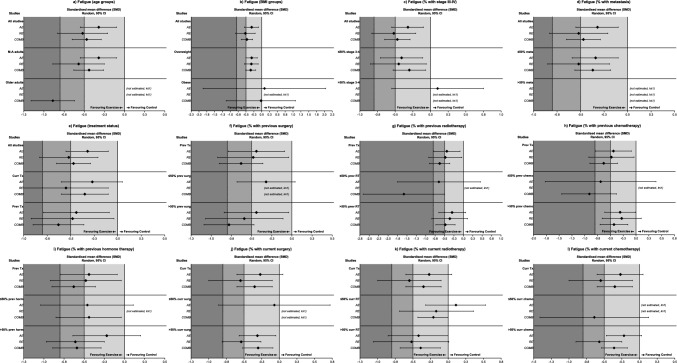
Subgroup network meta-analysis of fatigue on the basis of **a** age groups, **b** body mass index groups [BMI], **c** % with stage III–IV, **d** % with metastasis, **e** treatment status, **f** % with previous surgery, **g** % with previous radiotherapy, **h** % with previous chemotherapy, **i** % with previous hormone therapy, **j** % with current surgery, (k) % with current radiotherapy and **l** % with current chemotherapy. Light-grey shade indicates small effect size; grey shade indicates medium effect size; dark-grey shade indicates large effect size. *AE* aerobic exercise, *chemo* chemotherapy, *COMB* combined resistance and aerobic exercise, *curr* current, *M-A* middle-aged adults, *mets* metastasis, *prev* previous, *RE* resistance exercise, *RT* radiotherapy, *surg* surgery, *Tx* treatment

**Fig. 3:**
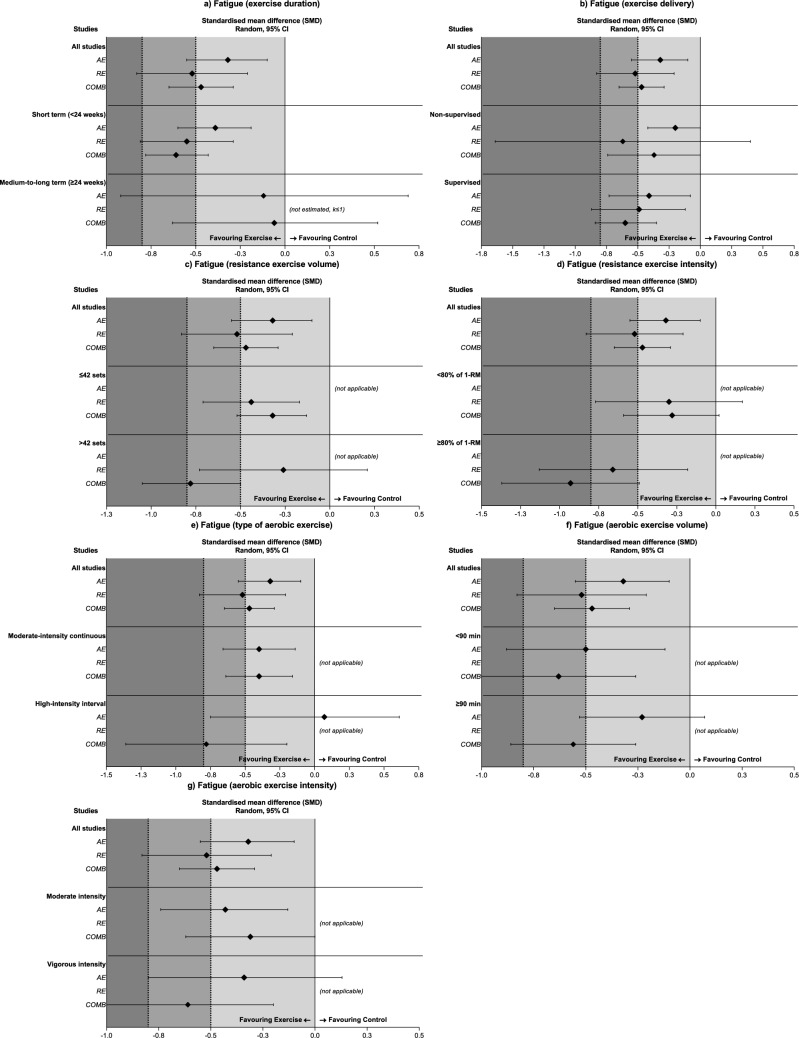
Subgroup network meta-analysis of fatigue on the basis of **a** exercise duration, **b** exercise delivery, **c** resistance exercise volume, **d** resistance exercise intensity, **e** type of aerobic exercise, **f** aerobic exercise volume and **g** aerobic exercise intensity. Light-grey shade indicates small effect size; grey shade indicates medium effect size; dark-grey shade indicates large effect size. *AE* aerobic exercise, *COMB* combined resistance and aerobic exercise, *RE* resistance exercise

A potential interference effect (SMD <  − 0.20) between RE and COMB was observed on fatigue under specific circumstances, particularly in patients undertaking exercise during primary treatment (subgroup differences, *p* = 0.399; RE versus COMB = SMD − 0.20, 95% CI − 0.71 to 0.31, *p* = 0.441; Fig. 2e) and receiving breast surgery (subgroup differences, *p* = 0.410; RE versus COMB = SMD − 0.23, 95% CI − 0.58 to 0.12, *p* = 0.191; Fig. 2j) or chemotherapy (subgroup differences, *p* = 0.238; RE versus COMB = SMD − 0.22, 95% CI − 0.59 to 0.16, *p* = 0.257; Fig. 2l). An interference effect was also observed when delivering non-supervised exercise programs (subgroup differences, *p* = 0.248; RE versus COMB = SMD − 0.25, 95% CI − 1.33 to 0.84, *p* = 0.654; Fig. 3b). Conversely, an interference effect on fatigue was mitigated when prescribing > 42 RE weekly sets (subgroup differences, *p* = 0.639; RE versus COMB = SMD 0.52, 95% CI − 0.02 to 1.06, *p* = 0.061; Fig. 3c) or at least 80% of 1-RM (subgroup differences, *p* = 0.278; RE versus COMB = SMD 0.27, 95% CI − 0.38 to 0.92, *p* = 0.411; Fig. 3d) within COMB, prescribing moderate-intensity continuous AE rather than HIIT (subgroup differences, *p* = 0.529; SMD 0.53, 95% CI 0.03 to 1.03, *p* = 0.038; Fig. 3e) or prescribing vigorous intensity rather than moderate intensity continuous AE (subgroup differences, *p* = 0.590; SMD 0.30, 95% CI − 0.21 to 0.81, *p* = 0.253; Fig. 3g). An interference effect was unlikely to be meaningful in the other subgroup comparisons.

### Lean Mass

#### Main Model

RE was the most effective intervention to significantly increase lean mass (*k* = 29, MD 0.68 kg, 95% CI 0.43 to 0.94 kg, *p* < 0.001) compared with controls, with a *p*-score equal to 92.8% (Table 2). Although RE provided larger effects compared with COMB (MD 0.18 kg, 95% CI − 0.17 to 0.53 kg, *p* = 0.303) and AE (MD 0.26 kg, 95% CI − 0.07 to 0.59 kg, *p* = 0.129), differences were not statistically significant. The certainty of evidence was graded very low. The heterogeneity I^2^ was 43%, with evidence of small-study effects (*p* = 0.031; ESM Fig. S2). In addition, the design-by-treatment interaction model showed no evidence of statistically significant inconsistency in the NMA for lean mass (*Q* = 1.97, *p* = 0.742). Local side-split analyses are presented in ESM Table S11. The FS algorithm identified two outliers [129, 130, 161] in lean mass (ESM Fig. S3). Sensitivity analysis omitting the outliers resulted in slightly different effects for AE (MD 0.39 kg, 95% CI 0.14–0.64 kg, *p* = 0.002) and COMB (MD 0.42 kg, 95% CI 0.27–0.58 kg, *p* < 0.001), while a larger effect was found for RE (MD 0.78 kg, 95% CI 0.63–0.93 kg, *p* < 0.001) with a *P*-score of 99.9%. Differences between RE and COMB (MD 0.36 kg, 95% CI 0.15–0.57 kg, *p* < 0.001) and RE and AE (MD 0.39 kg, 95% CI 0.13–0.65 kg, *p* < 0.001) were also relevant. The heterogeneity reduced from 40% in the primary analysis to 12% (ESM Table S12).

**Table 2:** Network meta-analysis results for lean mass

Comparisons	MD (95% CI)	*p*-value	*I*^2^	*P*-score	Certainty
**Lean mass**	***k* = 29**				
AE versus CTR	0.43 (0.12 to 0.73)	0.007	43%	RE: 92.8% COMB: 60.4% AE: 46.7%	⊕ ⊝ ⊝ ⊝ Very low^a,b,c^
RE versus CTR	0.68 (0.43 to 0.94)	< 0.001			
COMB versus CTR	0.50 (0.24 to 0.76)	< 0.001			
AE versus RE	− 0.26 (− 0.59 to 0.07)	0.129			
AE versus COMB	− 0.08 (− 0.43 to 0.28)	0.677			
RE versus COMB	0.18 (− 0.17 to 0.53)	0.303			

*95% CI* 95% confidence interval, *AE* aerobic exercise, *COMB* combined resistance and aerobic exercise, *CTR* controls, *k* number of comparisons, *RE* resistance exercise, *MD* mean difference

^a^Certainty of evidence downgraded due to study limitations, with most studies (> 50%) presenting with high risk in the risk of bias assessment

^b^Certainty of evidence downgraded due to imprecision, with confidence intervals from interventions crossing null values or including values favouring both interventions tested

^c^Certainty of evidence downgraded due to publication bias, with visual assessment of comparison-adjusted funnel plots suggesting evidence of small-study effects

#### Subgroup Analyses

**Fig. 4:**
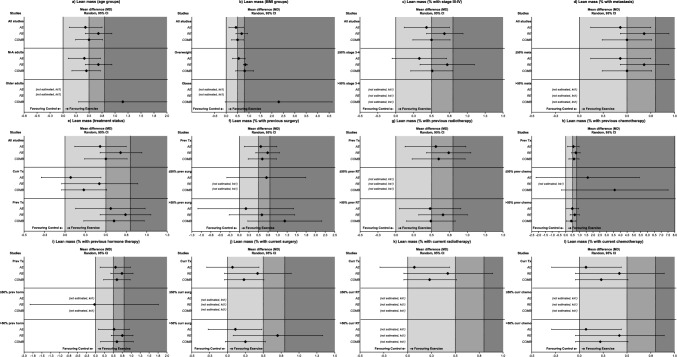
Subgroup network meta-analysis of lean mass based on **a** age groups, **b** body mass index groups [BMI], **c** % with stage III–IV, **d** % with metastasis, **e** treatment status, **f** % with previous surgery, **g** % with previous radiotherapy, **h** % with previous chemotherapy, **i** % with previous hormone therapy, **j** % with current surgery, **k** % with current radiotherapy and **l** % with current chemotherapy. Light-grey shade indicates small effect size; grey shade indicates medium effect size; dark-grey shade indicates large effect size. *AE* aerobic exercise, *chemo* chemotherapy, *COMB* combined resistance and aerobic exercise, *curr* current, *M-A* middle-aged adults, *mets* metastasis, *prev* previous, *RE* resistance exercise, *RT* radiotherapy, *surg* surgery, *Tx* treatment

**Fig. 5:**
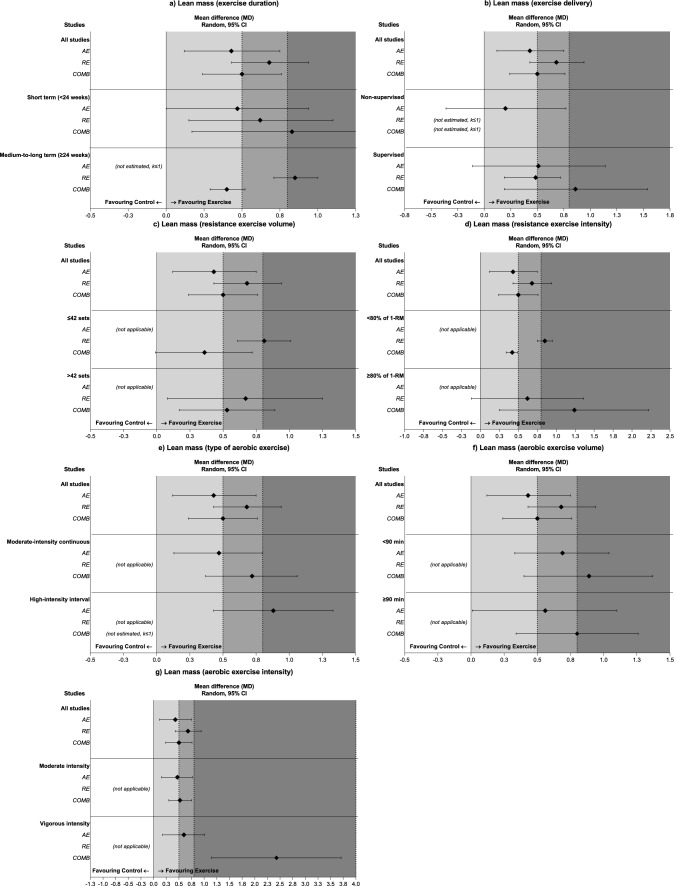
Subgroup network meta-analysis of lean mass based on **a** exercise duration, **b** exercise delivery, **c** resistance exercise volume, **d** resistance exercise intensity, **e** type of aerobic exercise, **f** aerobic exercise volume and **g** aerobic exercise intensity. Light-grey shade indicates small effect size; grey shade indicates medium effect size; dark-grey shade indicates large effect size. *AE* aerobic exercise, *COMB* combined resistance and aerobic exercise, *RE* resistance exercise

This potential interference effect (MD > 0.20 kg) between RE and COMB was prominent in middle-aged patients (subgroup differences, *p* = 0.404; RE versus COMB = MD 0.24, 95% CI − 0.14 to 0.61, *p* = 0.217; Fig. 4a), patients undertaking exercise during primary treatment and receiving breast surgery (subgroup differences, *p* = 0.582; RE versus COMB = MD 0.46, 95% CI − 0.23 to 1.14, *p* = 0.191; Fig. 4j) or chemotherapy (subgroup differences, *p* = 0.421; RE versus COMB = MD 0.20, 95% CI − 0.35 to 0.73, *p* = 0.489; Fig. 4l), and patients undertaking exercise following primary treatment who had received chemotherapy (subgroup differences, *p* = 0.193; RE versus COMB = MD 0.27, 95% CI − 0.22 to 0.76, *p* = 0.283; Fig. 4h). In regard to exercise prescription characteristics, a potential interference effect was noted in medium-to-long exercise duration (subgroup differences, *p* = 0.078; RE versus COMB = MD 0.45, 95% CI 0.26–0.63, *p* < 0.001; Fig. 5a), prescribing ≤ 42 weekly sets (subgroup differences, *p* = 0.073; RE versus COMB = MD 0.45, 95% CI 0.03–0.87, *p* = 0.034; Fig. 5c) or at RE intensities < 80% of 1-RM within COMB (subgroup differences, *p* < 0.001; RE versus COMB = MD 0.44, 95% CI 0.32–0.56, *p* < 0.001; Fig. 5c). In contrast, the potential interference effect on lean mass was overcome in patients undertaking exercise following primary treatment who had received breast surgery (subgroup differences, *p* = 0.267; RE versus COMB = MD − 0.60, 95% CI − 0.70 to 1.90, *p* = 0.366; Fig. 4f), in short-term exercise duration (subgroup differences, *p* = 0.078; RE versus COMB = MD − 0.20, 95% CI − 1.01 to 0.60, *p* = 0.623; Fig. 5a), when prescribing the RE component at intensities ≥ 80% of 1-RM (subgroup differences, *p* = 0.775; RE versus COMB = MD − 0.62, 95% CI − 1.85 to 0.61, *p* = 0.325; Fig. 5c) or when prescribing the AE component at vigorous intensity rather than moderate intensity continuous AE (subgroup differences, *p* = 0.217; MD − 1.91, 95% CI − 3.21 to − 0.61, *p* = 0.004; Fig. 5g). An interference effect was unlikely to be meaningful in the other subgroup comparisons.

### Physical Function

#### Main Model

RE (*k* = 35; SMD 0.86, 95% CI 0.41 to 1.30, *p* < 0.001; *P*-score = 75.8%) and COMB (*k* = 35; SMD 0.90, 95% CI 0.58 to 1.22, *p* < 0.001; *P*-score = 80.8%) significantly improved physical function compared with controls (Table 3). AE also resulted in significant improvements in physical function compared with controls (SMD 0.59, 95% CI 0.15–1.03, *p* = 0.009). Differences between COMB and RE (SMD − 0.04, 95% CI − 0.58 to 0.50, *p* = 0.880) and RE and AE (SMD − 0.27, 95% CI − 0.80 to 0.27, *p* = 0.335) were not statistically significant. Specific results for timed-up and go, 30-s chair rise, sit-to-stand and walking distance tests are provided in ESM Tables S13–S16. The certainty of evidence was graded low. The heterogeneity I^2^ was 88%. Visual assessment of comparison-adjusted funnel plots (ESM Fig. S4) did not indicate small-study effects (*p* = 0.108). The design-by-treatment interaction model showed no evidence of statistically significant inconsistency in the NMA for physical function (*Q* = 3.66, *p* = 0.600). Local side-split analyses are presented in ESM Table S17. The FS algorithm provided two outliers [150, 151] in physical function. Sensitivity analysis omitting the outliers resulted in slightly smaller effects for AE (SMD 0.52, 95% CI 0.21–0.83, *p* = 0.001), RE (SMD 0.62, 95% CI 0.29–0.95, *p* < 0.001) and COMB (SMD 0.73, 95% CI 0.50–0.97, *p* < 0.001). The heterogeneity reduced from 88% in the primary analysis to 75% (ESM Table S18).

**Table 3:** Network meta-analysis results for physical function outcomes

Comparisons	SMD (95% CI)	*p*-value	*I*^2^	*P*-score	Certainty
**Physical function**	***K* = 35**				
AE versus CTR	0.59 (0.15 to 1.03)	0.009	88%	COMB: 80.8% RE: 75.8% AE: 43.3%	⊕ ⊕ ⊝ ⊝ Low^a,b^
RE versus CTR	0.86 (0.41 to 1.30)	< 0.001			
COMB versus CTR	0.90 (0.58 to 1.22)	< 0.001			
AE versus RE	− 0.27 (− 0.80 to 0.27)	0.335			
AE versus COMB	− 0.31 (− 0.85 to 0.24)	0.270			
RE versus COMB	− 0.04 (− 0.58 to 0.50)	0.880			

*95% CI* 95% confidence interval, *AE* aerobic exercise, *COMB* combined resistance and aerobic exercise, *CTR* controls, *k* number of comparisons, *RE* resistance exercise, *SMD* standardised mean difference

^a^Certainty of evidence downgraded due to study limitations, with most studies (> 50%) presenting with high risk in the risk of bias assessment

^b^Certainty of evidence downgraded due to imprecision, with confidence intervals from interventions crossing null values or including values favouring both interventions tested

#### Subgroup Analyses

**Fig. 6:**
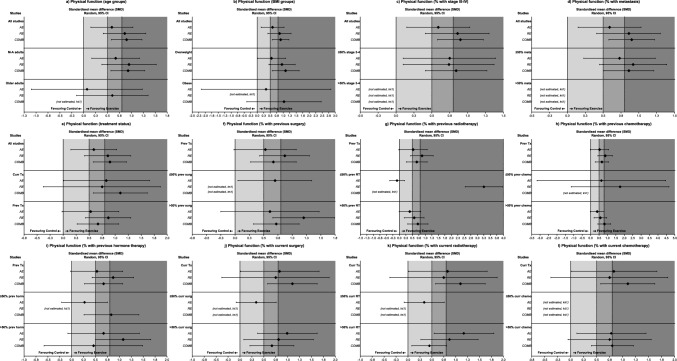
Subgroup network meta-analysis of physical function based on **a** age groups, **b** body mass index groups [BMI], **c** % with stage III–IV, **d** % with metastasis, **e** treatment status, **f** % with previous surgery, **g** % with previous radiotherapy, **h** % with previous chemotherapy, **i** % with previous hormone therapy, **j** % with current surgery, **k** % with current radiotherapy and **l** % with current chemotherapy. Light-grey shade indicates small effect size; grey shade indicates medium effect size; dark-grey shade indicates large effect size. *AE* aerobic exercise, *chemo* chemotherapy, *COMB* combined resistance and aerobic exercise, *curr* current, *M-A* middle-aged adults, *mets* metastasis, *prev* previous, *RE* resistance exercise, *RT* radiotherapy, *surg* surgery, *Tx* treatment

**Fig. 7:**
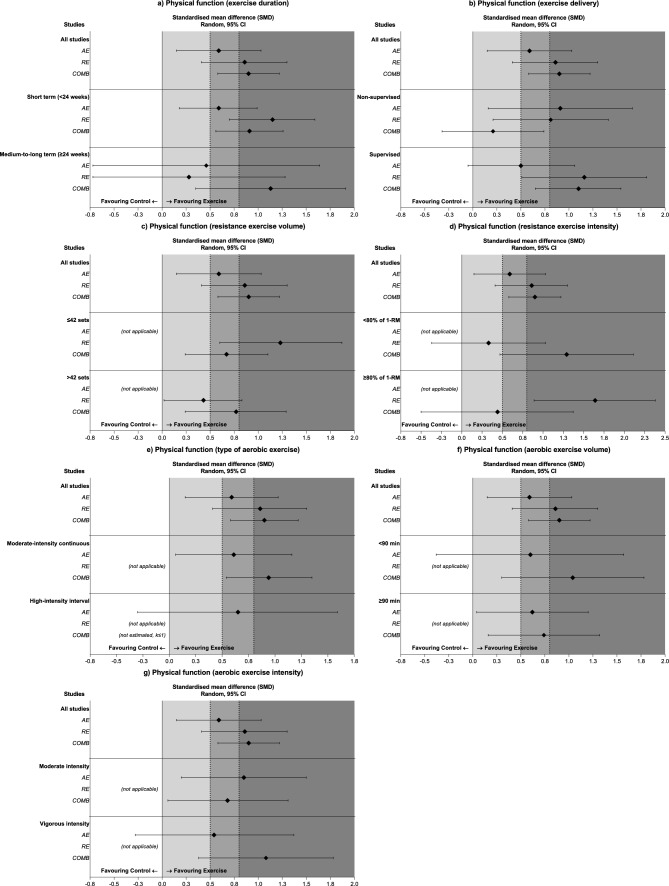
Subgroup network meta-analysis of physical function based on **a** exercise duration, **b** exercise delivery, **c** resistance exercise volume, **d** resistance exercise intensity, **e** type of aerobic exercise, **f** aerobic exercise volume and **g** aerobic exercise intensity. Light-grey shade indicates small effect size; grey shade indicates medium effect size; dark-grey shade indicates large effect size. *AE* aerobic exercise, *COMB* combined resistance and aerobic exercise, *RE* resistance exercise

A potential interference effect (SMD > 0.20) between RE and COMB was observed in patients undertaking exercise during primary treatment and receiving radiotherapy (subgroup differences, *p* = 0.404; RE versus COMB = SMD 0.43, 95% CI − 0.27 to 1.13, *p* = 0.232; Fig. 6k), patients undertaking exercise following primary treatment (subgroup differences, *p* = 0.288; RE versus COMB = SMD 0.20, 95% CI − 0.40 to 0.79, *p* = 0.520; Fig. 6e) and who had received breast surgery (subgroup differences, *p* = 0.814; RE versus COMB = SMD 0.48, 95% CI − 0.20 to 1.15, *p* = 0.165; Fig. 6f) and hormone therapy (subgroup differences, *p* = 0.199; RE versus COMB = SMD 0.62, 95% CI − 0.56 to 1.81, *p* = 0.303; Fig. 6i). In addition, the potential interference effect was noted in short term duration exercise (subgroup differences, *p* = 0.725; RE versus COMB = SMD 0.23, 95% CI − 0.33 to 0.80, *p* = 0.415; Fig. 7a), delivering non-supervised exercise programs (subgroup differences, *p* = 0.847; RE versus COMB = SMD 0.60, 95% CI − 0.20 to 1.40, *p* = 0.140; Fig. 7b), prescribing ≤ 42 RE weekly sets (subgroup differences, *p* = 0.204; RE versus COMB = SMD 0.56, 95% CI − 0.20 to 1.33, *p* = 0.147; Fig. 7c) or prescribing the RE at intensities ≥ 80% of 1-RM within COMB (subgroup differences, *p* = 0.216; RE versus COMB = SMD 1.20, 95% CI 0.01 to 2.39, *p* = 0.049; Fig. 7d). In contrast, the interference effect on physical function was overcome in patients undertaking exercise during primary treatment (subgroup differences, *p* = 0.288; RE versus COMB = SMD − 0.35, 95% CI − 1.59 to 0.89, *p* = 0.577; Fig. 6e), patients undertaking exercise following primary treatment who had received chemotherapy (subgroup differences, *p* = 0.733; RE versus COMB = SMD − 0.24, 95% CI − 0.78 to 0.31, *p* = 0.390; Fig. 6h), medium-to-long term duration exercise (subgroup differences, *p* = 0.725; RE verus COMB = SMD − 0.85, 95% CI − 2.11 to 0.42, *p* = 0.190; Fig. 7a), prescribing > 42 RE weekly sets (subgroup differences, *p* = 0.610; RE versus COMB = SMD − 0.34, 95% CI − 1.00 to 0.33, *p* = 0.318; Fig. 7c), prescribing the RE component at intensities < 80% of 1-RM (subgroup differences, *p* = 0.859; RE versus COMB = SMD − 0.96, 95% CI − 2.04 to 0.11, *p* = 0.079; Fig. 7d), prescribing < 90 min rather than ≥ 90 min of weekly AE (subgroup differences, *p* = 0.334; SMD − 0.30, 95% CI − 1.24 to 0.64, *p* = 0.532; Fig. 7f) or prescribing the AE component at vigorous intensity rather than moderate intensity (subgroup differences, *p* = 0.211; SMD − 0.40, 95% CI − 1.34 to 0.54, *p* = 0.405; Fig. 7g). An interference effect was unlikely to be meaningful in the other subgroup comparisons.

### Muscle Strength

#### Main Model

RE was the most effective intervention to significantly improve muscle strength (*k* = 33, SMD 1.01, 95% CI 0.71–1.32, *p* < 0.001) compared with controls, with a 98.9% probability of being the most effective intervention (Table 4). RE showed superior effects compared with AE (SMD 0.57, 95% CI 0.13–1.00, *p* = 0.011), and approached statistical significance compared with COMB (SMD 0.43, 95% CI − 0.00 to 0.86, *p* = 0.052). COMB (SMD 0.59, 95% CI 0.27 to 0.90, *p* < 0.001) and AE (SMD 0.45, 95% CI 0.04–0.85, *p* = 0.032) also led to improvements in muscle strength. Specific results for leg press 1-RM, leg extension 1-RM and isometric knee extension are provided in ESM Tables S19–S21. The certainty of evidence was graded moderate. The heterogeneity I^2^ was 86%. Visual assessment of comparison-adjusted funnel plots (ESM Fig. S5) suggested no evidence of small-study effects (*p* = 0.345). In addition, the design-by-treatment interaction model showed no evidence of statistically significant inconsistency in the NMA for muscle strength (*Q* = 5.56, *p* = 0.475). Local side-split analyses are presented in ESM Table S22. The FS algorithm identified two outliers [129, 130, 139, 146] in muscle strength. Sensitivity analysis omitting the outliers resulted in attenuated results for AE (SMD 0.31, 95% CI 0.02–0.60, *p* = 0.038), COMB (SMD 0.39, 95% CI 0.16–0.62, *p* = 0.001) and RE (SMD 0.78, 95% CI 0.56–1.01, *p* < 0.001). Differences between RE and COMB were also relevant (SMD 0.40, 95% CI 0.08–0.71, *p* = 0.014). The heterogeneity reduced from 86% in the primary analysis to 71% (ESM Table S23).

**Table 4:** Network meta-analysis results for lower-limb muscle strength

Comparisons	SMD (95% CI)	*p*-value	*I*^2^	*P*-score	Certainty
**Muscle strength**	***k* = 33**				
AE versus CTR	0.45 (0.04 to 0.85)	0.032	86%	RE: 98.9% COMB: 58.2% AE: 42.3%	⊕ ⊕ ⊕ ⊝ Moderate^a^
RE versus CTR	1.01 (0.71 to 1.32)	< 0.001			
COMB versus CTR	0.59 (0.27 to 0.90)	< 0.001			
AE versus RE	− 0.57 (− 1.00 to − 0.13)	0.011			
AE versus COMB	− 0.14 (− 0.61 to 0.33)	0.558			
RE versus COMB	0.43 (− 0.00 to 0.86)	0.052			

*95% CI* 95% confidence interval, *AE* aerobic exercise, *COMB* combined resistance and aerobic exercise, *CTR* controls, *k* number of comparisons; RE, resistance exercise; SMD, standardised mean difference

^a^Certainty of evidence downgraded due to study limitations, with most studies (> 50%) presenting with high risk in the risk of bias assessment

#### Subgroup Analyses

**Fig. 8:**
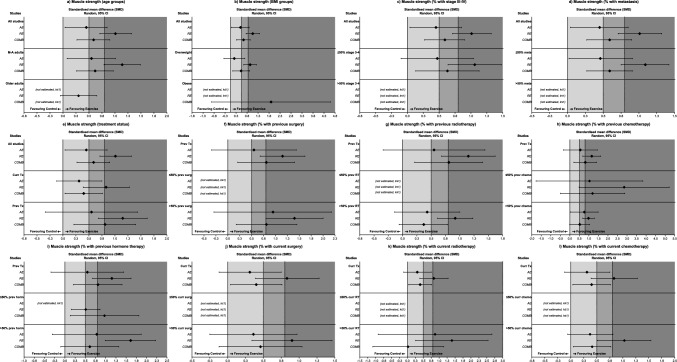
Subgroup network meta-analysis of muscle strength based on **a** age groups, **b** body mass index groups [BMI], **c** % with stage III–IV, **d** % with metastasis, **e** treatment status, **f** % with previous surgery, **g** % with previous radiotherapy, **h** % with previous chemotherapy, **i** % with previous hormone therapy, **j** % with current surgery, **k** % with current radiotherapy and **l** % with current chemotherapy. Light-grey shade indicates small effect size; grey shade indicates medium effect size; dark-grey shade indicates large effect size. *AE* aerobic exercise, *chemo* chemotherapy, *COMB* combined resistance and aerobic exercise, *curr* current, *M-A* middle-aged adults, *mets* metastasis, *prev* previous, *RE* resistance exercise, *RT* radiotherapy, *surg* surgery, *Tx* treatment

**Fig. 9:**
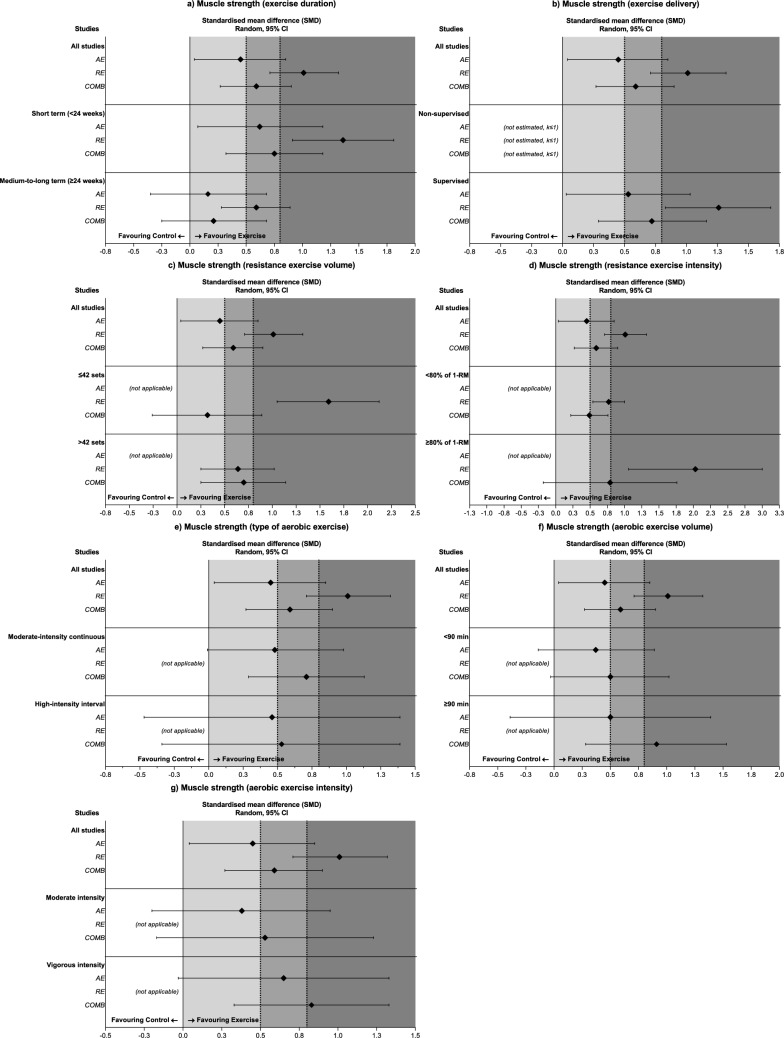
Subgroup network meta-analysis of muscle strength based on **a** exercise duration, **b** exercise delivery, **c** resistance exercise volume, **d** resistance exercise intensity, **e** type of aerobic exercise, **f** aerobic exercise volume and **g** aerobic exercise intensity. Light-grey shade indicates small effect size; grey shade indicates medium effect size; dark-grey shade indicates large effect size. *AE* aerobic exercise, *COMB* combined resistance and aerobic exercise, *RE* resistance exercise

A potential interference effect (> 0.20 SMD) between RE and COMB was observed in middle-aged patients (subgroup differences, *p* = 0.582; RE versus COMB = SMD 0.52, 95% CI 0.02–1.02, *p* = 0.041; Fig. 8a), overweight patients (subgroup differences, *p* = 0.059; RE versus COMB = SMD 0.41, 95% CI − 0.08 to 0.89, *p* = 0.103; Fig. 8b), patients undertaking exercise during primary treatment and receiving breast surgery (subgroup differences, *p* = 0.014; RE versus COMB = SMD 0.44, 95% CI − 0.36 to 1.25, *p* = 0.280; Fig. 8j), radiotherapy (subgroup differences, *p* = 0.121; RE versus COMB = SMD 1.38, 95% CI − 0.21 to 2.98, *p* = 0.089; Fig. 8k), chemotherapy (subgroup differences, *p* = 0.655; RE versus COMB = SMD 0.61, 95% CI − 0.01 to 1.24, *p* = 0.055; Fig. 8l) or patients undertaking exercise following primary treatment who had received breast surgery (subgroup differences, *p* = 0.099; RE versus COMB = SMD 0.59, 95% CI − 0.28 to 1.46, *p* = 0.184; Fig. 8f), radiotherapy (subgroup differences, *p* = 0.052; RE versus COMB = SMD 0.55, 95% CI 0.03–1.08, *p* = 0.040; Fig. 8g), chemotherapy (subgroup differences, *p* = 0.052; RE versus COMB = SMD 0.47, 95% CI − 0.14 to 1.07, *p* = 0.131; Fig. 8h) or hormone therapy (subgroup differences, *p* = 0.108; RE versus COMB = SMD 1.00, 95% CI 0.06–1.94, *p* = 0.038; Fig. 8i). Regarding exercise prescription characteristics, the interference effect was observed in short (subgroup differences, *p* = 0.380; RE versus COMB = SMD 0.61, 95% CI − 0.01 to 1.24, *p* = 0.054) and medium-to-long duration (SMD 0.37, 95% CI − 0.18 to 0.93, *p* = 0.189; Fig. 9a), in supervised exercise programs (subgroup differences, *p* = 0.414; RE versus COMB = SMD 0.53, 95% CI − 0.08 to 1.14, *p* = 0.087; Fig. 9b), prescribing ≤ 42 RE weekly sets (subgroup differences, *p* = 0.363; RE versus COMB = SMD 1.27, 95% CI 0.49 2.05, *p* = 0.002; Fig. 9c) or prescribing the RE component at intensities < 80% of 1-RM (subgroup differences, *p* = 0.210; RE versus COMB = SMD 0.28, 95% CI − 0.07 to 0.64, *p* = 0.118) and ≥ 80% of 1-RM within COMB (subgroup differences, *p* = 0.203; RE versus COMB = SMD 1.24, 95% CI − 0.13 to 2.61, *p* = 0.076; Fig. 9d). The interference effect on muscle strength was mitigated when prescribing ≥ 90 min rather than < 90 min of weekly AE (subgroup differences, *p* = 0.003; RE versus COMB = SMD − 0.41, 95% CI − 1.22 to 0.40, *p* = 0.321; Fig. 9f) or when prescribing the AE component at vigorous intensity rather than moderate intensity (subgroup differences, *p* = 0.010; RE versus COMB = SMD − 0.30, 95% CI − 1.15 to 0.55, *p* = 0.494; Fig. 9g). An interference effect was unlikely to be meaningful in the other subgroup comparisons.

## Discussion

In the present systematic review and network meta-analysis, we examined the effects of COMB, RE alone and AE alone on fatigue, lean mass, physical function and muscle strength to investigate the presence of an interference effect from prescribing COMB in women with breast cancer diagnosed with any type, stage and treatment. In addition, we examined a range of demographic, clinical and exercise prescription moderators in this group. While the interference effect was not observed on fatigue and physical function in the overall analysis, prescribing COMB may result in attenuated benefits in certain clinical groups and exercise prescription characteristics. Conversely, a potential interference effect was more pronounced on lean mass and muscle strength and remained consistent across a range of demographic, clinical and exercise prescription characteristics. AE alone resulted in smaller and modest effects on the outcomes investigated compared with RE and COMB. These findings highlight the importance of delivering exercise programs based on cancer treatment characteristics and exercise goals when prescribing RE and AE for women with breast cancer.

Fatigue is one of the most reported [2, 3] and impactful symptoms for women with breast cancer [4]. Exercise programs are well-established as effective in alleviating cancer-related fatigue [178, 179], though the magnitude of these effects is perhaps limited [178, 179]. Consistent with previous meta-analyses [179–181], we observed moderate exercise effects (SMD ~ 0.5) on fatigue, even after controlling for multiple comparisons in the network meta-analysis. COMB, however, resulted in a potential interference effect when undertaken specifically after breast surgery or during adjuvant chemotherapy. This may be owing to the excessive workload imposed by combining RE and AE within sessions, cumulative residual fatigue from repeated exercise sessions [28], or different physiological and inflammatory responses to RE and AE that could affect fatigue [182]. In addition, the smaller effect observed for AE alone may further support these mechanisms [28, 182]. Adjuvant chemotherapy, which is widely used [183], is associated with exacerbated fatigue symptoms compared with other regimens [184], further supporting the use of RE alone in this context. While undertaking a supervised COMB program may overcome this potential interference effect, increasing RE dosage and intensity or increasing AE intensity within COMB may be impractical due to the patients’ deconditioning and symptom burden during primary treatment [185]. Therefore, RE alone may offer greater reductions in fatigue rather than combined with AE or AE alone during adjuvant chemotherapy. Following primary treatment, COMB or AE may be necessary to achieve additional benefits for fatigue and other markers of cardiorespiratory fitness.

Declines in lean body mass ranging from − 1.7 to − 0.4 kg are common during and after chemotherapy, radiotherapy and hormone therapy [186], with low lean mass associated with a ~ 40% higher risk of mortality [5, 187]. This phenomenon is considered an accelerated ageing process [188], hypothesised to result from proinflammatory cytokines induced by radiotherapy [189] and telomere shortening caused by chemotherapy [190]. RE, despite its limited benefit in the setting of breast cancer [191], provides an undeniable anabolic effect [191], counteracting expected declines of − 0.6 kg in lean mass during usual care [191]. Higher levels of lean mass may also contribute to the metabolism and clearance of chemotherapy agents while protecting against treatment toxicities [192]. Therefore, maintaining and accruing lean mass is critical for women with breast cancer [193]. Indeed, we observed a moderate-to-large effect on lean mass, with a median increase of 0.8 kg. While RE is associated with clear benefits, the combination with AE or AE alone requires careful consideration when targeting lean mass in women with breast cancer. Our analyses indicated that COMB diminished the benefits of RE on lean mass, with both COMB and AE showing SMD values approximately half that of RE alone, as confirmed by the sensitivity analysis. This finding was consistent across a range of demographic, clinical and exercise prescription characteristics. This phenomenon may be explained by the compromised immune system following cancer treatment [194, 195], which affects the capacity to recover and adapt to the excessive workload imposed by combining RE and AE. This was also hypothesised in a previous exercise trial in patients with glioblastoma undertaking adjuvant chemoradiotherapy [30]. In circumstances where reversing loss of lean mass is critical to prevent sarcopenia, frailty and cachexia, such as during and following chemotherapy [186], a targeted RE prescription should be emphasized to maximize lean mass retention and mitigate risks associated with treatment-induced loss of lean mass.

The fatigue, loss of lean mass and mobility limitations experienced by women with breast cancer during treatment may impact the ability to undertake activities of daily living [196]. These may affect participation in social and occupational activities, resulting in social isolation and decreased quality of life. We observed that either COMB or RE resulted in large and similar effects on the analyses combining the different physical function tests, whereas AE yielded smaller effects. However, results were inconsistent when analysing each physical function test separately. RE was the most effective program for improving the 30-s chair rise, sit-to-stand test and walking distance tests (400-m walk and 6-min walking test), with differences above SMD 0.2 compared with COMB or AE. This potential interference effect, where COMB diminished the benefits of RE alone, was observed during primary treatment (chemotherapy or radiotherapy), following primary treatment (breast surgery or hormone therapy) and further supported by the smaller effects derived from AE alone. This information is important for patients following primary treatment as hormonal therapy, specifically aromatase inhibitors, is associated with a loss of bone mineral density and increased risk of falls and fractures [197, 198]. Therefore, RE is critical for preserving physical function during primary treatment or reversing physical disability afterward, as higher levels of physical function are independently associated with ~ 50% lower all-cause mortality in older adults with cancer [6].

Muscle strength is one of the most studied components of physical fitness. A recently published meta-analysis by Bettariga et al. [7] found that higher muscle strength levels are associated with a ~ 30% reduction in the risk of all-cause mortality after a cancer diagnosis, underscoring the importance of this marker in patients with breast cancer. Following the principle of *specificity*, we found that RE was the most effective intervention for increasing muscle strength in this population. Interestingly, we observed a large superiority of RE over COMB and AE, with a SMD 0.4 difference. This result is in accordance with previous meta-analyses in healthy adults [24–26], though the topic remains debated in the current literature [199, 200]. In addition, muscle strength gains were diminished across all treatment subgroups, highlighting the potential impact of various cancer therapies. A potential interference effect from COMB was also hypothesized in a previous exercise trial in men with prostate cancer [31]. Overall, it is reasonable to assume that women with breast cancer may not fully recover after the treatment sessions [19], especially due to the toxicities of taxanes and other agents affecting the nervous system [201, 202]. In terms of strategies to mitigate this potential interference effect, our subgroup analyses indicated that higher volumes and intensities of AE were associated with better results within COMB, though these strategies may be impractical due to patients’ deconditioning and symptoms [185]. Interestingly, our findings contrast with recent theoretical models [203] indicating that prescribing HIIT within COMB may help reduce the risk of the potential interference effect. It should be noted that HIIT protocols vary widely in intensity, duration, and rest intervals, and only a few have been tested specifically in women with breast cancer [29]. Given the unclear interaction between multiple treatment regimens and different exercise stimuli, this subject certainly requires further investigation. Nevertheless, when targeting muscle strength as a primary outcome, RE alone should be preferred to achieve better results.

### Strengths and Limitations

To our knowledge, this is the first systematic review and network meta-analysis to compare the effects of COMB, RE and AE programs in women with breast cancer and to explore the potential interference effect. In addition, this is one of the largest syntheses of exercise in women with breast cancer. Strengths of this study include: (1) the inclusion of 112 randomised trials with 8695 women with breast cancer, (2) a network meta-analysis comparing COMB, RE alone and AE alone, and (3) subgroup analyses on a range of demographic, clinical and exercise prescription characteristics. However, there are limitations that are worthy of comment. First, the majority of the studies focused on women with stage 0–II breast cancer enrolled in short-term exercise programs (< 24 weeks). As a result, our findings may not be generalisable to patients with advanced breast cancer or those engaging in long-term exercise programs (≥ 24 weeks). Second, we did not investigate the effects of different exercise modes and the potential interference effect on cardiorespiratory fitness. It is well-established that both AE and COMB have major effects on cardiorespiratory fitness, with minimal differences between them (0.14 mL.kg^−1^.min^−1^) [32]. As a result, we focused on outcomes potentially affected by the combination of RE and AE. Nevertheless, RE was the most effective intervention for the 6-min walk and 400-m walk tests, whereas COMB attenuated these benefits and AE was less effective, indicating possible interference effects in tasks commonly used to assess cardiorespiratory fitness components related to activities of daily living. Third, we defined the interference effect as a difference of SMD 0.2 or MD 0.2 kg between RE and COMB across the outcome measures given the lack of MCID previously stated for the range of outcomes investigated. Although a larger difference may be desirable, any mode of exercise promoted benefits (SMD > 0.3) in the outcomes investigated compared with controls. Therefore, a difference of SMD 0.2 or MD 0.2 kg may be sufficient to indicate the superiority of RE over COMB and can be considered a relevant threshold for identifying an interference effect, especially in women with breast cancer where small improvements can be impactful. Fourth, subgroup differences between RE and COMB were focused on effect sizes (SMD > 0.2 or MD > 0.2 kg) rather than statistical significance given the range of subgroups tested. This approach may be preferable given the number of studies available within each subgroup and the exploratory nature of subgroup testing within network meta-analysis. Finally, it remains unclear whether the attenuation observed in COMB reflects a true physiological interference or differences in RE dose, as we observed across the outcomes investigated. Although the RE dose was generally comparable within studies directly comparing RE and COMB programs, this was not consistent across the entire network. Consequently, the findings should be interpreted with caution. More trials that carefully match RE volume across interventions are required to determine whether the interference effect stems from physiological mechanisms or from exercise prescription variation.

## Conclusions

Our findings emphasise the importance of personalised exercise medicine targeted to breast cancer treatment characteristics to address key outcomes such as fatigue, lean mass, physical function and muscle strength. Although COMB may offer benefits across the outcomes investigated, the prescription of RE alone (e.g., supervised, thrice a week, 6–7 resistance exercises, two sets per exercise at 75% of 1-RM) may be preferred to better target lean mass and muscle strength and minimize the potential interference effect. In addition, our results indicated that RE provided superior outcomes for fatigue and physical function tests, particularly during adjuvant and hormonal therapies. Our study provides evidence for refining exercise medicine on the basis of patient characteristics, treatment regimen and exercise-specific goals when prescribing RE or COMB in women with breast cancer.

## Supplementary Information

Below is the link to the electronic supplementary material.

Supplementary file1 (DOCX 738 KB)

## Declarations

### Data Availability

Data used in the present study such as data extraction templates, forms and analysis will be made available upon reasonable request to the corresponding author.
