# Supplementary material for: Do Combined Resistance and Aerobic Exercise Programs Cause an Interference Effect in Women with Breast Cancer? A Systematic Review and Network Meta-analysis
Source: Sports Med. 2026 Feb 20;56(7):1675–700. doi: 10.1007/s40279-026-02402-x (PMC13388779; doi:10.1007/s40279-026-02402-x)
Supplement: Supplementary file 1 — Supplementary file1 (DOCX 738 KB) [file 40279_2026_2402_MOESM1_ESM.docx]

**Interference effect from combined resistance and aerobic exercise programs in women with breast cancer: a systematic review and network meta-analysis**

**Appendix S1. Search strategy**

***PubMed***

(“breast cancer” [title/abstract] OR Breast Neoplasm [title/abstract] OR Neoplasm, Breast [title/abstract] OR Breast Tumors [title/abstract] OR Breast Tumor [title/abstract] OR Tumor, Breast [title/abstract] OR Tumors, Breast [title/abstract] OR Neoplasms, Breast [title/abstract] OR Breast Cancer [title/abstract] OR Cancer, Breast [title/abstract] OR Mammary Cancer [title/abstract] OR Cancer, Mammary [title/abstract] OR Cancers, Mammary [title/abstract] OR Mammary Cancers [title/abstract] OR Malignant Neoplasm of Breast [title/abstract] OR Breast Malignant Neoplasm [title/abstract] OR Breast Malignant Neoplasms [title/abstract] OR Malignant Tumor of Breast [title/abstract] OR Breast Malignant Tumor [title/abstract] OR Breast Malignant Tumors [title/abstract] OR Cancer of Breast [title/abstract] OR Cancer of the Breast [title/abstract] OR Mammary Carcinoma, Human [title/abstract] OR Carcinoma, Human Mammary [title/abstract] OR Carcinomas, Human Mammary [title/abstract] OR Human Mammary Carcinomas [title/abstract] OR Mammary Carcinomas, Human [title/abstract] OR Human Mammary Carcinoma [title/abstract] OR Mammary Neoplasms, Human [title/abstract] OR Human Mammary Neoplasm [title/abstract] OR Human Mammary Neoplasms [title/abstract] OR Neoplasm, Human Mammary [title/abstract] OR Neoplasms, Human Mammary [title/abstract] OR Mammary Neoplasm, Human [title/abstract] OR Breast Carcinoma [title/abstract] OR Breast Carcinomas [title/abstract] OR Carcinoma, Breast [title/abstract] OR Carcinomas, Breast [title/abstract]) AND (exercise [title/abstract] OR Exercises [title/abstract] OR Exercise, Physical [title/abstract] OR Exercises, Physical [title/abstract] OR Physical Exercise [title/abstract] OR Physical Exercises [title/abstract] OR Exercise, Aerobic [title/abstract] OR Aerobic Exercise [title/abstract] OR Aerobic Exercises [title/abstract] OR Exercises, Aerobic [title/abstract] OR Exercise Training [title/abstract] OR Exercise Trainings [title/abstract] OR Training, Exercise [title/abstract] OR Trainings, Exercise [title/abstract] OR "resistance training" [title/abstract] OR "resistance exercise" [title/abstract] OR Training, Resistance [title/abstract] OR Strength Training [title/abstract] OR Training, Strength [title/abstract] OR Weight-Lifting [title/abstract] OR Strengthening Program [title/abstract] OR Strengthening Program, Weight-Lifting [title/abstract] OR Strengthening Programs, Weight-Lifting [title/abstract] OR Weight Lifting Strengthening Program [title/abstract] OR Weight-Lifting Strengthening Programs [title/abstract] OR Weight-Lifting Exercise Program [title/abstract] OR Exercise Program, Weight-Lifting [title/abstract] OR Exercise Programs, Weight-Lifting [title/abstract] OR Weight Lifting Exercise Program [title/abstract] OR Weight-Lifting Exercise Programs [title/abstract] OR Weight-Bearing Strengthening Program [title/abstract] OR Strengthening Program, Weight-Bearing [title/abstract] OR Strengthening Programs, Weight-Bearing [title/abstract] OR Weight Bearing Strengthening Program [title/abstract] OR Weight-Bearing Strengthening Programs [title/abstract] OR Weight-Bearing Exercise Program [title/abstract] OR Exercise Program, Weight-Bearing [title/abstract] OR Exercise Programs, Weight-Bearing [title/abstract] OR Weight Bearing Exercise Program [title/abstract] OR Weight-Bearing Exercise Programs [title/abstract] OR High Intensity Interval Training [title/abstract] OR High-Intensity Interval Trainings [title/abstract] OR Interval Training, High-Intensity [title/abstract] OR Interval Trainings, High-Intensity [title/abstract] OR Training, High-Intensity Interval [title/abstract] OR Trainings, High-Intensity Interval [title/abstract] OR High-Intensity Intermittent Exercise [title/abstract] OR Exercise, High-Intensity Intermittent [title/abstract] OR Exercises, High-Intensity Intermittent [title/abstract] OR High-Intensity Intermittent Exercises [title/abstract] OR Sprint Interval Training [title/abstract] OR Sprint Interval Trainings [title/abstract]) AND ("clinical trial" (Mesh) OR "randomized controlled trial" [title/abstract] OR "controlled clinical trial" [title/abstract] OR "randomized" [title/abstract] OR "randomly" [title/abstract] OR "groups" [title/abstract] OR "trial" [title/abstract]) NOT (Rat [title/abstract] OR Rats [title/abstract] OR Mice [title/abstract] OR Mices [title/abstract] OR Review [title/abstract] OR “Systematic Review” [title/abstract] OR Meta-analysis [title/abstract] OR “Cross-Sectional study” [title/abstract] OR Protocol [title/abstract])

***EBSCO host (CINAHL and SPORTDiscus) + Web of Science***

(“breast cancer” OR Breast Neoplasm OR Neoplasm, Breast OR Breast Tumors OR Breast Tumor OR Tumor, Breast OR Tumors, Breast OR Neoplasms, Breast OR Breast Cancer OR Cancer, Breast OR Mammary Cancer OR Cancer, Mammary OR Cancers, Mammary OR Mammary Cancers OR Malignant Neoplasm of Breast OR Breast Malignant Neoplasm OR Breast Malignant Neoplasms OR Malignant Tumor of Breast OR Breast Malignant Tumor OR Breast Malignant Tumors OR Cancer of Breast OR Cancer of the Breast OR Mammary Carcinoma, Human OR Carcinoma, Human Mammary OR Carcinomas, Human Mammary OR Human Mammary Carcinomas OR Mammary Carcinomas, Human OR Human Mammary Carcinoma OR Mammary Neoplasms, Human OR Human Mammary Neoplasm OR Human Mammary Neoplasms OR Neoplasm, Human Mammary OR Neoplasms, Human Mammary OR Mammary Neoplasm, Human OR Breast Carcinoma OR Breast Carcinomas OR Carcinoma, Breast OR Carcinomas, Breast) AND (exercise OR Exercises OR Exercise, Physical OR Exercises, Physical OR Physical Exercise OR Physical Exercises OR Exercise, Aerobic OR Aerobic Exercise OR Aerobic Exercises OR Exercises, Aerobic OR Exercise Training OR Exercise Trainings OR Training, Exercise OR Trainings, Exercise OR "resistance training" OR "resistance exercise" OR Training, Resistance OR Strength Training OR Training, Strength OR Weight-Lifting OR Strengthening Program OR Strengthening Program, Weight-Lifting OR Strengthening Programs, Weight-Lifting OR Weight Lifting Strengthening Program OR Weight-Lifting Strengthening Programs OR Weight-Lifting Exercise Program OR Exercise Program, Weight-Lifting OR Exercise Programs, Weight-Lifting OR Weight Lifting Exercise Program OR Weight-Lifting Exercise Programs OR Weight-Bearing Strengthening Program OR Strengthening Program, Weight-Bearing OR Strengthening Programs, Weight-Bearing OR Weight Bearing Strengthening Program OR Weight-Bearing Strengthening Programs OR Weight-Bearing Exercise Program OR Exercise Program, Weight-Bearing OR Exercise Programs, Weight-Bearing OR Weight Bearing Exercise Program OR Weight-Bearing Exercise Programs OR High Intensity Interval Training OR High-Intensity Interval Trainings OR Interval Training, High-Intensity OR Interval Trainings, High-Intensity OR Training, High-Intensity Interval OR Trainings, High-Intensity Interval OR High-Intensity Intermittent Exercise OR Exercise, High-Intensity Intermittent OR Exercises, High-Intensity Intermittent OR High-Intensity Intermittent Exercises OR Sprint Interval Training OR Sprint Interval Trainings) AND ("clinical trial" OR "randomized controlled trial" OR "controlled clinical trial" OR "randomized" OR "randomly" OR "groups" OR "trial") NOT (Rat OR Rats OR Mice OR Mices OR Review OR “Systematic Review” OR Meta-analysis OR “Cross-Sectional study” OR Protocol)

***Embase***

('breast cancer':ab,ti OR 'breast tumor':ab,ti OR 'breast carcinoma':ab,ti) AND (exercise:ab,ti OR 'aerobic exercise':ab,ti OR 'resistance training':ab,ti OR 'weight lifting':ab,ti OR 'weight training':ab,ti OR 'weight machine':ab,ti OR 'weight bearing':ab,ti OR 'high intensity interval training':ab,ti) AND ('clinical trial':ab,ti OR 'randomized controlled trial':ab,ti OR 'controlled study':ab,ti)

***LILACS and Scielo***

("câncer de mama" OR "cáncer de mama") AND (exercicio OR ejercicio OR "treinamento de força" OR "treinamento resistido" OR musculação OR "entrenamiento de Resistencia" OR "entrenamiento de fuerza" OR "treinamento aerobico" OR "entrenamiento aerobico" OR exercicio OR ejercicio OR entrenamiento OR "ejercicio isometrico" OR "treinamento intervalado de alta intensidade" OR "entrenamiento de intervalos de alta intensidad")

**Table S1.** Pairwise meta-analysis on fatigue.

| **Authors** | **Group 1** | **Group 2** | **Group 1** | | **Group 2** | | **Between-group** |
| --- | --- | --- | --- | --- | --- | --- | --- |
|  |  |  | **n** | **MD ± SD** | **n** | **MD ± SD** | **SMD (95% CI)** |
| Segal et al. 2001 | AE | Controls | 40 | 2.1 ± 17.1 | 41 | 6.7 ± 17.31 | -0.26 (-0.7 to 0.17) |
| Segal et al. 2001 | AE | Controls | 42 | 1.5 ± 24.96 | 41 | 6.7 ± 17.31 | -0.24 (-0.67 to 0.19) |
| Burnham et al. 2002 | AE | Controls | 12 | -16.1 ± 21.25 | 6 | 3.2 ± 30.48 | 0.75 (-1.77 to 0.27) |
| Courneya et al. 2003 | AE | Controls | 24 | -9.3 ± 10.2 | 28 | -2 ± 7.5 | 0.81 (-1.38 to -0.24) |
| Pinto et al. 2003 | AE | Controls | 12 | -1.51 ± 6.84 | 6 | 2 ± 5.62 | 0.52 (-1.51 to 0.48) |
| Campbell et al. 2005 | COMB | Controls | 10 | -2.11 ± 2.3 | 9 | -0.25 ± 2.5 | 0.74 (-1.68 to 0.2) |
| Drouin et al. 2005 | AE | Controls | 10 | -2.17 ± 7.16 | 10 | 6.33 ± 12.13 | 0.82 (-1.74 to 0.1) |
| Drouin et al. 2005 | AE | Controls | 10 | -12.8 ± 64.33 | 10 | 12.67 ± 67.61 | 0.37 (-1.26 to 0.52) |
| Mock et al. 2005 | AE | Controls | 54 | 1 ± 2.9 | 54 | 1.6 ± 2.5 | 0.22 (-0.6 to 0.16) |
| Pinto et al. 2005 | AE | Controls | 39 | -15.39 ± 22.55 | 43 | 0.62 ± 25.64 | 0.65 (-1.1 to -0.21) |
| Battaglini et al. 2006 | COMB | Controls | 10 | -1.35 ± 1.57 | 10 | 1.84 ± 1.04 | 2.3 (-3.48 to -1.12) |
| Courneya et al. 2007 | RE | Controls | 76 | 0.9 ± 10.9 | 73 | -0.7 ± 10.9 | 0.15 (-0.18 to 0.47) |
| Courneya et al. 2007 | AE | Controls | 74 | 0.4 ± 10.97 | 73 | -0.7 ± 10.9 | 0.1 (-0.22 to 0.42) |
| Courneya et al. 2007 | RE | AE | 76 | 0.9 ± 10.9 | 74 | 0.4 ± 10.97 | 0.05 (-0.27 to 0.37) |
| Mutrie et al. 2007 | COMB | Controls | 82 | 4 ± 11.11 | 92 | 3.2 ± 12.41 | 0.07 (-0.23 to 0.37) |
| Yuen et al. 2007 | RE | Controls | 7 | -2.15 ± 1.92 | 7 | -0.61 ± 1.53 | 0.83 (-1.94 to 0.28) |
| Yuen et al. 2007 | AE | Controls | 8 | -1.66 ± 1.64 | 7 | -0.61 ± 1.53 | 0.62 (-1.67 to 0.42) |
| Yuen et al. 2007 | RE | AE | 7 | -2.15 ± 1.92 | 8 | -1.66 ± 1.64 | 0.26 (-1.28 to 0.76) |
| Hwang et al. 2008 | COMB | Controls | 17 | -0.57 ± 0.45 | 20 | 0.28 ± 0.46 | 1.82 (-2.6 to -1.04) |
| Milne et al. 2008 | COMB | Controls | 29 | -3.8 ± 3.73 | 29 | 0.9 ± 4.39 | 1.14 (-1.7 to -0.58) |
| Cadmus et al. 2009 | AE | Controls | 25 | -0.1 ± 9.1 | 25 | 2.9 ± 10.1 | -0.31 (-0.87 to 0.25) |
| Cadmus et al. 2009 & Irwin et al. 2009 | AE | Controls | 37 | 1.6 ± 6.6 | 37 | 1.2 ± 7.1 | 0.06 (-0.4 to 0.51) |
| Moros et al. 2010 | COMB | Controls | 10 | 15.6 ± 21.18 | 7 | 11.1 ± 16.61 | -0.22 (-0.75 to 1.19) |
| Cantarero-Villanueva et al. 2011 | COMB | Controls | 32 | -2.34 ± 2.29 | 35 | 0.15 ± 1.42 | 1.3 (-1.83 to -0.77) |
| Winters-Stone et al. 2011 & Winters-Stone et al. 2012 | RE | Controls | 36 | 0.2 ± 4.18 | 31 | -0.3 ± 3.16 | -0.13 (-0.35 to 0.61) |
| Duijtis et al. 2012 | AE | Controls | 37 | 7.98 ± 17.16 | 89 | 2.97 ± 16.94 | 0.29 (-0.09 to 0.68) |
| Naumann et al. 2012 | COMB | Controls | 11 | -0.69 ± 1.33 | 10 | 0.26 ± 1.36 | 0.68 (-1.57 to 0.21) |
| Nikander et al. 2012 & Saarto et al. 2012 & Saarto et al. 2012 | COMB | Controls | 263 | 2.4 ± 8.69 | 237 | 2.4 ± 8.64 | 0 (-0.18 to 0.18) |
| Cormie et al. 2013 | RE | Controls | 22 | 1.6 ± 8.44 | 19 | 1 ± 7.85 | 0.07 (-0.54 to 0.69) |
| Cormie et al. 2013 | RE | Controls | 21 | 5.3 ± 7.79 | 19 | 1 ± 7.85 | 0.54 (-0.09 to 1.17) |
| Courneya et al. 2013 | AE | COMB | 95 | -11.2 ± 13.92 | 103 | -10.8 ± 13.98 | -0.03 (-0.31 to 0.25) |
| Courneya et al. 2013 | AE | COMB | 100 | -7.8 ± 13.78 | 103 | -10.8 ± 13.98 | 0.22 (-0.06 to 0.49) |
| Courneya et al. 2013 | AE | COMB | 95 | -7 ± 7.71 | 103 | -6.2 ± 7.77 | -0.1 (-0.38 to 0.18) |
| Courneya et al. 2013 | AE | COMB | 100 | -5.2 ± 7.65 | 103 | -6.2 ± 7.77 | 0.13 (-0.15 to 0.4) |
| Ergun et al. 2013 | COMB | Controls | 20 | -0.58 ± 2.13 | 20 | -0.14 ± 1.72 | 0.22 (-0.84 to 0.4) |
| Ergun et al. 2013 | AE | Controls | 20 | -0.22 ± 2.48 | 20 | -0.14 ± 1.72 | 0.04 (-0.66 to 0.58) |
| Ergun et al. 2013 | COMB | AE | 20 | -0.58 ± 2.13 | 20 | -0.22 ± 2.48 | 0.15 (-0.77 to 0.47) |
| Hayes et al. 2013 | COMB | Controls | 67 | 4.9 ± 9.61 | 60 | 4.6 ± 11.26 | 0.03 (-0.32 to 0.38) |
| Hayes et al. 2013 | COMB | Controls | 67 | 6.8 ± 12.32 | 60 | 4.6 ± 11.26 | 0.18 (-0.16 to 0.53) |
| Kulkarni et al. 2013 | AE | Controls | 30 | -0.05 ± 0.99 | 30 | 0.46 ± 0.6 | 0.62 (-1.13 to -0.1) |
| Rogers et al. 2013 | COMB | Controls | 11 | 0 ± 1.91 | 9 | 0.3 ± 1.55 | 0.16 (-1.05 to 0.72) |
| Rogers et al. 2013 | COMB | Controls | 11 | 0 ± 1.55 | 9 | -0.2 ± 1.35 | -0.13 (-0.75 to 1.01) |
| Hornsby et al. 2014 | AE | Controls | 10 | -2.5 ± 14.15 | 10 | -2.2 ± 5.21 | -0.03 (-0.9 to 0.85) |
| Husebo et al. 2014 | COMB | Controls | 29 | 1.73 ± 4.17 | 31 | 1.77 ± 4.09 | 0.01 (-0.52 to 0.5) |
| Rogers et al. 2014 | COMB | Controls | 20 | 0.4 ± 1.9 | 22 | -0.1 ± 1.9 | -0.26 (-0.35 to 0.87) |
| Rogers et al. 2014 | COMB | Controls | 20 | -0.4 ± 1.1 | 22 | 0.1 ± 1.8 | 0.33 (-0.94 to 0.28) |
| Rogers et al. 2014 | COMB | Controls | 20 | -3.8 ± 4.1 | 22 | -1.1 ± 6.4 | 0.49 (-1.1 to 0.13) |
| Steindorf et al. 2014 & Wiskemann et al. 2017 | RE | Controls | 77 | -0.5 ± 1.57 | 78 | 0 ± 1.58 | 0.32 (-0.63 to 0) |
| Steindorf et al. 2014 & Wiskemann et al. 2017 | RE | Controls | 77 | -8.1 ± 21.71 | 78 | -2.2 ± 21.85 | 0.27 (-0.59 to 0.05) |
| Al-Majid et al. 2015 | AE | Controls | 7 | 0 ± 2 | 7 | 3.8 ± 2.07 | 1.75 (-3.04 to -0.46) |
| Do et al. 2015 | COMB | Controls | 32 | -11.1 ± 14.47 | 30 | 5.1 ± 18.34 | 0.97 (-1.5 to -0.44) |
| Do et al. 2015 | COMB | Controls | 32 | -11.2 ± 12.47 | 30 | 1.8 ± 15 | 0.93 (-1.46 to -0.41) |
| Do et al. 2015 [2] | RE | Controls | 22 | -26 ± 23.45 | 22 | -23.3 ± 23.35 | 0.11 (-0.7 to 0.48) |
| Naraphong et al. 2015 | AE | Controls | 9 | -0.94 ± 1.96 | 12 | -0.38 ± 2.49 | 0.24 (-1.1 to 0.63) |
| Schmidt et al. 2015 | RE | Controls | 21 | 16.4 ± 20.07 | 26 | 12.96 ± 19.86 | -0.17 (-0.41 to 0.75) |
| Schmidt et al. 2015 | AE | Controls | 20 | 16.89 ± 24.41 | 26 | 12.96 ± 19.86 | -0.18 (-0.41 to 0.76) |
| Schmidt et al. 2015 | RE | AE | 21 | 16.4 ± 20.07 | 20 | 16.89 ± 24.41 | 0.02 (-0.63 to 0.59) |
| Schmidt et al. 2015 | RE | Controls | 21 | 1.3 ± 3.16 | 26 | 2.84 ± 3.43 | 0.46 (-1.04 to 0.13) |
| Schmidt et al. 2015 | AE | Controls | 20 | 3.59 ± 4.34 | 26 | 2.84 ± 3.43 | -0.19 (-0.39 to 0.78) |
| Schmidt et al. 2015 | RE | AE | 21 | 1.3 ± 3.16 | 20 | 3.59 ± 4.34 | 0.59 (-1.22 to 0.03) |
| Schmidt et al. 2015 [2] | RE | Controls | 49 | -1.1 ± 16.96 | 46 | 4.6 ± 17.13 | 0.33 (-0.74 to 0.07) |
| Travier et al. 2015 | COMB | Controls | 87 | 1.9 ± 4.28 | 77 | 2.3 ± 4.25 | 0.09 (-0.4 to 0.21) |
| Travier et al. 2015 | COMB | Controls | 87 | 7.4 ± 23.08 | 77 | 9 ± 22.83 | 0.07 (-0.38 to 0.24) |
| Travier et al. 2015 | COMB | Controls | 87 | -7.5 ± 16.89 | 77 | -5.2 ± 16.79 | -0.14 (-0.44 to 0.17) |
| van Waart et al. 2015 | COMB | Controls | 76 | 2.5 ± 4 | 77 | 3 ± 4.3 | 0.12 (-0.44 to 0.2) |
| van Waart et al. 2015 | COMB | Controls | 76 | 15.7 ± 21.94 | 77 | 20.1 ± 22.39 | 0.2 (-0.52 to 0.12) |
| Cornette et al. 2016 | COMB | Controls | 10 | -6.9 ± 18.04 | 9 | -4.6 ± 18.92 | 0.12 (-1.02 to 0.78) |
| de Luca et al. 2016 | COMB | Controls | 10 | 16.9 ± 15.14 | 10 | -4.8 ± 18.23 | 1.24 (0.27 to 2.22) |
| Galiano-Castillo et al. 2016 & Ariza-Garcia et al. 2019 | COMB | Controls | 36 | -1.93 ± 2.36 | 36 | -0.21 ± 2.58 | 0.69 (-1.16 to -0.21) |
| Hagstrom et al. 2016 | RE | Controls | 20 | 6.65 ± 9.05 | 19 | 1.53 ± 10.44 | 0.51 (-0.12 to 1.15) |
| Shobeiri et al. 2016 | AE | Controls | 27 | -25.93 ± 21.29 | 26 | -5.56 ± 16.57 | 1.05 (-1.63 to -0.47) |
| Waked et al. 2016 | COMB | Controls | 23 | -4.48 ± 1.49 | 23 | -0.25 ± 0.92 | 3.37 (-4.29 to -2.45) |
| Campbell et al. 2018 | AE | Controls | 10 | 4.7 ± 10.1 | 9 | 0.5 ± 11.4 | 0.37 (-0.54 to 1.28) |
| de Paulo et al. 2018 & de Paulo et al. 2019 | COMB | Controls | 18 | 12.2 ± 12.57 | 18 | -10.6 ± 13.75 | 1.69 (0.92 to 2.47) |
| de Paulo et al. 2018 & de Paulo et al. 2019 | COMB | Controls | 18 | -15.7 ± 18.1 | 18 | -3 ± 20.23 | 0.65 (-1.32 to 0.03) |
| Dieli-Conwright et al. 2018 & Dieli-Conwright et al. 2018 [2] | COMB | Controls | 46 | 7.4 ± 9.4 | 45 | -0.5 ± 9.45 | 0.83 (0.4 to 1.26) |
| Dieli-Conwright et al. 2018 & Dieli-Conwright et al. 2018 [2] | COMB | Controls | 46 | -4.2 ± 1.8 | 45 | 0.5 ± 2.26 | 2.28 (-2.81 to -1.75) |
| Mijwel et al. 2018 & Mijwel et al. 2018 | COMB | Controls | 74 | 0.07 ± 3.05 | 60 | 1.64 ± 2.88 | 0.52 (-0.87 to -0.18) |
| Mijwel et al. 2018 & Mijwel et al. 2018 | AE | Controls | 70 | 1.06 ± 2.62 | 60 | 1.64 ± 2.88 | 0.21 (-0.56 to 0.14) |
| Mijwel et al. 2018 & Mijwel et al. 2018 | COMB | AE | 74 | 0.07 ± 3.05 | 70 | 1.06 ± 2.62 | 0.35 (-0.67 to -0.02) |
| Mijwel et al. 2018 & Mijwel et al. 2018 | COMB | Controls | 74 | -2.56 ± 27.32 | 60 | 14.41 ± 25.13 | 0.64 (-0.99 to -0.29) |
| Mijwel et al. 2018 & Mijwel et al. 2018 | AE | Controls | 70 | 3.05 ± 23.4 | 60 | 14.41 ± 25.13 | 0.47 (-0.82 to -0.12) |
| Mijwel et al. 2018 & Mijwel et al. 2018 | COMB | AE | 74 | -2.56 ± 27.32 | 70 | 3.05 ± 23.4 | 0.22 (-0.55 to 0.11) |
| Reis et al. 2018 | COMB | Controls | 14 | -1 ± 2.7 | 14 | -0.15 ± 3.25 | 0.28 (-1.02 to 0.47) |
| Scott et al. 2018 | AE | Controls | 33 | 2 ± 11.53 | 32 | 0 ± 10.15 | 0.18 (-0.31 to 0.67) |
| Ammitzboll et al. 2019 | RE | Controls | 68 | -0.43 ± 1.99 | 52 | -3.32 ± 1.96 | 1.45 (1.05 to 1.86) |
| Baglia et al. 2019 | COMB | Controls | 45 | 5.7 ± 6.67 | 38 | 0.5 ± 6.6 | 0.78 (0.33 to 1.22) |
| Baglia et al. 2019 | COMB | Controls | 45 | 6 ± 5.99 | 38 | 0.9 ± 5.98 | 0.84 (0.39 to 1.3) |
| Ceseiko et al. 2019 & Ceseiko et al. 2020 | RE | Controls | 27 | -7.9 ± 18.43 | 28 | 7.3 ± 15.79 | 0.87 (-1.43 to -0.32) |
| Dong et al. 2019 & Dong et al. 2020 | COMB | Controls | 26 | 5.2 ± 10.11 | 24 | -0.63 ± 9.66 | 0.58 (0.01 to 1.15) |
| dos Santos et al. 2019 & Moraes et al. 2021 | RE | Controls | 13 | -2.8 ± 2.34 | 13 | -0.9 ± 2.23 | 0.81 (-1.61 to 0) |
| dos Santos et al. 2019 & Moraes et al. 2021 | RE | Controls | 13 | 9.3 ± 22.29 | 13 | 0.4 ± 21.29 | 0.4 (-0.38 to 1.17) |
| Yee et al. 2019 | COMB | Controls | 8 | 5.6 ± 3.2 | 6 | -1.8 ± 3.9 | 1.97 (0.61 to 3.33) |
| Yee et al. 2019 | COMB | Controls | 8 | -6.9 ± 13.2 | 6 | 0 ± 7 | 0.58 (-1.67 to 0.5) |
| Kim et al. 2020 | COMB | Controls | 23 | 1.34 ± 1.5 | 25 | 0.48 ± 1.5 | -0.56 (-0.01 to 1.14) |
| Pereira-Rodriguez et al. 2020 | COMB | Controls | 80 | -10.6 ± 8.25 | 66 | 0.3 ± 5.11 | -1.55 (-1.92 to -1.18) |
| Pereira-Rodriguez et al. 2020 | COMB | Controls | 70 | -15.3 ± 4.92 | 66 | 0.3 ± 5.11 | -3.1 (-3.6 to -2.59) |
| Santagnello et al. 2020 | RE | Controls | 11 | -1.34 ± 0.4 | 9 | 0.22 ± 0.5 | 3.34 (-4.79 to -1.89) |
| Scott et al. 2020 | AE | Controls | 58 | 2.8 ± 12.05 | 57 | 0.3 ± 10.8 | 0.22 (-0.15 to 0.58) |
| Scott et al. 2020 | AE | Controls | 59 | 2 ± 8.95 | 57 | 0.3 ± 10.8 | 0.17 (-0.19 to 0.54) |
| Aydin et al. 2021 | AE | Controls | 24 | -24.6 ± 24.46 | 24 | -19.9 ± 25.7 | 0.18 (-0.75 to 0.38) |
| Brown et al. 2021 & Brown et al. 2021 [2] | COMB | Controls | 87 | 3.24 ± 1.81 | 90 | 4.38 ± 1.79 | -0.63 (-0.93 to -0.33) |
| Gal et al. 2021 | COMB | Controls | 109 | -0.9 ± 3.2 | 113 | -0.3 ± 3.8 | 0.17 (-0.43 to 0.09) |
| Lee et al. 2021 | AE | Controls | 15 | 11.4 ± 12.66 | 15 | 5.3 ± 11.46 | -0.49 (-0.24 to 1.22) |
| Mostafaei et al. 2021 | COMB | Controls | 30 | -5.93 ± 9.42 | 30 | 1.16 ± 8.93 | 0.76 (-1.29 to -0.24) |
| Bringel et al. 2022 | COMB | Controls | 13 | -2.44 ± 2.85 | 13 | -0.26 ± 3.76 | 0.63 (-1.42 to 0.16) |
| Bringel et al. 2022 | COMB | Controls | 13 | -1.87 ± 3.44 | 13 | -0.55 ± 3.98 | 0.34 (-1.12 to 0.43) |
| Bringel et al. 2022 | COMB | Controls | 13 | -0.44 ± 2.95 | 13 | -0.41 ± 3.3 | 0.01 (-0.78 to 0.76) |
| Knoerl et al. 2022 | COMB | Controls | 26 | -3 ± 18.51 | 21 | 1.1 ± 18.92 | 0.22 (-0.79 to 0.36) |
| Sturgeon et al. 2022 | AE | Controls | 8 | -5.6 ± 21.31 | 7 | 15 ± 18.3 | 0.97 (-2.06 to 0.12) |
| Wonders et al. 2022 | COMB | Controls | 123 | 7.5 ± 34.64 | 120 | -0.7 ± 34.52 | 0.24 (-0.02 to 0.49) |
| Wonders et al. 2022 | COMB | Controls | 123 | -4.3 ± 14.55 | 120 | 0.5 ± 15.91 | 0.31 (-0.57 to -0.06) |
| Adams-Campbell et al. 2023 | AE | Controls | 15 | 1.3 ± 5.9 | 15 | -1.9 ± 12.4 | 0.32 (-0.4 to 1.04) |
| Antunes et al. 2023 | COMB | Controls | 44 | 6.09 ± 27.14 | 41 | 26.06 ± 27.03 | 0.73 (-1.17 to -0.29) |
| Darvishi et al. 2023 | COMB | Controls | 32 | -2.61 ± 1.81 | 32 | 0.75 ± 1.79 | 1.84 (-2.43 to -1.25) |
| Han et al. 2023 | COMB | Controls | 23 | -1.28 ± 1.82 | 20 | -1.27 ± 1.69 | 0.01 (-0.6 to 0.59) |
| Soriano-Maldonado et al. 2023 | RE | Controls | 32 | 1.8 ± 5.66 | 28 | 1.4 ± 5.82 | 0.07 (-0.44 to 0.58) |
| Mavropalias et al. 2023 | COMB | Controls | 40 | 7.51 ± 8.41 | 43 | 3.83 ± 7.75 | 0.45 (0.02 to 0.89) |
| Casanovas-Álvarez et al. 2024 | COMB | CONT | 32 | -11.1 ± 20.27 | 32 | -5 ± 26.21 | 0.26 (-0.23 to 0.75) |
| Garcia-Roca et al. 2024 & Suárez-Alcázar et al. 2024 | COMB | CONT | 21 | -1.95 ± 12.55 | 28 | -1.78 ± 19.22 | 0.01 (-0.56 to 0.58) |
| Garcia-Roca et al. 2024 & Suárez-Alcázar et al. 2024 | COMB | CONT | 31 | -9.2 ± 17.95 | 28 | -1.78 ± 19.22 | 0.39 (-0.12 to 0.91) |

95% CI, 95% confidence interval; AE, aerobic exercise; COMB, combined resistance and aerobic exercise; MD, within-group mean difference; RE, resistance exercise; SMD, standardised mean difference.

**Table S2.** Pairwise meta-analysis on lean mass.

| **Authors** | **Group 1** | **Group 2** | **Group 1** | | **Group 2** | | **Between-group** |
| --- | --- | --- | --- | --- | --- | --- | --- |
|  |  |  | **n** | **MD ± SD** | **n** | **MD ± SD** | **SMD (95% CI)** |
| Schmitz et al. 2005 & Ahmed et al. 2006 | RE | Controls | 40 | 0.88 ± 0.23 | 41 | 0.02 ± 0.23 | 0.86 (0.76 to 0.96) |
| Herrero et al. 2006 | COMB | Controls | 8 | 0.7 ± 2.56 | 8 | -0.3 ± 2.72 | 1 (-1.59 to 3.59) |
| Courneya et al. 2007 | RE | Controls | 66 | 1 ± 2.07 | 69 | 0.2 ± 1.91 | 0.8 (0.13 to 1.47) |
| Courneya et al. 2007 | AE | Controls | 64 | 0.5 ± 1.84 | 69 | 0.2 ± 1.91 | 0.3 (-0.34 to 0.94) |
| Courneya et al. 2007 | RE | AE | 66 | 1 ± 2.07 | 64 | 0.5 ± 1.84 | 0.5 (-0.17 to 1.17) |
| Matthews et al. 2007 | AE | Controls | 22 | 0.21 ± 1.44 | 14 | -0.31 ± 1.1 | 0.52 (-0.31 to 1.35) |
| Cadmus et al. 2009 & Irwin et al. 2009 | AE | Controls | 36 | 0.34 ± 6.61 | 33 | -0.35 ± 7.19 | 0.69 (-2.58 to 3.96) |
| Schmitz et al. 2009 | RE | Controls | 65 | -1.2 ± 7.51 | 64 | -0.7 ± 7.4 | -0.5 (-3.07 to 2.07) |
| Schmitz et al. 2010 | RE | Controls | 65 | -0.59 ± 7.24 | 68 | -1 ± 7.54 | 0.41 (-2.1 to 2.92) |
| DeNysschen et al. 2011 | AE | Controls | 36 | 0.3 ± 4.41 | 34 | -0.6 ± 6.1 | 0.9 (-1.61 to 3.41) |
| Winters-Stone et al. 2011 & Winters-Stone et al. 2012 | RE | Controls | 36 | 0.6 ± 6.6 | 31 | 0.5 ± 5.9 | 0.1 (-2.89 to 3.09) |
| Nikander et al. 2012 & Saarto et al. 2012 & Saarto et al. 2012 | COMB | Controls | 124 | 0.35 ± 1.53 | 105 | 0.27 ± 1.62 | 0.08 (-0.33 to 0.49) |
| Nikander et al. 2012 & Saarto et al. 2012 & Saarto et al. 2012 | COMB | Controls | 138 | 0.34 ± 2.09 | 131 | -0.01 ± 1.62 | 0.36 (-0.09 to 0.8) |
| Courneya et al. 2013 | AE | COMB | 95 | 0.9 ± 1.74 | 103 | 1 ± 1.81 | -0.1 (-0.59 to 0.39) |
| Courneya et al. 2013 | AE | COMB | 100 | 0.7 ± 1.79 | 103 | 1 ± 1.81 | -0.3 (-0.79 to 0.19) |
| Fernandez-Lao 2013 | COMB | Controls | 33 | 0.35 ± 3.21 | 34 | -0.4 ± 2.87 | 0.75 (-0.71 to 2.21) |
| Bruno et al. 2016 | AE | Controls | 18 | 0.7 ± 6.18 | 20 | 0.3 ± 4.1 | 0.4 (-2.97 to 3.77) |
| Buchan et al. 2016 | RE | AE | 19 | 0.3 ± 1 | 20 | 0.6 ± 0.91 | -0.3 (-0.9 to 0.3) |
| Galiano-Castillo et al. 2016 & Ariza-Garcia et al. 2019 | COMB | Controls | 19 | 0.09 ± 1.05 | 20 | 0.17 ± 1.05 | -0.08 (-0.74 to 0.58) |
| Thomas et al. 2017 | COMB | Controls | 60 | 0.32 ± 2.96 | 61 | -0.88 ± 1.99 | 1.2 (0.3 to 2.1) |
| de Paulo et al. 2018 & de Paulo et al. 2019 | COMB | Controls | 18 | 0.4 ± 3.68 | 18 | -0.3 ± 4.83 | 0.7 (-2.1 to 3.5) |
| Dieli-Conwright et al. 2018 & Dieli-Conwright et al. 2018 [2] | COMB | Controls | 46 | 2.9 ± 7.95 | 45 | -4 ± 8.16 | 6.9 (3.59 to 10.21) |
| Dieli-Conwright et al. 2018 [3] | COMB | Controls | 11 | 1.8 ± 2.3 | 10 | -0.71 ± 2.3 | 2.51 (0.54 to 4.48) |
| Uludag et al. 2018 | RE | AE | 10 | 1.1 ± 3.44 | 11 | 0.1 ± 2.72 | 1 (-1.67 to 3.67) |
| dos Santos et al. 2019 & Moraes et al. 2021 | RE | Controls | 12 | 0.5 ± 1.02 | 13 | -0.11 ± 1.69 | 0.61 (-0.47 to 1.69) |
| McNeil et al. 2019 | AE | Controls | 15 | -0.1 ± 1.38 | 13 | 0.4 ± 1.56 | -0.5 (-1.6 to 0.6) |
| McNeil et al. 2019 | AE | Controls | 15 | 0.7 ± 1.48 | 13 | 0.4 ± 1.56 | 0.3 (-0.83 to 1.43) |
| Santagnello et al. 2020 | RE | Controls | 11 | 1.02 ± 0.19 | 9 | 0.12 ± 0.23 | 0.9 (0.71 to 1.09) |
| Brown et al. 2021 & Brown et al. 2021 [2] | COMB | Controls | 87 | 0.36 ± 0.25 | 90 | -0.06 ± 0.25 | 0.42 (0.35 to 0.49) |
| Samhan et al. 2021 | AE | Controls | 30 | 3.1 ± 6.61 | 30 | 0.18 ± 2.07 | 2.92 (0.44 to 5.4) |
| Lee et al. 2022 | RE | Controls | 15 | 0.8 ± 2.55 | 15 | -0.3 ± 2.33 | 1.1 (-0.65 to 2.85) |
| Isanejad et al. 2023 | AE | Controls | 10 | 0.12 ± 1.43 | 10 | -0.2 ± 1.89 | 0.32 (-1.15 to 1.79) |
| Isanejad et al. 2023 | AE | Controls | 10 | -0.26 ± 1.78 | 10 | -0.2 ± 1.89 | -0.06 (-1.67 to 1.55) |
| Vikmoen et al. 2024 | RET | CONT | 21 | -4.9 ± 4 | 13 | -5.2 ± 4.9 | 0.07 (-0.62 to 0.76) |
| Bettariga et al. 2025 | RET | AET | 14 | 0.6 ± 0.95 | 14 | 0.4 ± 1.53 | 0.15 (-0.59 to 0.89) |

95% CI, 95% confidence interval; AE, aerobic exercise; COMB, combined resistance and aerobic exercise; MD, within-group mean difference; RE, resistance exercise; SMD, standardised mean difference.

**Table S3.** Pairwise meta-analysis on physical function.

| **Authors** | **Method** | **Group 1** | **Group 2** | **Group 1** | | **Group 2** | | **Between-group** |
| --- | --- | --- | --- | --- | --- | --- | --- | --- |
|  |  |  |  | **n** | **MD ± SD** | **n** | **MD ± SD** | **SMD (95% CI)** |
| Nieman et al. 1995 | 6-min walking test (m) | COMB | Controls | 6 | 60.8 ± 20.58 | 6 | 12 ± 43.6 | 1.32 (0.02 to 2.62) |
| Campbell et al. 2005 | 12-min walking test (m) | COMB | Controls | 10 | 328 ± 145 | 9 | -5 ± 139 | 2.24 (1.04 to 3.44) |
| Herrero et al. 2006 | 5-times sit-to-stand (sec) | COMB | Controls | 8 | -0.71 ± 0.74 | 8 | -0.04 ± 0.52 | 0.99 (-2.05 to 0.06) |
| Mutrie et al. 2007 | 12-min walking test (m) | COMB | Controls | 82 | 138 ± 186.54 | 92 | 9 ± 228.32 | 0.61 (0.31 to 0.92) |
| Schwartz et al. 2007 | 12-min walking test (m) | RE | Controls | 21 | 35 ± 309.17 | 23 | -91 ± 249.39 | 0.44 (-0.16 to 1.04) |
| Schwartz et al. 2007 | 12-min walking test (m) | AE | Controls | 22 | 244.4 ± 306.83 | 23 | -91 ± 249.39 | 1.18 (0.54 to 1.82) |
| Schwartz et al. 2007 | 12-min walking test (m) | RE | AE | 21 | 35 ± 309.17 | 22 | 244.4 ± 306.83 | -0.67 (-1.28 to -0.05) |
| Yuen et al. 2007 | 6-min walking test (m) | RE | Controls | 7 | 36.15 ± 79.31 | 7 | 19.5 ± 51.91 | 0.23 (-0.82 to 1.28) |
| Yuen et al. 2007 | 6-min walking test (m) | AE | Controls | 8 | 11.58 ± 111.38 | 7 | 19.5 ± 51.91 | -0.08 (-1.1 to 0.93) |
| Yuen et al. 2007 | 6-min walking test (m) | RE | AE | 7 | 36.15 ± 79.31 | 8 | 11.58 ± 111.38 | 0.24 (-0.78 to 1.26) |
| Portela et al. 2008 | 12-min walking test (feet) | COMB | Controls | 12 | 267.7 ± 726.63 | 9 | -54.6 ± 700.97 | 0.43 (-0.44 to 1.31) |
| Portela et al. 2008 | 12-min walking test (feet) | COMB | Controls | 13 | 380.5 ± 464.41 | 9 | -54.6 ± 700.97 | 0.73 (-0.15 to 1.62) |
| Winters-Stone et al. 2011 & Winters-Stone et al. 2012 | 5-times sit-to-stand (sec) | RE | Controls | 36 | -1.5 ± 2.29 | 31 | -1.2 ± 3.24 | 0.11 (-0.59 to 0.37) |
| Anderson et al. 2012 | 6-min walking test (m) | COMB | Controls | 43 | 54 ± 95.94 | 35 | 20.9 ± 86.81 | 0.36 (-0.09 to 0.81) |
| Milecki et al. 2013 | 6-min walking test (m) | AE | Controls | 35 | 23.95 ± 36.1 | 31 | -9.4 ± 32.6 | 0.96 (0.44 to 1.47) |
| Husebo et al. 2014 | 6-min walking test (m) | COMB | Controls | 29 | -12.87 ± 63.3 | 31 | -10.31 ± 59 | -0.04 (-0.55 to 0.47) |
| Murtezani et al. 2014 | 12-min walking test (m) | AE | Controls | 30 | 75.5 ± 84 | 32 | 9.1 ± 87.81 | 0.76 (0.25 to 1.28) |
| van Waart et al. 2015 | 30-sec chair rise (reps) | COMB | Controls | 76 | -0.2 ± 5.27 | 77 | -0.8 ± 4.88 | 0.12 (-0.2 to 0.43) |
| Buchan et al. 2016 | 6-min walking test (m) | RE | AE | 19 | 14.4 ± 32.02 | 20 | 14.7 ± 52.14 | -0.01 (-0.63 to 0.62) |
| Cornette et al. 2016 | 6-min walking test (m) | COMB | Controls | 22 | 21.7 ± 49.91 | 22 | -9.6 ± 65.75 | 0.53 (-0.08 to 1.13) |
| Galiano-Castillo et al. 2016 & Ariza-Garcia et al. 2019 | 5-times sit-to-stand (sec) | COMB | Controls | 36 | -4.28 ± 8.23 | 36 | 1.27 ± 8.46 | 0.66 (-1.13 to -0.18) |
| Galiano-Castillo et al. 2016 & Ariza-Garcia et al. 2019 | 6-min walking test (m) | COMB | Controls | 19 | 62.07 ± 130.09 | 20 | -26.34 ± 82.21 | 0.8 (0.15 to 1.46) |
| Galiano-Castillo et al. 2016 & Ariza-Garcia et al. 2019 | 10-times sit-to-stand (sec) | COMB | Controls | 19 | -2.82 ± 3.75 | 20 | 1.26 ± 2.84 | 1.21 (-1.89 to -0.52) |
| Scott et al. 2018 | 6-min walking test (m) | AE | Controls | 33 | 29 ± 93.15 | 32 | 29 ± 100.59 | 0 (-0.49 to 0.49) |
| Scott et al. 2018 | 30-sec chair rise (reps) | AE | Controls | 33 | 1 ± 3.61 | 32 | 2 ± 4 | -0.26 (-0.75 to 0.23) |
| Scott et al. 2018 | Timed-up and go (sec) | AE | Controls | 33 | -1 ± 2 | 32 | -1 ± 2 | 0 (-0.49 to 0.49) |
| Ceseiko et al. 2019 & Ceseiko et al. 2020 | 6-min walking test (m) | RE | Controls | 27 | 46.45 ± 55.86 | 28 | -23.46 ± 68.56 | 1.1 (0.53 to 1.67) |
| Ceseiko et al. 2019 & Ceseiko et al. 2020 | 30-sec chair rise (reps) | RE | Controls | 27 | 4.38 ± 4.39 | 28 | -2.22 ± 4.21 | 1.51 (0.91 to 2.12) |
| Dong et al. 2019 & 2020 | 30-sec chair rise (reps) | COMB | Controls | 26 | 4.5 ± 3.82 | 24 | 0.5 ± 3.56 | 1.07 (0.47 to 1.66) |
| Hiraoui et al. 2019 & Hiraoui et al. 2023 | 6-min walking test (m) | COMB | Controls | 20 | 90.31 ± 56.22 | 12 | -21.57 ± 47.12 | 2.06 (1.16 to 2.95) |
| Souza Filho et al. 2019 | 30-sec chair rise (reps) | RE | Controls | 38 | 2.52 ± 2.99 | 37 | -1.65 ± 2.73 | 1.44 (0.93 to 1.95) |
| Yee et al. 2019 | 6-min walking test (m) | COMB | Controls | 8 | 40 ± 23 | 6 | -46 ± 56 | 2 (0.64 to 3.37) |
| Hojan et al. 2020 | 6-min walking test (m) | COMB | Controls | 26 | 0.9 ± 52.89 | 21 | -25.6 ± 28.89 | 0.59 (0.01 to 1.18) |
| Nouri et al. 2020 | Timed-up and go (sec) | COMB | Controls | 19 | -1.3 ± 1.14 | 21 | -0.8 ± 0.98 | 0.46 (-1.09 to 0.17) |
| Nouri et al. 2020 | Timed-up and go (sec) | RE | Controls | 17 | -1.2 ± 0.87 | 21 | -0.8 ± 0.98 | 0.42 (-1.07 to 0.23) |
| Nouri et al. 2020 | Timed-up and go (sec) | COMB | RET | 19 | -1.3 ± 1.14 | 17 | -1.2 ± 0.87 | 0.1 (-0.75 to 0.56) |
| Pereira-Rodriguez et al. 2020 | 6-min walking test (m) | COMB | Controls | 80 | 53 ± 36.29 | 66 | 1 ± 16.17 | 1.78 (1.4 to 2.17) |
| Pereira-Rodriguez et al. 2020 | 6-min walking test (m) | COMB | Controls | 70 | 98 ± 12.05 | 66 | 1 ± 16.17 | 6.79 (5.91 to 7.68) |
| Santagnello et al. 2020 | 5-times sit-to-stand (sec) | RE | Controls | 11 | -1.8 ± 0.28 | 9 | 0.16 ± 0.31 | 6.39 (-8.76 to -4.02) |
| Santagnello et al. 2020 | 30-sec chair rise (reps) | RE | Controls | 11 | 3.5 ± 0.64 | 9 | 0.52 ± 0.71 | 4.25 (2.54 to 5.95) |
| Santagnello et al. 2020 | 6-min walking test (m) | RE | Controls | 11 | 40.8 ± 7.1 | 9 | -15.1 ± 7.9 | 7.17 (4.55 to 9.79) |
| Santagnello et al. 2020 | 400-m walking test (sec) | RE | Controls | 11 | -15.6 ± 4.2 | 9 | 9.35 ± 4.6 | 5.45 (-7.52 to -3.38) |
| Santagnello et al. 2020 | Timed-up and go (sec) | RE | Controls | 11 | -1.1 ± 1.2 | 9 | 0.13 ± 0.19 | 1.3 (-2.29 to -0.32) |
| Lee et al. 2021 | Timed-up and go (sec) | AE | Controls | 15 | 0.06 ± 1.31 | 15 | 0.25 ± 1.89 | 0.11 (-0.83 to 0.6) |
| Lee et al. 2021 | 30-sec chair rise (reps) | AE | Controls | 15 | 0.14 ± 2.99 | 15 | -0.37 ± 1.87 | 0.2 (-0.52 to 0.92) |
| Lee et al. 2021 | 6-min walking test (m) | AE | Controls | 15 | 51 ± 64.62 | 15 | -6.59 ± 87.3 | 0.73 (-0.01 to 1.47) |
| Ortiz et al. 2021 | 6-min walking test (m) | COMB | Controls | 61 | 23.76 ± 102.6 | 33 | 6.3 ± 86.98 | 0.18 (-0.25 to 0.6) |
| Ortiz et al. 2021 | 30-sec chair rise (reps) | COMB | Controls | 61 | 0.8 ± 3.41 | 33 | 0.17 ± 3 | 0.19 (-0.23 to 0.62) |
| Charati et al. 2022 | 6-min walking test (m) | AE | Controls | 35 | 7.43 ± 43.04 | 35 | -29.72 ± 69.02 | 0.64 (0.16 to 1.12) |
| Lee et al. 2022 | 30-sec chair rise (reps) | RE | Controls | 15 | 2.3 ± 5.3 | 15 | 0.1 ± 4.31 | 0.44 (-0.28 to 1.17) |
| Owusu et al. 2022 | 6-min walking test (m) | COMB | Controls | 91 | 34.5 ± 64.49 | 87 | 12.9 ± 64.24 | 0.33 (0.04 to 0.63) |
| Winters-Stone et al. 2022 | 5-times sit-to-stand (sec) | RET | Controls | 32 | -2.3 ± 2.6 | 29 | -2.1 ± 2.75 | 0.07 (-0.58 to 0.43) |
| Winters-Stone et al. 2022 | 5-times sit-to-stand (sec) | AE | Controls | 27 | -1.2 ± 2.78 | 29 | -2.1 ± 2.75 | -0.32 (-0.21 to 0.85) |
| Winters-Stone et al. 2022 | 5-times sit-to-stand (sec) | RE | AE | 32 | -2.3 ± 2.6 | 27 | -1.2 ± 2.78 | 0.4 (-0.92 to 0.11) |
| Winters-Stone et al. 2022 | 6-min walking test (m) | RE | Controls | 32 | 57.3 ± 72.73 | 29 | 20.4 ± 71.71 | 0.5 (-0.01 to 1.02) |
| Winters-Stone et al. 2022 | 6-min walking test (m) | AE | Controls | 27 | 31.1 ± 71.84 | 29 | 20.4 ± 71.71 | 0.15 (-0.38 to 0.67) |
| Winters-Stone et al. 2022 | 6-min walking test (m) | RE | AE | 32 | 57.3 ± 72.73 | 27 | 31.1 ± 71.84 | 0.36 (-0.16 to 0.87) |
| Antunes et al. 2023 | 30-sec chair rise (reps) | COMB | Controls | 40 | 2.5 ± 3.94 | 38 | -1.13 ± 3.59 | 0.95 (0.48 to 1.42) |
| Isanejad et al. 2023 | 30-sec chair rise (reps) | AE | Controls | 10 | 4.1 ± 5.45 | 10 | 0.3 ± 4.58 | 0.72 (-0.19 to 1.63) |
| Isanejad et al. 2023 | 30-sec chair rise (reps) | AE | Controls | 10 | 4 ± 5.88 | 10 | 0.3 ± 4.58 | 0.67 (-0.23 to 1.58) |
| Garcia-Roca et al. 2024 & Suárez-Alcázar et al. 2024 | 30-sec chair rise (reps) | COMB | CONT | 21 | 15 ± 6.56 | 28 | 4 ± 5 | 1.89 (1.21 to 2.58) |
| Garcia-Roca et al. 2024 & Suárez-Alcázar et al. 2024 | 6-min walking test (m) | COMB | CONT | 31 | 177.5 ± 171.82 | 28 | 6.8 ± 81.82 | 1.23 (0.67 to 1.79) |

95% CI, 95% confidence interval; AE, aerobic exercise; COMB, combined resistance and aerobic exercise; MD, within-group mean difference; RE, resistance exercise; SMD, standardised mean difference.

**Table S4.** Pairwise meta-analysis on muscle strength.

| **Authors** | **Method** | **Group 1** | **Group 2** | **Group 1** | | **Group 2** | | **Between-group** |
| --- | --- | --- | --- | --- | --- | --- | --- | --- |
|  |  |  |  | **n** | **MD ± SD** | **n** | **MD ± SD** | **SMD (95% CI)** |
| Nieman et al. 1995 | Leg extension (N) | COMB | Controls | 6 | 28.9 ± 33.56 | 6 | 3.5 ± 55.11 | 0.51 (-0.64 to 1.67) |
| Schmitz et al. & Ahmed et al. 2005 & 2006 | Leg Press 1-RM (Pounds) | RE | Controls | 23 | 81.8 ± 48.92 | 23 | 20.3 ± 47 | 1.26 (0.62 to 1.9) |
| Courneya et al. 2007 | Leg Extension 1-RM (kg) | RE | Controls | 77 | 8.2 ± 8.28 | 73 | 1.4 ± 7.63 | 0.85 (0.51 to 1.18) |
| Courneya et al. 2007 | Leg Extension 1-RM (kg) | AE | Controls | 71 | 3.3 ± 8.38 | 73 | 1.4 ± 7.63 | 0.24 (-0.09 to 0.56) |
| Courneya et al. 2007 | Leg Extension 1-RM (kg) | RE | AE | 77 | 8.2 ± 8.28 | 71 | 3.3 ± 8.38 | 0.59 (0.26 to 0.91) |
| Nikander et al. 2007 | Isometric knee extension (N) | COMB | Controls | 14 | 59 ± 177 | 14 | 10 ± 279.61 | 0.2 (-0.54 to 0.95) |
| Schwartz et al. 2007 | Knee Extension 1-RM (kg) | RE | Controls | 21 | 14.9 ± 33.23 | 23 | 4.6 ± 27.9 | 0.33 (-0.26 to 0.93) |
| Schwartz et al. 2007 | Knee Extension 1-RM (kg) | AE | Controls | 22 | 14.6 ± 28.52 | 23 | 4.6 ± 27.9 | 0.35 (-0.24 to 0.94) |
| Schwartz et al. 2007 | Knee Extension 1-RM (kg) | RE | AE | 21 | 14.9 ± 33.23 | 22 | 14.6 ± 28.52 | 0.01 (-0.59 to 0.61) |
| Milne et al. 2008 | Leg Press 1-RM (kg) | COMB | Controls | 29 | 23.8 ± 9.87 | 29 | 21 ± 9.85 | 0.28 (-0.24 to 0.8) |
| Schmitz et al. 2009 | Leg Press 1-RM (Pounds) | RE | Controls | 59 | 50 ± 65.02 | 63 | 3 ± 57.51 | 0.76 (0.39 to 1.13) |
| Schmitz et al. 2010 | Leg Press 1-RM (Pounds) | RE | Controls | 61 | 43 ± 49.03 | 63 | 11 ± 53.51 | 0.62 (0.26 to 0.98) |
| Winters-Stone et al. & Winters-Stone et al. 2011 & 2012 | Leg Press 1-RM (Pounds) | RE | Controls | 36 | 33.4 ± 50.85 | 31 | 17 ± 49.91 | 0.32 (-0.16 to 0.81) |
| Naumann et al. 2012 | Leg Press 1-RM (kg) | COMB | Controls | 11 | 7.7 ± 12.6 | 10 | 3.5 ± 13.91 | 0.3 (-0.56 to 1.17) |
| Nikander et al. & Saarto et al. & Saarto et al. 2012 & 2012 & 2012 | Isometric knee extension (N) | COMB | Controls | 30 | 0 ± 23 | 37 | 2 ± 27.22 | -0.08 (-0.56 to 0.4) |
| Cormie et al. 2013 | Leg Press 1-RM (kg) | RE | Controls | 22 | 38.4 ± 25.8 | 19 | 6.7 ± 25.72 | 1.21 (0.53 to 1.88) |
| Cormie et al. 2013 | Leg Press 1-RM (kg) | RE | Controls | 21 | 36.7 ± 25.66 | 19 | 6.7 ± 25.72 | 1.14 (0.47 to 1.82) |
| Courneya et al. 2013 | Leg Press 1-RM (kg) | AE | COMB | 95 | 2.5 ± 16.16 | 103 | 8.6 ± 16.83 | -0.37 (-0.65 to -0.09) |
| Courneya et al. 2013 | Leg Press 1-RM (kg) | AE | COMB | 100 | 2.5 ± 16.33 | 103 | 8.6 ± 16.83 | -0.37 (-0.64 to -0.09) |
| Steindorf et al. & Wiskemann et al. 2014 & 2017 | Isokinetic knee extension at 60 degrees (N.kg-1) | RE | Controls | 72 | 0.04 ± 0.13 | 71 | 0.02 ± 0.15 | 0.14 (-0.19 to 0.47) |
| Steindorf et al. & Wiskemann et al. 2014 & 2017 | Isometric knee extension (N.m) | RE | Controls | 72 | 0.13 ± 0.28 | 70 | 0.05 ± 0.26 | 0.3 (-0.04 to 0.63) |
| Do et al. 2015 | Isometric knee extension (kg.m.s-2) - Right | COMB | Controls | 32 | 0.9 ± 0.6 | 30 | 0.9 ± 0.5 | 0 (-0.5 to 0.5) |
| Do et al. 2015 | Isometric knee extension (kg.m.s-2) - Left | COMB | Controls | 32 | 0.9 ± 0.8 | 30 | 1 ± 0.7 | -0.13 (-0.63 to 0.37) |
| Schmidt et al. 2015 | Leg Press 1-RM (N.m) | RE | Controls | 21 | 4.44 ± 31.28 | 26 | 0.32 ± 25.56 | 0.14 (-0.43 to 0.72) |
| Schmidt et al. 2015 | Leg Press 1-RM (N.m) | AE | Controls | 20 | 2.3 ± 20.81 | 26 | 0.32 ± 25.56 | 0.08 (-0.5 to 0.67) |
| Schmidt et al. 2015 | Leg Press 1-RM (N.m) | RE | AE | 21 | 4.44 ± 31.28 | 20 | 2.3 ± 20.81 | 0.08 (-0.53 to 0.69) |
| Travier et al. 2015 | Isokinetic knee extension at 60 degrees (N.m) - Right | COMB | Controls | 87 | 4.4 ± 23.08 | 77 | -4.3 ± 23.73 | 0.37 (0.06 to 0.68) |
| Travier et al. 2015 | Isokinetic knee extension at 60 degrees (N.m) - Left | COMB | Controls | 87 | 6.7 ± 27.36 | 77 | -4.2 ± 28.65 | 0.39 (0.08 to 0.7) |
| van Waart et al. 2015 | Isometric knee extension (N.m) | COMB | Controls | 76 | 1.2 ± 18.12 | 77 | -3.4 ± 21.43 | 0.23 (-0.09 to 0.55) |
| Cornette et al. 2016 | Isometric knee extension (kg) | COMB | Controls | 22 | -1.3 ± 7.62 | 22 | -0.5 ± 8.35 | -0.1 (-0.69 to 0.49) |
| de Luca et al. 2016 | Leg Press 1-RM (kg) | COMB | Controls | 10 | 31.1 ± 37.19 | 10 | -6.5 ± 19.54 | 1.21 (0.24 to 2.18) |
| de Luca et al. 2016 | Knee Extension 1-RM (kg) | COMB | Controls | 10 | 9.4 ± 11.62 | 10 | -0.1 ± 12.44 | 0.76 (-0.16 to 1.67) |
| Dolan et al. 2016 | Leg Press 1-RM (%) | AE | Controls | 11 | 13.28 ± 7.9 | 10 | 0.34 ± 11.1 | 1.3 (0.34 to 2.26) |
| Dolan et al. 2016 | Leg Press 1-RM (%) | AE | Controls | 12 | 10.26 ± 10 | 10 | 0.34 ± 11.1 | 0.91 (0.02 to 1.8) |
| Hagstrom et al. 2016 | Leg Press 1-RM (kg) | RE | Controls | 20 | 40.04 ± 43.76 | 19 | 4.08 ± 43.92 | 0.8 (0.15 to 1.46) |
| Dieli-Conwright et al. & Dieli-Conwright et al. [2] 2018 & 2018 | Knee Extension 1-RM (kg) | COMB | Controls | 46 | 30.3 ± 10.7 | 45 | -2 ± 9.05 | 3.23 (2.6 to 3.86) |
| Mijwel et al. & Mijwel et al. 2018 & 2018 | Isometric knee extension (kg) [taxanes] | COMB | Controls | 38 | 16.32 ± 33.34 | 30 | -4.6 ± 21.06 | 0.72 (0.23 to 1.22) |
| Mijwel et al. & Mijwel et al. 2018 & 2018 | Isometric knee extension (kg) [taxanes] | AE | Controls | 40 | 8.26 ± 22.74 | 30 | -4.6 ± 21.06 | 0.58 (0.09 to 1.06) |
| Mijwel et al. & Mijwel et al. 2018 & 2018 | Isometric knee extension (kg) [taxanes] | COMB | AE | 38 | 16.32 ± 33.34 | 40 | 8.26 ± 22.74 | 0.28 (-0.17 to 0.73) |
| Mijwel et al. & Mijwel et al. 2018 & 2018 | Isometric knee extension (kg) [non-taxanes] | COMB | Controls | 27 | 12.01 ± 28.88 | 21 | -2.29 ± 31.44 | 0.47 (-0.11 to 1.05) |
| Mijwel et al. & Mijwel et al. 2018 & 2018 | Isometric knee extension (kg) [non-taxanes] | AE | Controls | 22 | 8.76 ± 26.06 | 21 | -2.29 ± 31.44 | 0.38 (-0.23 to 0.98) |
| Mijwel et al. & Mijwel et al. 2018 & 2018 | Isometric knee extension (kg) [non-taxanes] | COMB | AE | 27 | 12.01 ± 28.88 | 22 | 8.76 ± 26.06 | 0.12 (-0.45 to 0.68) |
| Ceseiko et al. & Ceseiko et al. 2019 & 2020 | Leg Press 1-RM (kg) | RE | Controls | 27 | 20.4 ± 8.35 | 28 | -8.9 ± 5.53 | 4.09 (3.14 to 5.04) |
| dos Santos et al. & Moraes et al. 2019 & 2021 | Leg Press 10-RM (kg) | RE | Controls | 12 | 33.75 ± 11.51 | 13 | -1.54 ± 6.58 | 3.68 (2.32 to 5.04) |
| Hiraoui et al. & Hiraoui et al. 2019 & 2023 | Isometric Knee Extension (kg) | COMB | Controls | 20 | 2.27 ± 2.6 | 12 | -0.73 ± 1.3 | 1.32 (0.53 to 2.12) |
| Santagnello et al. 2020 | Leg Press 1-RM (kg) | RE | Controls | 11 | 40.1 ± 5 | 9 | -1.8 ± 5.5 | 7.67 (4.89 to 10.46) |
| Owusu et al. 2022 | Leg Press 1-RM (lbs) | COMB | Controls | 82 | 26.1 ± 41.35 | 78 | 8.1 ± 33.12 | 0.48 (0.16 to 0.79) |
| Winters-Stone et al. 2022 | Leg Press 1-RM (kg) | RE | Controls | 32 | 8.2 ± 9.96 | 29 | 5.4 ± 10.23 | 0.27 (-0.23 to 0.78) |
| Winters-Stone et al. 2022 | Leg Press 1-RM (kg) | AE | Controls | 27 | 2.7 ± 10.21 | 29 | 5.4 ± 10.23 | -0.26 (-0.79 to 0.27) |
| Winters-Stone et al. 2022 | Leg Press 1-RM (kg) | RE | AE | 32 | 8.2 ± 9.96 | 27 | 2.7 ± 10.21 | 0.54 (0.02 to 1.06) |
| Damato et al. 2023 | Isometric knee extension (kg) - left | RE | Controls | 18 | 3.17 ± 8.99 | 17 | -5.98 ± 7.02 | 1.1 (0.39 to 1.82) |
| Damato et al. 2023 | Isometric knee extension (kg) - right | RE | Controls | 18 | 2.45 ± 8.66 | 17 | -4.63 ± 8.63 | 0.8 (0.11 to 1.49) |
| Soriano-Maldonado et al. 2023 | Lower body muscular strength (z-score) | RE | Controls | 32 | 0.33 ± 0.74 | 28 | -0.38 ± 0.74 | 0.94 (0.41 to 1.48) |
| Bettariga et al. 2025 | Leg Press 1-RM (kg) | RE | AE | 14 | 24.1 ± 17.47 | 14 | 16.1 ± 5.15 | 0.6 (-0.16 to 1.36) |

95% CI, 95% confidence interval; AE, aerobic exercise; COMB, combined resistance and aerobic exercise; MD, within-group mean difference; RE, resistance exercise; SMD, standardised mean difference.

**Table S5.** Characteristics of studies examining the effects of aerobic exercise, resistance exercise and combined resistance and aerobic exercise on lean body mass, lower-limb muscle hypertrophy, lower-limb muscle strength, physical function and fatigue in women diagnosed with breast cancer.

| **Author** | **Participant characteristics** | **Treatment characteristics** | **Study design** | **Intervention characteristics** | **Retention** | **Outcomes** |
| --- | --- | --- | --- | --- | --- | --- |
| Nieman et al. 1995 | 16 women previously treated for breast cancer  Age: range of 35 to 72 years  BMI: NR  Stage 3-4: NR | Surgery: 100%; Radiotherapy: NR; Chemotherapy: 100%; Hormone therapy: 0% | Combined resistance and aerobic exercise vs. Control group | **Combined resistance and aerobic exercise**  3 sessions per week for 12 weeks performing a supervised program undertaking 2 sets per exercise in 7 resistance exercises, in addition to 3 sessions per week performing continuous aerobic exercise for 30 min per session at 75% of HR maximum | 12 out of 16 participants completed the study (75.0%) | Physical function;  Lower-body muscle strength |
| Segal et al. 2001 | 123 women undergoing treatment for breast cancer  Age: Mean of 50.9 years  BMI: NR  Stage 3-4: 0% | Surgery: NR; Radiotherapy: NR; Chemotherapy: 78.1% | Aerobic exercise vs. Usual care | **Aerobic exercise**  5 sessions per week for 26 weeks performing supervised or non-supervised aerobic exercise at 60% of predicted VO2 max | 99 out of 123 participants completed the study (80.5%) | Fatigue |
| Burnham et al. 2002 | 21 women previously treated for breast cancer  Age: Mean of 53.6 years  BMI: NR  Stage 3-4: 0% | Surgery: 52.4%; Radiotherapy: 47.6%; Chemotherapy: 90.5%; Hormone therapy: NR | Aerobic exercise vs. Control group | **Aerobic exercise**  3 sessions per week for 10 weeks performing a supervised aerobic exercise at 60% of HR max | 18 out of 21 participants completed the study (85.7%) | Fatigue |
| Courneya et al. 2003 | 53 women previously treated for breast cancer  Age: Mean of 59 years  BMI: Mean of 29.2 kg.m^-2^  Stage 3-4: 6% | Surgery: 100%; Radiotherapy: 71%; Chemotherapy: 40%; Hormone therapy: 46% | Aerobic exercise vs. Waitlist Control group | **Aerobic exercise**  3 sessions per week for 15 weeks performing supervised continuous aerobic exercise for 15 to 35 min at 75% of predicted VO2 max | 52 out of 53 participants completed the study (98.1%) | Fatigue |
| Pinto et al. 2003 | 24 women previously treated for breast cancer  Age: Mean of 52.5 years  BMI: Mean of 26.8 kg.m^-2^  Stage 3-4: 0% | Surgery: 96%; Radiotherapy: 65%; Chemotherapy: 30%; Hormone therapy: 61% | Aerobic exercise vs. Control group | **Aerobic exercise**  3 sessions per week for 12 weeks performing supervised continuous aerobic exercise for 30 min at 70% of HR peak | 21 out of 24 participants completed the study (87.5%) | Fatigue |
| Campbell et al. 2005 | 22 women undergoing treatment for breast cancer  Age: Mean of 47.5 years  BMI: NR  Stage 3-4: NR | Surgery: 100%; Radiotherapy: 72.7%; Chemotherapy: 72.7%; Hormone therapy: NR | Combined resistance and aerobic exercise vs. Usual care | **Combined resistance and aerobic exercise**  2 sessions per week for 12 weeks performing a supervised program, in addition to 2 sessions per week performing continuous aerobic exercise | 19 out of 22 participants completed the study (86.4%) | Fatigue;  Physical function |
| Drouin et al. 2005 | 23 women undergoing treatment for breast cancer  Age: Mean of 50 years  BMI: NR  Stage 3-4: 38.1% | Surgery: 100%; Radiotherapy: 100%; Chemotherapy: 76.2%; Hormone therapy: NR | Aerobic exercise vs. Stretching control group | **Aerobic exercise**  3 to 5 sessions per week for 7 weeks performing non-supervised continuous aerobic exercise for 20 to 45 min at 70% of HR maximum. | 21 out of 23 participants completed the study (91.3%) | Fatigue |
| Mock et al. 2005 | 119 women undergoing treatment for breast cancer  Age: Mean of 51.5 years  BMI: Mean of 25.7 kg.m^-2^  Stage 3-4: 3.4% | Surgery: 100%; Radiotherapy: 58%; Chemotherapy: 42%; Hormone therapy: NR | Aerobic exercise vs. Usual care | **Aerobic exercise**  5 to 6 sessions per week during  cancer treatment, performing non-supervised continuous aerobic exercise for 15 to 30 min at 70% of HR maximum. | 108 out of 119 participants completed the study (90.8%) | Fatigue |
| Pinto et al. 2005 | 86 women previously treated for breast cancer  Age: Mean of 53.1 years  BMI: Mean of 28 kg.m^-2^  Stage 3-4: 0% | Surgery: 27.9%; Radiotherapy: 68.6%; Chemotherapy: 55.8%; Hormone therapy: 61.6% | Aerobic exercise vs. Control group | **Aerobic exercise**  2 to 5 sessions per week for 12 weeks performing non-supervised continuous aerobic exercise for 10 to 30 min at 65% of HR maximum. | 82 out of 86 participants completed the study (95.3%) | Fatigue |
| Schmitz et al. 2005 & Ahmed et al. 2006 | 85 women previously treated for breast cancer  Age: Mean of 53 years  BMI: Mean of 25.8 kg.m^-2^  Stage 3-4: 4.9% | Surgery: NR  Radiotherapy: 50.6%; Chemotherapy: 67.9%; Hormone therapy: 81.5% | Resistance exercise vs. Delayed intervention group | **Resistance exercise**  2 sessions per week for 26 weeks performing a supervised and a non-supervised program undertaking 1 to 3 sets per exercise in 9 resistance exercises. The peak intensity exercise is 10RM, with 75% of peak 1RM. | 69 out of 85 participants completed the study (81.2%) | Lean mass;  Lower-body muscle strength |
| Battaglini et al. 2006 | 20 women undergoing treatment for breast cancer  Age: Mean of 53.6 years  BMI: NR  Stage 3-4:NR | Surgery: NR  Radiotherapy: 100%; Chemotherapy: 100%; Hormone therapy: 81.5% | Combined resistance and aerobic exercise vs. Control group | **Combined resistance and aerobic exercise**  2 sessions per week for 20 weeks performing a supervised program undertaking 1 to 3 sets per exercise in 11 resistance exercises, 60% of 1 RM, in addition to 2 sessions per week performing continuous aerobic exercise for 6 to 12 min per session. | 20 out of 20 participants completed the study (100%) | Fatigue |
| Herrero et al. 2006 | 20 women previously treated for breast cancer  Age: Mean of 50.5 years  BMI: Mean of 24.6 kg.m^-2^  Stage 3-4: 0% | Surgery: 100%  Radiotherapy: 100%; Chemotherapy: 100%; Hormone therapy: 0% | Combined resistance and aerobic exercise vs. Control group | **Combined resistance and aerobic exercise**  3 sessions per week for 8 weeks performing a supervised program undertaking 1 to 3 sets per exercise of 8RM in 11 resistance exercises, in addition to 3 sessions per week performing continuous aerobic exercise for 20 to 30 min per session at 80% of HR maximum | 16 out of 20 participants completed the study (80%) | Lean mass;  Physical function |
| Courneya et al. 2007 | 242 women undergoing treatment for breast cancer  Age: Mean of 49.2 years  BMI: Mean of 26.6 kg.m^-2^  Stage 3-4: 14.5% | Surgery: 59.1%  Radiotherapy: NR Chemotherapy:100%; Hormone therapy: NR | Resistance exercise vs Usual care | **Resistance exercise**  3 sessions per week for the completion chemotherapy performing a supervised program undertaking 2 sets per exercise in 9 resistance exercises with 70% of peak 1RM  **Aerobic exercise**  3 sessions per week for the completion chemotherapy performing supervised continuous aerobic exercise for 15 to 45 min at 80% of VO_2_ max. | 224 out of 242 participants completed the study (92.6%) | Fatigue;  Lean mass;  Lower-body muscle strength |
| Daley et al. 2007 | 72 women previously treated for breast cancer  Age: Mean of 51.3 years  BMI: Mean of 29.0 kg.m^-2^  Stage 3-4: 0% | Surgery: 100%; Radiotherapy: 79.2%; Chemotherapy: 77.8%; Hormone therapy: NR; | Aerobic exercise vs. Usual care | **Aerobic exercise**  3 sessions per week for 8 weeks performing supervised continuous aerobic exercise for 50 min at 85% of HR maximum. | 62 out of 72 participants completed the study (86.1%) | Fatigue |
| Matthews et al. 2007 | 36 women previously treatment for breast cancer;  Age: Mean of 53.5 years;  BMI: Mean of 28.9 kg.m^-2^  Stage 3-4:NR; | Surgery: 22.2%; Radiotherapy: 63.9%; Chemotherapy: 30.6%; Hormone therapy: 52.8%; | Aerobic exercise vs. Waitlist control group | **Aerobic exercise**  3 to 5 sessions per week for 12 weeks performing non-supervised continuous aerobic exercise for 20 to 40 min at 11-13 out of 20 RPE | 36 out of 36 participants completed the study (100%) | Lean mass |
| Mutrie et al. 2007 | 203 women undergoing treatment for breast cancer;  Age: Mean of 51.6 years;  BMI: Mean of 27.3 kg.m^-2^  Stage 3-4:NR | Surgery: 100%; Radiotherapy: 92.5%; Chemotherapy: 71.6%; Hormone therapy: NR; | Combined resistance and aerobic exercise vs. Usual care | **Combined resistance and aerobic exercise**  2 sessions per week for 12 weeks performing a supervised program, in addition to 2 sessions per week performing continuous aerobic at 75% of HR maximum | 174 out of 203 participants completed the study (85.7%) | Fatigue;  Physical function |
| Nikander et al. 2007 | 30 women with undergoing treatment for breast cancer;  Age: Mean of 51.9 years;  BMI: Mean of 27.5 kg.m^-2^  Stage 3-4: 0 %; | Surgery: 100%; Radiotherapy: NR; Chemotherapy: 78.6%; Hormone therapy: 75%; | Aerobic exercise vs. Control Group | **Combined resistance and aerobic exercise**  1 session per week for 12 weeks performing a supervised program, in addition to 1 session per week performing continuous aerobic at 14-16 out of 20 RPE | 28 out of 30 participants completed the study (93.3%) | Lower-body muscle strength |
| Schwartz et al. 2007 | 72 women undergoing treatment for breast cancer;  Age: Mean of 49.1 years;  BMI: NR  Stage 3-4: 15.9% | Surgery: 100%; Radiotherapy: 64.7%; Chemotherapy: 100%; Hormone therapy: NR; | Resistance exercise vs. Aerobic exercise vs. Control group | **Resistance exercise**  4 sessions per week for 26 weeks performing a non-supervised program undertaking 2 sets per exercise at 75% of 1-RM in 8 resistance exercises.  **Aerobic exercise**  4 sessions per week for 26 weeks performing a non-supervised program undertaking continuous aerobic exercise at 15 to 30 min. | 66 out of 72 participants completed the study (91.6%) | Physical function;  Lower-body muscle strength |
| Yuen et al. 2007 | 22 women previously treatment for breast cancer;  Age: Mean of 53.8 years;  BMI: NR  Stage 3-4: NR | Surgery: 100%; Radiotherapy: 77.3%; Chemotherapy: 81.8%; Hormone therapy: NR; | Resistance exercise vs. Aerobic exercise vs. Usual care | **Resistance exercise**  3 sessions per week for 12 weeks performing a non-supervised program undertaking 1 to 2 sets at 70% of 1-RM per exercise in 8 resistance exercises.  **Aerobic exercise**  3 sessions per week for 12 weeks performing a non-supervised program undertaking continuous aerobic exercise at 15 to 30 min. | 22 out of 22 participants completed the study (100%) | Fatigue;  Physical function |
| Hwang et al. 2008 | 40 women undergoing treatment for breast cancer;  Age: Mean of 46.3 years;  BMI: NR  Stage 3-4: %; | Surgery: 100%; Radiotherapy: 100%; Chemotherapy: NR; Hormone therapy: NR; | Combined resistance and aerobic exercise vs. Control Group | **Combined resistance and aerobic exercise**  3 sessions per week for 5 weeks performing a supervised program in addition to 3 sessions per week performing continuous aerobic exercise. | 37 out of 40 participants completed the study (92.5%) | Fatigue |
| Milne et al. 2008 | 58 women previously treatment for breast cancer;  Age: Mean of 55.1 years;  BMI: Mean of 26.3 kg.m^-2^  Stage 3-4: 3.4%; | Surgery: 51.7%; Radiotherapy: 60.3%; Chemotherapy: 70.7%; Hormone therapy: 74.1%; | Combined resistance and aerobic exercise vs. Delayed intervention group | **Combined resistance and aerobic exercise**  3 sessions per week for 12 weeks performing a supervised program undertaking 2 sets per exercise in 12 resistance exercises, in addition to 3 sessions per week performing continuous aerobic exercise. | 58 out of 58 participants completed the study (100%) | Fatigue;  Lower-body muscle strength |
| Portela et al. 2008 | 44 women previously treatment for breast cancer;  Age: Mean of 52.9 years;  BMI: NR  Stage 3-4: 29.4%; | Surgery: 100%; Radiotherapy: NR; Chemotherapy: NR; Hormone therapy: NR; | Combined resistance and aerobic exercise vs. Combined resistance and aerobic exercise vs. Control Group | **Combined resistance and aerobic**  3 sessions per week for 26 weeks performing a supervised or non-supervised program undertaking 2 sets at 13-15 out of 20 RPE in 5 resistance exercises and 3 sessions per week performing a treadmill continuous aerobic exercise for 30 min per session at 80% of HR maximum. | 34 out of 44 participants completed the study (77.3%) | Physical function |
| Cadmus et al. 2009 | 105 women undergoing and previously treatment (mixed), with 47.6% currently treated for breast cancer;  Age: Mean of 55.1 years;  BMI: Mean of 29.2 kg.m^-2^  Stage 3-4: 6% and 12% | Surgery: 100% and 100%  Radiotherapy: 66% and 72%  Chemotherapy: 64% and 58%  Hormone therapy: 62% and 63.5% | Aerobic exercise vs. Usual care | **Aerobic exercise**  5 sessions per week for 26 weeks performing supervised or non-supervised continuous aerobic exercise. | 105 out of 105 participants completed the study (100%) | Fatigue |
| Cadmus et al. 2009 & Irwin et al. 2009 | 75 women previously treatment for breast cancer;  Age: Mean of 55.7 years;  BMI: NR  Stage 3-4: 12.05%; | Surgery: NR; Radiotherapy: 71.4%; Chemotherapy: 58.1%; Hormone therapy: 63.6%; | Aerobic exercise vs. Usual care | **Aerobic exercise**  5 sessions per week for 26 weeks performing supervised continuous aerobic exercise. | 75 out of 75 participants completed the study (100%) | Fatigue;  Lean mass |
| Schmitz et al. 2009 | 141 women previously treatment for breast cancer;  Age: Mean of 56.9 years;  BMI: Mean of 30.4 kg.m^-2^  Stage 3-4: 31% | Surgery: 100%; Radiotherapy: 79.5%; Chemotherapy: 81.5%; Hormone therapy: 12.6%; | Resistance exercise vs. Control Group | **Resistance exercise**  2 sessions per week for 52 weeks performing a supervised and a non-supervised program undertaking 2 to 3 sets of 10RM per exercise in 9 resistance exercises. | 130 out of 141 participants completed the study (92.2%) | Lean mass;  Lower-body muscle strength |
| Moros et al. 2010 | 22 women undergoing treatment for breast cancer;  Age: Mean of 49 years;  BMI: Mean of 27.8 kg.m^-2^  Stage 3-4: 9% | Surgery: 100%; Radiotherapy: NR; Chemotherapy: 100%; Hormone therapy: NR; | Combined resistance and aerobic exercise vs. Control Group | **Combined resistance and aerobic exercise**  3 sessions per week for 18 to 22 weeks performing a supervised resistance exercise program & 3 sessions per week performing cycle continuous aerobic exercise for per session at 70% of HR maximum | 22 out of 22 participants completed the study (100%) | Fatigue |
| Schmitz et al. 2010 | 154 women previously treatment for breast cancer;  Age: Mean of 55 years;  BMI: Mean of 28.0 kg.m^-2^  Stage 3-4: 34.5% | Surgery: 100%; Radiotherapy: 76%; Chemotherapy: 71%; Hormone therapy: 20.5%; | Resistance exercise vs. Control Group | **Resistance exercise**  2 sessions per week for 52 weeks performing a supervised and a non-supervised program undertaking 3 sets per exercise in 9 resistance exercises. The peak intensity exercise is 10RM, with 75% of peak 1RM | 134 out of 154 participants completed the study (87%) | Lean mass;  Lower-body muscle strength |
| Cantarero-Villanueva et al. 2011 | 78 women previously treatment for breast cancer;  Age: Mean of 48.4 years;  BMI: NR  Stage 3-4: 17.8% | Surgery: 100%; Radiotherapy: 91.1%; Chemotherapy: 97.1%; Hormone therapy: NR; | Combined resistance and aerobic exercise vs. Control Group | **Combined resistance and aerobic exercise** 3 sessions per week for 8 weeks performing a Supervised program undertaking 2 to 3 sets at 75% of 1-RM in 10 resistance exercises and 3 sessions per week performing aerobic exercise for 10 to 25 min per session at | 67 out of 78 participants completed the study (85.9) | Fatigue |
| DeNysschen et al. 2011 | 70 women undergoing treatment for breast cancer;  Age: Mean of 49.9 years;  BMI: NR  Stage 3-4: 14.9%; | Surgery: NR; Radiotherapy: 68%; Chemotherapy: 100%; Hormone therapy: NR; | Aerobic exercise vs. Usual care | **Aerobic exercise**  3 to 5 sessions per week after the completion chemotherapy performing supervised and non-supervised continuous aerobic exercise for 20 to 30 min at 12-14 out of 20 RPE | 70 out of 70 participants completed the study (100%) | Lean mass |
| Winters-Stone et al. 2011 & Winters-Stone et al. 2012 | 106 women previously treatment for breast cancer;  Age: Mean of 62.2 years;  BMI: Mean of 29.5 kg.m^-2^  Stage 3-4: 5.6 % | Surgery: NR; Radiotherapy: 87.7%; Chemotherapy: 60.4%; Hormone therapy: 56.6%; | Resistance exercise vs. Stretching control group | **Resistance exercise**  2 sessions per week for 52 weeks performing a supervised and a non-supervised program undertaking 1 to 3 sets per exercise in 9 resistance exercises. The peak intensity exercise is 10RM, with 70% of 1RM | 67 out of 106 participants completed the study (63.2%) | Fatigue;  Lean mass;  Physical function;  Lower-body muscle strength |
| Anderson et al. 2012 | 104 women undergoing treatment for breast cancer;  Age: NR  BMI: NR  Stage 3-4: 11.5%; | Surgery: 100%; Radiotherapy: 64.5%; Chemotherapy: 60%; Hormone therapy: 47%; | Combined resistance and aerobic exercise vs. Usual care | **Combined resistance and aerobic exercise**  2 sessions per week for 39 weeks performing a hybrid program undertaking sets at 50% of 1-RM in 9 resistance exercises and 2 sessions per week performing Continuous aerobic exercise for 20 to 30 min per session at 14-16 out of 20 RPE | 78 out of 104 participants completed the study (75%) | Physical function |
| Duijtis et al. 2012 | 207 women previously treatment for breast cancer;  Age: Mean of 47.7 years;  BMI: Mean of 25.2 kg.m^-2^  Stage 3-4: 0 %; | Surgery: 47.8%; Radiotherapy: NR; Chemotherapy: 88.4%; Hormone therapy: 86.5%; | Aerobic exercise vs. Control Group | **Aerobic exercise**  3 to 5 sessions per week for 12 weeks performing supervised continuous aerobic exercise for 150 to 180 min per week at 80% HR maximum | 176 out of 207 participants completed the study (85%) | Fatigue |
| Naumann et al. 2012 | 23 women previously treatment for breast cancer;  Age: Mean of 50.3 years;  BMI: NR  Stage 3-4: 0%; | Surgery: 94.8%; Radiotherapy: 71%; Chemotherapy: 66.8%; Hormone therapy: 100%; | Combined resistance and aerobic exercise vs. Control Group | **Combined resistance and aerobic exercise**  3 sessions per week for 8 weeks performing a supervised program at 14-16 out of 20 RPE in resistance exercises & 3 sessions per week performing continuous aerobic exercise for 50 min per session at moderate intensity | 21 out of 23 participants completed the study (91.3%) | Fatigue;  Lower-body muscle strength |
| Nikander et al. 2012 & Saarto et al. 2012 & Saarto et al. 2012 | 86 women undergoing treatment for breast cancer;  Age: Mean of 53.1 years;  BMI: NR  Stage 3-4: 24.3% | Surgery: 100%; Radiotherapy: 78.5%; Chemotherapy: 89%; Hormone therapy: 88%; | Combined resistance and aerobic exercise vs. Control Group | **Combined resistance and aerobic exercise**  1 session per week for 52 weeks performing a hybrid program undertaking sets at 14-16 out of 20 RPE in resistance exercises & 1 sessions per week performing aerobic exercise for per session | 67 out of 86 participants completed the study (77.9%) | Fatigue;  Lean mass;  Lower-body muscle strength |
| Cormie et al. 2013 | 62 women previously treatment for breast cancer;  Age: Mean of 57.1 years;  BMI: Mean of 29.8 kg.m^-2^  Stage 3-4: 19.3% | Surgery: 93.6%; Radiotherapy: 82.3%; Chemotherapy: 82.3%; Hormone therapy: 62.9%; | Resistance exercise vs. Control Group | **Resistance exercise** & **resistance exercise**  2 sessions per week for 12 weeks performing a supervised program undertaking 1 to 4 sets per exercise in 8 exercises at 85% of 1-RM or 65% of 1-RM | 57 out of 62 participants completed the study (91.9%) | Fatigue;  Lower-body muscle strength |
| Courneya et al. 2013 | 301 women undergoing treatment for breast cancer;  Age: Mean of 50.1 years;  BMI: Mean of 26.5 kg.m^-2^  Stage 3-4: 10.3% | Surgery: 100%; Radiotherapy: NR; Chemotherapy: 100%; Hormone therapy: NR; | Aerobic exercise vs. Aerobic exercise vs. Combined resistance and aerobic exercise | **Combined resistance and aerobic exercise**  3 sessions per week for the completion of chemotherapy performing a supervised program undertaking 2 sets per exercise in 9 exercises at 75% of 1 RM, in addition and a supervised continuous aerobic exercise for 15 to 30 min at 75% of VO2 peak.  **Aerobic exercise**  3 sessions per week for the completion of chemotherapy performing a supervised continuous aerobic exercise for 15 to 30 min or 15 to 60 min at 75% of VO2 peak. | 298 out of 301 participants completed the study (99%) | Fatigue;  Lean mass;  Lower-body muscle strength |
| Ergun et al. 2013 | 60 women previously treatment for breast cancer;  Age: Mean of 51.6 years;  BMI: Mean of 25.2 kg.m^-2^  Stage 3-4: %; | Surgery: 100%; Radiotherapy: 100%; Chemotherapy: 100%; Hormone therapy: NR; | Combined resistance and aerobic exercise vs. Aerobic exercise vs. Education control group | **Combined resistance and aerobic exercise**  3 sessions per week for 12 weeks performing a supervised program in resistance exercises and 3 sessions per week walking for 30 min per session  **Aerobic exercise**  3 sessions per week for 12 weeks performing a non-supervised walking for 30 min. | 58 out of 60 participants completed the study (96.7%) | Fatigue |
| Fernandez-Lao 2013 | 65 women previously treatment for breast cancer;  Age: Mean of 48.4 years;  BMI: Mean of 27.1 kg.m^-2^  Stage 3-4: 25%; | Surgery: 100%; Radiotherapy: NR; Chemotherapy: NR; Hormone therapy: 100%; | Combined resistance and aerobic exercise vs. Control Group | **Combined resistance and aerobic exercise**  3 sessions per week for 8 weeks performing a supervised program undertaking 2 to 3 sets in resistance exercises & 3 sessions per week performing aerobic exercise for per session at 60% of HR maximum | 65 out of 65 participants completed the study (100%) | Lean mass |
| Hayes et al. 2013 | 194 women undergoing treatment for breast cancer;  Age: Mean of 52.3 years;  BMI: NR  Stage 3-4: 0%; | Surgery: 100%; Radiotherapy: 38.6%; Chemotherapy: 60.3%; Hormone therapy: 58.8%; | Combined resistance and aerobic exercise vs. Usual care | **Combined resistance and aerobic exercise**  1 per week to 1 per month sessions for 35 weeks performing a supervised or non-supervised program undertaking resistance exercises and aerobic exercise | 180 out of 194 participants completed the study (92.8%) | Fatigue |
| Kulkarni et al. 2013 | 60 women undergoing treatment for breast cancer;  Age: Mean of 45.5 years;  BMI: Mean of 20.1 kg.m^-2^  Stage 3-4: 0%; | Surgery: 100%; Radiotherapy: 100%; Chemotherapy: NR; Hormone therapy: NR; | Aerobic exercise vs. Conventional physiotherapy | **Aerobic exercise**  5 sessions per week for 6 weeks performing supervised program, with treadmill continuous aerobic exercise for 30 to 50 min at 60% of HR reserve | 54 out of 60 participants completed the study (90%) | Fatigue |
| Milecki et al. 2013 | 66 women undergoing treatment breast cancer;  Age: Mean of 52.4 years;  BMI: Mean of 25.3 kg.m^-2^  Stage 3-4: 0% | Surgery: 100%; Radiotherapy: 100%; Chemotherapy: NR; Hormone therapy: NR; | Aerobic exercise vs. Usual care | **Aerobic exercise**  5 sessions per week for 6 weeks performing supervised program, with cycle continuous aerobic exercise for 40 to 45 min at 70% of HR maximum | 66 out of 66 participants completed the study (100%) | Physical function |
| Rogers et al. 2013 | 28 women previously treatment for breast cancer;  Age: Mean of 56 years;  BMI: Mean of 32.2 kg.m^-2^  Stage 3-4: 14% | Surgery: 100%; Radiotherapy: 79%; Chemotherapy: 75%; Hormone therapy: 50%; | Combined resistance and aerobic exercise vs. Usual care | **Combined resistance and aerobic exercise**  2 sessions per week for 12 weeks performing a hybrid program undertaking 1 set at in 8 resistance exercises & performing Mixed Continuous aerobic exercise for 150 min per week per session at Moderate intensity | 20 out of 28 participants completed the study (71.4%) | Fatigue |
| Hornsby et al. 2014 | 20 women undergoing treatment for breast cancer;  Age: Mean of 48.5 years;  BMI: Mean of 28.5 kg.m^-2^  Stage 3-4: 0% | Surgery: NR; Radiotherapy: NR; Chemotherapy: 100%; Hormone therapy: NR; | Aerobic exercise vs. Control group | **Aerobic exercise**  3 sessions per week for 12 weeks performing supervised program, with cycle interval aerobic exercise for 15 to 45 min at 100% of peak workload | 19 out of 20 participants completed the study (95%) | Fatigue |
| Husebo et al. 2014 | 67 women undergoing treatment for breast cancer;  Age: Mean of 52.2 years;  BMI: NR  Stage 3-4: 11.6% | Surgery: 100%; Radiotherapy: 71.7%; Chemotherapy: 100%; Hormone therapy: NR; | Combined resistance and aerobic exercise vs. Control group | **Combined resistance and aerobic exercise**  3 sessions per week for completion of chemotherapy weeks performing a non-supervised program in resistance exercises & 3 sessions per week performing continuous aerobic exercise | 60 out of 67 participants completed the study (89.6) | Fatigue;  Physical function |
| Murtezani et al. 2014 | 73 women previously treatment for breast cancer;  Age: Mean of 52 years;  BMI: Mean of 26 kg.m^-2^  Stage 3-4: 8% | Surgery: 100%; Radiotherapy: 60%; Chemotherapy: 58%; Hormone therapy: 55%; | Aerobic exercise vs. Control group | **Aerobic exercise**  3 sessions per week for 10 weeks performing supervised program, with mixed continuous aerobic exercise for 15 to 35 min at 75% HR reserve | 62 out of 73 participants completed the study (84.9%) | Physical function |
| Rogers et al. 2014 | 46 women previously treatment for breast cancer;  Age: Mean of 56.2 years;  BMI: Mean of 31 kg.m^-2^  Stage 3-4: 0% | Surgery: 100%; Radiotherapy: 63.6%; Chemotherapy: 40.9%; Hormone therapy: 52.3%; | Combined resistance and aerobic exercise vs Control group | **Combined resistance and aerobic exercise**  2 sessions per week for 12 weeks performing a hybrid program undertaking 2 sets at in 8 resistance exercises & 4 sessions per week performing mixed continuous aerobic exercise for 40 min per session at 52% of HR reserve | 42 out of 46 participants completed the study (91.3%) | Fatigue |
| Steindorf et al. 2014 & Wiskemann et al. 2017 | 160 women undergoing treatment for breast cancer;  Age: Mean of 55.8 years;  BMI: Mean of 27.2 kg.m^-2^  Stage 3-4: 9.6%; | Surgery: 100%; Radiotherapy: 100%; Chemotherapy: 35.5%; Hormone therapy: 48.4%; | Resistance exercise vs. Relaxation control | **Resistance exercise**  2 sessions per week for 12 weeks performing a supervised program undertaking 3 sets per exercise in 8 resistance exercises at 80% of 1RM | 155 out of 160 participants completed the study (96.9%) | Fatigue;  Lower-body muscle strength |
| Al-Majid et al. 2015 | 14 women undergoing treatment for breast cancer;  Age: Mean of 50.3 years;  BMI: NR  Stage 3-4: 0%; | Surgery: NR; Radiotherapy: NR; Chemotherapy: 100%; Hormone therapy: NR; | Aerobic exercise. vs. Usual care | **Aerobic exercise**  2 to 3 sessions per week for 12 weeks performing supervised program, with treadmill continuous aerobic exercise for 20 to 40 min at 80% HR reserve | 14 out of 14 participants completed the study (100%) | Fatigue |
| Do et al. 2015 [1] | 212 women undergoing treatment for breast cancer;  Age: Mean of 47.6 years;  BMI: NR  Stage 3-4: 11.2% | Surgery: 100%; Radiotherapy: 100%; Chemotherapy: 46.8%; Hormone therapy: 56.5%; | Combined resistance and aerobic exercise vs. Delayed intervention group | **Combined resistance and aerobic exercise**  5 sessions per week for 4 weeks performing a supervised program undertaking 2 sets at 80% of 1-RM in 6 resistance exercises & 5 sessions per week performing mixed continuous aerobic exercise for 40 min per session at 75% of VO2 max | 62 out of 212 participants completed the study (29.2%) | Fatigue;  Lower-body muscle strength |
| Do et al. 2015 [2] | 46 women previously treatment for breast cancer;  Age: Mean of 49.6 years;  BMI: NR  Stage 3-4: 18.1% | Surgery: 100%; Radiotherapy: NR; Chemotherapy: 77.3%; Hormone therapy: 79.5%; | Resistance exercise vs. Control group | **Resistance exercise**  5 sessions per week for 8 weeks performing a supervised program undertaking 3 sets per exercise in 8 resistance exercises at 6 out of 10 RPE | 44 out of 46 participants completed the study (95.7%) | Fatigue |
| Naraphong et al. 2015 | 23 women undergoing treatment for breast cancer;  Age: Mean of 46.7 years;  BMI: NR  Stage 3-4: 26% | Surgery: 100%; Radiotherapy: NR; Chemotherapy: 100%; Hormone therapy: NR; | Aerobic exercise vs. Usual care | **Aerobic exercise**  1 session per week for 12 weeks performing non-supervised program, with continuous aerobic exercise for 20 to 30 min at 60% HR maximum | 21 out of 23 participants completed the study (91.3%) | Fatigue |
| Schmidt et al. 2015 [1] | 81 women undergoing treatment for breast cancer;  Age: Mean of 54.3 years;  BMI: NR  Stage 3-4: 0%; | Surgery: 100%; Radiotherapy: NR; Chemotherapy: 100%; Hormone therapy: NR; | Resistance exercise vs. Aerobic exercise vs.  Usual care | **Resistance exercise**  2 sessions per week for 12 weeks performing a supervised program undertaking 1 set per exercise in 10 resistance exercises at 50% of 1RM.  **Aerobic exercise**  2 sessions per week for 12 weeks performing supervised program, with cycle continuous aerobic exercise for 25 to 30 min at 11-14 out 20 RPE. | 67 out of 81 participants completed the study (82.7%) | Fatigue;  Lower-body muscle strength |
| Schmidt et al. 2015 [2] | 101 women undergoing treatment for breast cancer;  Age: Mean of 52.7 years;  BMI: Mean of 26 kg.m^-2^  Stage 3-4: 17.9%; | Surgery: 100%; Radiotherapy: NR; Chemotherapy: 100%; Hormone therapy: NR; | Resistance exercise vs Relaxation control | **Resistance exercise**  2 sessions per week for 12 weeks performing a supervised program undertaking 3 sets per exercise in 8 resistance exercises at 80% of 1RM | 95 out of 101 participants completed the study (94%) | Fatigue |
| Travier et al. 2015 | 204 women undergoing treatment for breast cancer;  Age: Mean of 49.6 years;  BMI: Mean of 26.2 kg.m^-2^  Stage 3-4: 0%; | Surgery: NR; Radiotherapy: 30.4%; Chemotherapy: 100%; Hormone therapy: NR; | Combined resistance and aerobic exercise vs. Usual care | **Combined resistance and aerobic exercise**  2 sessions per week for 18 weeks performing a Supervised program undertaking 2 sets at 75% of 1-RM in resistance exercises & 2 sessions per week performing Cycle Interval aerobic exercise for 3 bouts of 2 min per session | 164 out of 204 participants completed the study (80.3%) | Fatigue;  Lower-body muscle strength |
| van Waart et al. 2015 | 153 women undergoing treatment for breast cancer;  Age: Mean of 50.7 years;  BMI: NR  Stage 3-4: 48% | Surgery: 75.8%; Radiotherapy: 78.4%; Chemotherapy: 100%; Hormone therapy: NR; | Combined resistance and aerobic exercise vs. Usual care | **Combined resistance and aerobic exercise**  2 sessions per week for completion of chemotherapy weeks performing a supervised program undertaking 2 sets at 80% of 1-RM in 6 resistance exercises & 2 sessions per week performing continuous aerobic exercise for 30 min per session at 80% of maximal workload | 140 out of 153 participants completed the study (91.5%) | Fatigue;  Physical function;  Lower-body muscle strength |
| Bruno et al. 2016 | 38 women previously treatment for breast cancer;  Age: Mean of 56.4 years;  BMI: Mean of 24.9 kg.m^-2^  Stage 3-4: 12.9% | Surgery: NR; Radiotherapy: NR; Chemotherapy: NR; Hormone therapy: 51.6%; | Aerobic exercise vs. Recommendation | **Aerobic exercise**  2 sessions per week for 12 weeks performing supervised and non-supervised program, with mixed continuous aerobic exercise for 50 min at moderate intensity | 38 out of 38 participants completed the study (100%) | Lean mass |
| Buchan et al. 2016 | 41 women previously treatment for breast cancer;  Age: Mean of 56 years;  BMI: Mean of 29 kg.m^-2^  Stage 3-4: 0%; | Surgery: NR; Radiotherapy: 87.8%; Chemotherapy: 92.7%; Hormone therapy: 58.5%; | Resistance exercise vs. Aerobic exercise | **Resistance exercise**  1 to 2 sessions per week for 12 weeks performing a supervised and non-supervised program undertaking 2 sets per exercise in 6 to 12 resistance exercises at 12-14 out of 20 RPE  **Aerobic exercise**  1 to 2 sessions per week for 12 weeks performing a supervised and non-supervised program with mixed continuous aerobic exercise for 50 min | 39 out of 41 participants completed the study (95.1%) | Lean mass;  Physical function |
| Cornette et al. 2016 | 44 women undergoing treatment for breast cancer;  Age: Median of 50.4 years;  BMI: Mean of 25.1 kg.m^-2^  Stage 3-4: 23.8% | Surgery: NR; Radiotherapy: 100%; Chemotherapy: 100%; Hormone therapy: NR; | Combined resistance and aerobic exercise vs. Usual care | **Combined resistance and aerobic exercise**  1 session per week for 27 weeks performing a non-supervised program undertaking 2 sets at in 5 resistance exercises & 2 sessions per week performing Mixed Continuous aerobic exercise for 20 to 40 min per session | 30 out of 44 participants completed the study (68.1%) | Fatigue;  Physical function;  Lower-body muscle strength |
| de Luca et al. 2016 | 20 women previously treatment for breast cancer;  Age: Mean of 45.6 years;  BMI: Mean of 25.2 kg.m^-2^  Stage 3-4: 30% | Surgery: 100%; Radiotherapy: 60%; Chemotherapy: 100%; Hormone therapy: 50%; | Combined resistance and aerobic exercise vs. Control group | **Combined resistance and aerobic exercise**  2 sessions per week for 24 weeks performing a supervised program undertaking 2 to 4 sets at 60% of 1-RM in 5 resistance exercises & 2 sessions per week performing cycle continuous aerobic exercise for 20 to 30 min per session at 80% HR maximum | 20 out of 20 participants completed the study (100%) | Fatigue;  Lower-body muscle strength |
| Dolan et al. 2016 | 36 women previously treatment for breast cancer;  Age: Mean of 57.2 years;  BMI: Mean of 24.9 kg.m^-2^  Stage 3-4: 33.3%; | Surgery: 100%; Radiotherapy: 63.6%; Chemotherapy: 66.7%; Hormone therapy: 63.6%; | Aerobic exercise vs.  Delayed intervention group | **Aerobic exercise**  3 sessions per week for 6 weeks performing a supervised program, at treadmill continuous aerobic exercise for 60% of VO2 peak or interval treadmill aerobic exercise at 95% of VO2 peak | 33 out of 36 participants completed the study (91.6%) | Lower-body muscle strength |
| Galiano-Castillo et al. 2016 & Ariza-Garcia et al. 2019 | 81 women previously treatment for breast cancer;  Age: Mean of 48.3 years;  BMI: NR  Stage 3-4: 13.5% | Surgery: 100%; Radiotherapy: 95.1%; Chemotherapy: 95.1%; Hormone therapy: NR; | Combined resistance and aerobic exercise vs. Delayed intervention group | **Combined resistance and aerobic exercise**  3 sessions per week for 8 weeks performing a supervised program at in resistance exercises & 3 sessions per week performing aerobic exercise | 76 out of 81 participants completed the study (93.8%) | Lean mass;  Physical function;  Lower-body muscle strength |
| Hagstrom et al. 2016 | 39 women previously treatment for breast cancer;  Age: Mean of 51.9 years;  BMI: Mean of 28.7 kg.m^-2^  Stage 3-4: 0%; | Surgery: 100%; Radiotherapy: 100%; Chemotherapy: 97.1%; Hormone therapy: 85.3%; | Resistance exercise vs. Delayed intervention group | **Resistance exercise**  3 sessions per week for 16 weeks performing a supervised program undertaking 3 sets per exercise in 8 resistance exercises at 75% of 1RM | 34 out of 39 participants completed the study (87.1%) | Fatigue;  Lower-body muscle strength |
| Shobeiri et al. 2016 | 60 women previously treatment for breast cancer;  Age: Mean of 43 years;  BMI: Mean of 27.4 kg.m^-2^  Stage 3-4: 0% | Surgery: 100%; Radiotherapy: 45.3%; Chemotherapy: 94.3%; Hormone therapy: 49.1%; | Aerobic exercise vs. Control group | **Aerobic exercise**  2 sessions per week for 10 weeks performing supervised program, with mixed continuous aerobic exercise for 15 to 35 min at 75% of HR maximum | 53 out of 60 participants completed the study (88.3%) | Fatigue |
| Waked et al. 2016 | 52 women previously treatment for breast cancer;  Age: Mean of 49 years;  BMI: NR  Stage 3-4: 0%; | Surgery: 52.2%; Radiotherapy: 10.9%; Chemotherapy: 37%; Hormone therapy: NR; | Combined resistance and aerobic exercise vs. Usual care | **Combined resistance and aerobic exercise**  3 sessions per week for 8 weeks performing a supervised program undertaking 3 sets at 100% of 1-RM in 6 resistance exercises & 3 sessions per week performing Cycle Interval aerobic exercise for 15 min per session at 95% HR maximum | 46 out of 52 participants completed the study (88.4%) | Fatigue |
| Thomas et al. 2017 | 121 women previously treatment for breast cancer;  Age: Mean of 61.2 years;  BMI: Mean of 29.3 kg.m^-2^  Stage 3-4: 8.5% | Surgery: NR; Radiotherapy: 78.5%; Chemotherapy: 48.5%; Hormone therapy: 100%; | Combined resistance and aerobic exercise vs. Usual care | **Combined resistance and aerobic exercise**  2 sessions per week for 52 weeks performing a hybrid program undertaking 3 sets at 12RM in 6 resistance exercises & performing continuous aerobic exercise for 150 min per week at moderate intensity | 84 out of 121 participants completed the study (69.4%) | Lean mass |
| Campbell et al. 2018 | 19 women with breast cancer; Stage 3-4: 10%; Age: Mean of 52.4 years;  BMI: Mean of 26.4 kg.m-2 | Surgery: NR; Radiotherapy: 90%; Chemotherapy: 84.2%; Hormone therapy: NR; | Combined resistance and aerobic exercise vs. Delayed intervention group | **Combined resistance and aerobic exercise**  2 sessions per week for 24 weeks performing a hybrid program at in resistance exercises & sessions per week performing Treadmill Continuous aerobic exercise for 150 min per week per session at moderate intensity | 19 out of 19 participants completed the study (100%) | Fatigue |
| de Paulo et al. 2018 & de Paulo et al. 2019 | 36 women previously treatment for breast cancer;  Age: Mean of 64.9 years;  BMI: Mean of 30.2 kg.m^-2^  Stage 3-4: 17.6%; | Surgery: 100%; Radiotherapy: 76.5%; Chemotherapy: 68%; Hormone therapy: 100%; | Combined resistance and aerobic exercise vs. Stretching control group | **Combined resistance and aerobic exercise**  3 sessions per week for 36 weeks performing a supervised program undertaking 1 to 3 sets at 75% of 1-RM in 8 resistance exercises & 3 sessions per week performing Treadmill Continuous aerobic exercise for 20 to 30 min per session at 80% HR maximum | 29 out of 36 participants completed the study (80.5%) | Fatigue;  Lean mass |
| Dieli-Conwright et al. 2018 [1] & Dieli-Conwright et al. 2018 [2] | 100 women previously treatment for breast cancer;  Age: Mean of 53.5 years;  BMI: Mean of 33.2 kg.m^-2^  Stage 3-4: 22%; | Surgery: 100%; Radiotherapy: 11%; Chemotherapy: 13%; Hormone therapy: 84%; | Combined resistance and aerobic exercise vs. Usual care | **Combined resistance and aerobic exercise**  2 sessions per week for 16 weeks performing a supervised program undertaking 3 sets at 80% of 1-RM in 8 resistance exercises & 3 sessions per week performing Mixed Continuous aerobic exercise for 30 to 50 min per session at 85% HR maximum | 91 out of 100 participants completed the study (91%) | Fatigue;  Lean mass;  Lower-body muscle strength |
| Dieli-Conwright et al. [3] 2018 | 21 women previously treatment for breast cancer;  Age: Mean of 54 years;  BMI: Mean of 33.4 kg.m^-2^  Stage 3-4: 30%; | Surgery: 100%; Radiotherapy: 55%; Chemotherapy: 75%; Hormone therapy: NR; | Combined resistance and aerobic exercise vs. Delayed intervention group | **Combined resistance and aerobic exercise**  2 sessions per week for 16 weeks performing a supervised program undertaking 3 sets at 80% of 1-RM in 8 resistance exercises & 3 sessions per week performing mixed continuous aerobic exercise for 30 to 50 min per session at 80% HR maximum | 20 out of 21 participants completed the study (95.2%) | Lean mass |
| Mijwel et al. 2018 [1] & Mijwel et al. 2018 [2] | 240 women undergoing treatment for breast cancer;  Age: Mean of 53.3 years;  BMI: NR  Stage 3-4: 0%; | Surgery: NR; Radiotherapy: NR; Chemotherapy: 100%; Hormone therapy: NR; | Combined resistance and aerobic exercise vs. Aerobic exercise vs. Usual care | **Combined resistance and aerobic exercise**  2 sessions per week for 16 weeks performing a supervised program undertaking 2 to 3 sets at 70% of 1-RM in 10 resistance exercises  **Aerobic exercise**  2 sessions per week performing a cycle interval aerobic exercise for 3 bouts of 3 min per session at 16-18 out 20 RPE | 182 out of 240 participants completed the study (75.8%) | Fatigue;  Lower-body muscle strength |
| Reis et al. 2018 | 31 women undergoing treatment for breast cancer;  Age: Mean of 46.7 years;  BMI: Mean of 25.6 kg.m^-2^  Stage 3-4: 39.2% | Surgery: NR; Radiotherapy: 21.4%; Chemotherapy: 50%; Hormone therapy: NR; | Combined resistance and aerobic exercise vs. Usual care | **Combined resistance and aerobic exercise**  3 sessions per week for 12 weeks performing a hybrid program undertaking 3 sets at 12RM, 70% of 1RM in resistance exercises & 3 sessions per week performing Cycle Interval aerobic exercise for per session at 90% of target HR | 28 out of 31 participants completed the study (90.3%) | Fatigue |
| Uludag et al. 2018 | 30 women undergoing treatment for breast cancer;  Age: Mean of 53.1 years;  BMI: Mean of 29.7 kg.m^-2^  Stage 3-4: 0%; | Surgery: NR; Radiotherapy: NR; Chemotherapy: NR; Hormone therapy: NR; | Resistance exercise vs. Aerobic exercise | **Resistance exercise**  3 sessions per week for 12 weeks performing a supervised program undertaking 4 resistance exercises at 70% of HR.  **Aerobic exercise**  3 sessions per week for 12 weeks performing a supervised program with aerobic exercise at 70% of HR | 30 out of 30 participants completed the study (100%) | Lean mass |
| Scott et al. 2018 | 65 women undergoing and previously treatment (mixed) for breast cancer;  Age: Mean of 54 years;  BMI: Mean of 28 kg.m^-2^  Stage 3-4: 100% | Surgery: NR; Radiotherapy: NR; Chemotherapy: 56.0%; Hormone therapy: 32%; | Aerobic exercise vs. Stretching control group | **Aerobic exercise**  3 sessions per week for 12 weeks performing supervised program, with treadmill continuous aerobic exercise for 30 to 50 min at 80% of VO2 peak | 47 out of 65 participants completed the study (72.3%) | Fatigue;  Physical function |
| Ammitzboll et al. 2019 | 158 women undergoing treatment for breast cancer;  Age: Mean of 52.5 years;  BMI: NR  Stage 3-4: 20.8%; | Surgery: 100%; Radiotherapy: NR; Chemotherapy: 94.3%; Hormone therapy: 72.8%; | Resistance exercise vs. Usual care | **Resistance exercise**  2 sessions per week for 20 weeks performing a supervised and non-supervised program | 113 out of 158 participants completed the study (71.5%) | Fatigue |
| Baglia et al. 2019 | 121 women previously treatment for breast cancer;  Age: Mean of 61.2 years;  BMI: Mean of 29.3 kg.m^-2^  Stage 3-4: 8.5% | Surgery: NR; Radiotherapy: 78.5%; Chemotherapy: 48.5%; Hormone therapy: 100%; | Combined resistance and aerobic exercise vs. Usual care | **Combined resistance and aerobic exercise**  2 sessions per week for 52 weeks performing a supervised and non-supervised program undertaking 3 sets at in 6 resistance exercises & 3 or 5 sessions per week performing supervised and non-supervised continuous aerobic exercise for 50 or 30 per session at 80% HR maximum | 83 out of 121 participants completed the study (68.6%) | Fatigue |
| Ceseiko et al. 2019 & Ceseiko et al. 2020 | 55 women undergoing treatment for breast cancer;  Age: Mean of 48.6 years;  BMI: NR;  Stage 3-4: 21.8% | Surgery: 100%; Radiotherapy: 89.1%; Chemotherapy: 85.5%; Hormone therapy: 69.1%; | Resistance exercise vs. Chair rise | **Resistance exercise**  2 sessions per week for 12 weeks performing a supervised program undertaking 1 set at in 4 resistance exercises at 90% of 1 RM | 55 out of 55 participants completed the study (100%) | Fatigue;  Physical function;  Lower-body muscle strength |
| Dong et al. 2019 | 60 women previously treatment for breast cancer;  Age: Mean of 49.7 years;  BMI: Mean of 24.6 kg.m^-2^  Stage 3-4: 12%; | Surgery: 100%; Radiotherapy: 100%; Chemotherapy: 100%; Hormone therapy: NR; | Combined resistance and aerobic exercise vs. Control group | **Combined resistance and aerobic exercise**  3 sessions per week for 12 weeks performing a Supervised program undertaking sets at in resistance exercises & 4 sessions per week performing aerobic exercise | 50 out of 60 participants completed the study (83.3%) | Fatigue;  Physical function |
| dos Santos et al. 2019 & Moraes et al. 2021 | 26 women previously treatment for breast cancer;  Age: Mean of 54.6 years;  BMI: Mean of 27.4 kg.m^-2^  Stage 3-4: 8%; | Surgery: 100%; Radiotherapy: 88%; Chemotherapy: 84%; Hormone therapy: 100%; | Resistance exercise vs. Control group | **Resistance exercise**  1 session per week for 8 weeks performing a supervised program undertaking 3 sets at in 5 resistance exercises at 10RM, 75% of 1 RM | 25 out of 26 participants completed the study (96.1%) | Fatigue;  Lean mass;  Lower-body muscle strength |
| Hiraoui et al. 2019 & Hiraoui et al. 2023 | 39 women undergoing treatment for breast cancer;  Age: Mean of 49.4 years;  BMI: Mean of 28 kg.m^-2^  Stage 3-4: 0 %; | Surgery: 100%; Radiotherapy: NR; Chemotherapy: 100%; Hormone therapy: NR; | Combined resistance and aerobic exercise vs. Control group | **Combined resistance and aerobic exercise**  5 sessions per week for 6 weeks performing a supervised and non-supervised program undertaking sets at in 1 resistance exercises & 5 sessions per week performing Mixed Continuous aerobic exercise for 20 to 30 per session at 80% HR maximum | 32 out of 39 participants completed the study (82%) | Physical function;  Lower-body muscle strength |
| McNeil et al. 2019 | 45 women undergoing treatment for breast cancer;  Age: Mean of 58.7 years;  BMI: Mean of 28.5 kg.m^-2^  Stage 3-4: 17.8%; | Surgery: 100%; Radiotherapy: 80%; Chemotherapy: 80%; Hormone therapy: 80%; | Aerobic exercise vs. Control group | **Aerobic exercise**  12 weeks performing non-supervised program, with mixed continuous aerobic exercise for 60 min at 59% of HR reserve & 12 weeks performing non-supervised program, with mixed continuous aerobic exercise for 30 min at 80% of HR reserve | 43 out of 45 participants completed the study (95.5%) | Lean mass |
| Souza Filho et al. 2019 | 75 women previously treatment for breast cancer  Age: Mean of 66.7 years;  BMI: Mean of 29.7 kg.m^-2^  Stage 3-4: 17.8%; | Surgery: 100%; Radiotherapy: 58.7%; Chemotherapy: 53.3%; Hormone therapy: 100%; | Resistance exercise vs. Education control group | **Resistance exercise**  5 sessions per week for 12 weeks performing a non-supervised resistance exercise program | 75 out of 75 participants completed the study (100%) | Physical function |
| Yee et al. 2019 | 14 women undergoing treatment for breast cancer;  Age: Mean of 62.2 years;  BMI: Mean of 28.3 kg.m^-2^  Stage 3-4: 100%; | Surgery: NR; Radiotherapy: NR; Chemotherapy: 36%; Hormone therapy: 50%; | Combined resistance and aerobic exercise vs. Control group | **Combined resistance and aerobic exercise**  2 sessions per week for 8 weeks performing a hybrid program undertaking 2 sets at 6-7 out of 10 RPE in 7 resistance exercises & 2 sessions per week performing Continuous aerobic exercise for 10 to 15 per session at 11-13 out of 20 RPE | 13 out of 14 participants completed the study (83.3%) | Fatigue;  Physical function |
| Hojan et al. 2020 | 68 women undergoing treatment for breast cancer;  Age: Mean of 54.5 years;  BMI: Mean of 24.8 kg.m^-2^  Stage 3-4: 6.4% | Surgery: 100%; Radiotherapy: 100%; Chemotherapy: 100%; Hormone therapy: 72.3%; | Combined resistance and aerobic exercise vs. Usual care | **Combined resistance and aerobic exercise**  5 sessions per week for 9 weeks performing a supervised program undertaking 1 to 3 sets at in 3 resistance exercises & 5 sessions per week performing mixed Continuous aerobic exercise for 45 min per session | 49 out of 68 participants completed the study (96.1%) | Physical function |
| Kim et al. 2020 | 50 women previously treatment for breast cancer;  Age: Mean of 49.2 years;  BMI: NR  Stage 3-4: 16.7% | Surgery: 100%; Radiotherapy: 91.7%; Chemotherapy: 95.8%; Hormone therapy: 47.9%; | Combined resistance and aerobic exercise vs. Control group | **Combined resistance and aerobic exercise**  1 to 2 sessions per week for 12 weeks performing a mixed program undertaking sets at in resistance exercises & 1 to 2 sessions per week performing aerobic exercise | 48 out of 50 participants completed the study (82%) | Fatigue |
| Nouri et al. 2020 | 75 women previously treatment for breast cancer;  Age: Mean of 47 years;  BMI: Mean of 25.1 kg.m^-2^  Stage 3-4: 0%; | Surgery: 100%; Radiotherapy: NR; Chemotherapy: 100%; Hormone therapy: NR; | Resistance exercise vs. Combined resistance and aerobic exercise vs. Control group | **Resistance exercise**  3 sessions per week for 6 weeks performing a supervised program undertaking 2 to 4 sets at in 13 resistance exercises for 12 out of 20 RPE  **Combined resistance and aerobic exercise**  3 sessions per week for 6 weeks performing a supervised program undertaking 2 to 4 sets at in 13 resistance exercises for 12 out of 20 RPE, in addition 3 sessions per week continuous aerobic for 30 min | 57 out of 75 participants completed the study (95.5%) | Physical function |
| Pereira-Rodriguez et al. 2020 | 216 women undergoing treatment for breast cancer;  Age: Mean of 52.9 years;  BMI: Mean of 29.9 kg.m^-2^  Stage 3-4: 0%; | Surgery: NR; Radiotherapy: NR; Chemotherapy: NR; Hormone therapy: NR; | Combined resistance and aerobic exercise vs. Control group | **Combined resistance and aerobic exercise**  3 sessions per week for 36 weeks performing a supervised program undertaking sets at 60% of 1-RM in resistance exercises & 3 sessions per week performing treadmill Continuous aerobic exercise at 80% HR maximum or treadmill interval aerobic for 30 min at 90% of HR maximum. | 216 out of 216 participants completed the study (100%) | Fatigue;  Physical function |
| Santagnello et al. 2020 | 26 women previously treatment for breast cancer;  Age: Mean of 55.2 years;  BMI: Mean of 25.9 kg.m^-2^  Stage 3-4: 0%; | Surgery: 100%; Radiotherapy: 40%; Chemotherapy: 15%; Hormone therapy: 85%; | Resistance exercise vs. Control group | **Resistance exercise**  3 sessions per week for 12 weeks performing a supervised program undertaking 3 sets at in 4 resistance exercises at 10RM, 80% of 1 RM | 20 out of 26 participants completed the study (92.8%) | Fatigue;  Lean mass;  Physical function;  Lower-body muscle strength |
| Scott et al. 2020 | 174 women previously treatment for breast cancer;  Age: Mean of 58 years;  BMI: Mean of 29.5 kg.m^-2^  Stage 3-4: 8%; | Surgery: 100%; Radiotherapy: 71%; Chemotherapy: 89%; Hormone therapy: 71%; | Aerobic exercise vs. Stretching control group | **Aerobic exercise**  3 to 4 sessions per week for 16 weeks performing supervised program, with treadmill continuous aerobic exercise for 40 min at 70% of VO2 peak or treadmill continuous aerobic exercise for 20 to 45 min at >95% of VO2 peak | 156 out of 174 participants completed the study (72%) | Fatigue |
| Aydin et al. 2021 | 48 women previously treatment for breast cancer;  Age: Mean of 45 years;  BMI: NR  Stage 3-4: 0%; | Surgery: 100%; Radiotherapy: NR; Chemotherapy: NR; Hormone therapy: NR; | Combined resistance and aerobic exercise vs. Control group | **Combined resistance and aerobic exercise**  2 sessions per week for 12 weeks performing a hybrid program undertaking in 2 resistance exercises & 3 sessions per week performing Mixed Continuous aerobic exercise for 30 min per session at 60% HR maximum | 48 out of 48 participants completed the study (100%) | Fatigue |
| Brown et al. 2021 [1] & Brown et al. 2021 [2] | 177 women previously treatment for breast cancer;  Age: Mean of 59 years;  BMI: Mean of 34 kg.m^-2^  Stage 3-4: 16.4%; | Surgery: NR; Radiotherapy: 82.5%; Chemotherapy: 78.5%; Hormone therapy: 31.6%; | Combined resistance and aerobic exercise vs. Control group | **Combined resistance and aerobic exercise**  2 sessions per week for 52 weeks performing a hybrid program undertaking 2 to 3 sets at 10RM in 9 resistance exercises & 3 to 6 sessions per week performing mixed continuous aerobic exercise for 30 min per session at Moderate intensity | 137 out of 177 participants completed the study (77,4%) | Fatigue;  Lean mass |
| Gal et al. 2021 | 260 women previously treatment for breast cancer;  Age: Mean of 57.7 years;  BMI: Mean of 26.1 kg.m^-2^  Stage 3-4: 4% | Surgery: NR; Radiotherapy: NR; Chemotherapy: 34.8%; Hormone therapy: 34.8%; | Combined resistance and aerobic exercise vs. Usual care | **Combined resistance and aerobic exercise**  2 sessions per week for 12 weeks performing a supervised resistance exercises program & 2 sessions per week performing supervised aerobic exercise | 176 out of 260 participants completed the study (67.6%) | Fatigue |
| Lee et al. 2021 | 30 women undergoing treatment for breast cancer;  Age: Mean of 46.9 years;  BMI: Mean of 31.6 kg.m^-2^  Stage 3-4: 63%; | Surgery: 23%; Radiotherapy: NR; Chemotherapy: 100%; Hormone therapy: NR; | Aerobic exercise vs. Control group | **Aerobic exercise**  3 sessions per week for 8 weeks performing supervised program, with cycle interval aerobic exercise for 20 min at 90% of peak power output | 30 out of 30 participants completed the study (100%) | Fatigue;  Physical function |
| Moghadam et al. 2021 | 45 women previously treatment for breast cancer;  Age: Mean of 57 years;  BMI: Mean of 28.2 kg.m^-2^  Stage 3-4: 42.5%; | Surgery: 20%; Radiotherapy: 25%; Chemotherapy: 32.5%; Hormone therapy: 85%; | Aerobic exercise vs. Control group | **Aerobic exercise**  3 sessions per week for 12 weeks performing supervised program, with cycle continuous aerobic exercise for 20 to 30 min at 65% of peak power output or cycle interval aerobic exercise for 20 to 30 min at 90% HR maximum | 40 out of 45 participants completed the study (88.8%) | Lean mass;  Lower-body muscle strength |
| Mostafaei et al. 2021 | 60 women undergoing treatment for breast cancer;  Age: Mean of 49.03 years;  BMI: NR  Stage 3-4: 0%; | Surgery: NR; Radiotherapy: NR; Chemotherapy: 100%; Hormone therapy: NR; | Combined resistance and aerobic exercise vs. Usual care | **Combined resistance and aerobic**  exercise 3 sessions per week for 6 weeks performing a non-supervised program at in resistance exercises & 3 sessions per week performing aerobic exercise | 60 out of 60 participants completed the study (100%) | Fatigue |
| Ortiz et al. 2021 | 94 women previously treatment for breast cancer;  Age: Mean of 55.4 years;  BMI: Mean of 31.1 kg.m^-2^  Stage 3-4: 0%; | Surgery: NR; Radiotherapy: NR; Chemotherapy: NR; Hormone therapy: NR; | Combined resistance and aerobic exercise vs. Control group | **Combined resistance and aerobic exercise**  2 sessions per week for 16 weeks performing a non-supervised program undertaking 1 set at in 8 resistance exercises & performing Mixed Continuous aerobic exercise for 150 min per week per session at Moderate intensity | 89 out of 94 participants completed the study (94.6%) | Physical function |
| Samhan et al. 2021 | 63 women previously treatment for breast cancer;  Age: Mean of 49.3 years;  BMI: Mean of 33.3 kg.m^-2^;  Stage 3-4: 15%; | Surgery: 33.3%; Radiotherapy: 8.3%; Chemotherapy: 41.7%; Hormone therapy: NR; | Aerobic exercise vs. Usual care | **Aerobic exercise**  3 sessions per week for 8 weeks a supervised program performing interval treadmill aerobic for 38 min at 90% of HR maximum | 60 out of 63 participants completed the study (95.2%) | Lean mass |
| Bringel et al. 2022 | 31 women previously treatment for breast cancer;  Age: Mean of 46.7 years;  BMI: NR  Stage 3-4: 38.5% | Surgery: NR; Radiotherapy: 19.2%; Chemotherapy: 42.3%; Hormone therapy: NR; | Combined resistance and aerobic exercise vs. Usual care | **Combined resistance and aerobic exercise**  3 sessions per week for 12 weeks performing a supervised program undertaking 3 sets at 12RM, at 70% of 1RM in 5 resistance exercises & 3 sessions per week performing Cycle Continuous aerobic exercise for 30 min per session at 90% HR training | 26 out of 31 participants completed the study (83.8%) | Fatigue |
| Charati et al. 2022 | 76 women undergoing treatment for breast cancer;  Age: Mean of 40.4 years;  BMI: NR  Stage 3-4: 0%; | Surgery: 100%; Radiotherapy: NR; Chemotherapy: 100%; Hormone therapy: NR; | Aerobic exercise vs. Control group | **Aerobic exercise**  2 sessions per week for 5 weeks a supervised program performing aerobic exercise | 70 out of 76 participants completed the study (92.1%) | Physical function |
| Knoerl et al. 2022 | 49 women not treated for breast cancer;  Age: Mean of 52.8 years;  BMI: Mean of 30.2 kg.m^-2^  Stage 3-4: 14.9%; | Surgery: NR; Radiotherapy: NR; Chemotherapy: NR; Hormone therapy: NR; | Combined resistance and aerobic exercise vs. Mind-body | **Combined resistance and aerobic exercise**  2 sessions per week for an average of 28.9 days weeks performing a mixed program in resistance exercises & 2 sessions per week performing Continuous aerobic exercise for 30 to 45 min per session at moderate intensity | 46 out of 49 participants completed the study (93.8%) | Fatigue |
| Lee et al. 2022 | 30 women previously treatment for breast cancer;  Age: Mean of 55 years;  BMI: Mean of 23.9 kg.m^-2^  Stage 3-4: 0%; | Surgery: 100%; Radiotherapy: 100%; Chemotherapy: 100%; Hormone therapy: 70%; | Resistance exercise vs. Control group | **Resistance exercise**  2 to 3 sessions per week for 12 weeks performing a supervised program undertaking 3 to 4 sets at in 8 resistance exercises at 10RM, 80% of 1 RM | 30 out of 30 participants completed the study (100%) | Lean mass;  Physical function |
| Owusu et al. 2022 | 213 women previously treatment for breast cancer;  Age: Mean of 71.8 years;  BMI: Mean of 30.1 kg.m^-2^  Stage 3-4: 0%; | Surgery: 100%; Radiotherapy: 65%; Chemotherapy: 37%; Hormone therapy: 80%; | Combined resistance and aerobic exercise vs. Attention control arm | **Combined resistance and aerobic exercise**  3 sessions per week for 20 weeks performing a hybrid program undertaking 1 to 3 sets at 60% of 1-RM in 6 resistance exercises & 3 sessions per week performing Mixed Continuous aerobic exercise for 30 min per session at 70% HR maximum | 201 out of 213 participants completed the study (94.3%) | Physical function;  Lower-body muscle strength |
| Sturgeon et al. 2022 | 19 women undergoing treatment for breast cancer;  Age: Mean of 49.4 years;  BMI: NR  Stage 3-4: 5%; | Surgery: NR; Radiotherapy: NR; Chemotherapy: 100%; Hormone therapy: NR; | Aerobic exercise vs. Usual care | **Aerobic exercise**  3 sessions per week for 16 to 24 weeks a non-supervised program performing continuous mixed aerobic for 20 to 25 min at 60% of VO2 peak | 17 out of 19 participants completed the study (89.4%) | Fatigue |
| Winters-Stone et al. 2022 | 114 women previously treatment for breast cancer;  Age: Mean of 70.9 years;  BMI: Mean of 29.2 kg.m^-2^  Stage 3-4: 8%; | Surgery: NR; Radiotherapy: 84%; Chemotherapy: 46%; Hormone therapy: 68%; | Resistance exercise vs. Aerobic exercise vs. Flexibility control group | **Resistance exercise**  3 sessions per week for 52 weeks performing a supervised program undertaking 2 to 3 sets at in 10 resistance exercises at 10RM, 75% of 1 RM  **Aerobic exercise**  3 sessions per week for 52 weeks performing a supervised program with continuous aerobic exercise for 20 to 45 min at 65% of estimated heart rate reserve | 114 out of 114 participants completed the study (100%) | Physical function;  Lower-body muscle strength |
| Wonders et al. 2022 | 251 women previously treatment for breast cancer;  Age: Mean of 56.5 years;  BMI: Mean of 29 kg.m^-2^  Stage 3-4: 0%; | Surgery: NR; Radiotherapy: 94%; Chemotherapy: 94%; Hormone therapy: 94%; | Combined resistance and aerobic exercise vs. Usual care | **Combined resistance and aerobic exercise**  1 session per week for 12 weeks performing a supervised program undertaking 3 sets at 10RM, 75% of 1RM in 6 resistance exercises & 1 sessions per week performing Treadmill Continuous aerobic exercise for 30 min per session at 30% of predicted VO2 max | 243 out of 251 participants completed the study (96.8%) | Fatigue |
| Adams-Campbell et al. 2023 | 30 women undergoing treatment for breast cancer;  Age: Mean of 63.9 years;  BMI: Mean of 35.7 kg.m-2;  Stage 3-4: 20%; | Surgery: 100%; Radiotherapy: 100%; Chemotherapy: NR; Hormone therapy: NR; | Aerobic exercise vs. Usual care | **Aerobic exercise**  3 sessions per week for 8 weeks performing a supervised program performing continuous cycle aerobic for 10 min at 60% of VO2 peak | 30 out of 30 participants completed the study (100%) | Fatigue |
| Antunes et al. 2023 | 93 women undergoing treatment for breast cancer;  Age: Mean of 50.3 years;  BMI: Mean of 27.8 kg.m^-2^  Stage 3-4: 34.4%; | Surgery: 31.2%; Radiotherapy: NR; Chemotherapy: 100%; Hormone therapy: NR; | Combined resistance and aerobic exercise vs. Usual care | **Combined resistance and aerobic exercise**  3 to 4 sessions per week performing a supervised program undertaking 2 to 3 sets at 12RM, 70% of 1 RM in 10 resistance exercises & 3 to 4 sessions per week performing mixed continuous aerobic exercise for 20 to 30 min per session at 80% of HR reserve | 85 out of 93 participants completed the study (91.4%) | Fatigue;  Physical function |
| Damato et al. 2023 | 58 women previously treatment for breast cancer;  Age: Mean of 54.3 years;  BMI: Mean of 25.7 kg.m^-2^  Stage 3-4: 0%; | Surgery: 100%; Radiotherapy: 85.7%; Chemotherapy: 91.4%; Hormone therapy: 68.6%; | Resistance exercise vs. Control group | **Resistance exercise**  4 sessions per week for 6 weeks performing a supervised program undertaking 3 sets at in 5 to 6 resistance exercises at 7-8 out of 10 RPE | 35 out of 58 participants completed the study (60.3%) | Lower-body muscle strength |
| Darvishi et al. 2023 | 64 women with breast cancer;  Age: NR;  BMI: NR;  Stage 3-4: NR | Surgery: NR; Radiotherapy: NR; Chemotherapy: 100%; Hormone therapy: NR; | Aerobic exercise vs. Usual care | **Aerobic exercise**  3 sessions per week for 8 weeks performing a supervised program performing continuous cycle aerobic for 35 to 40 min at 75% of HR maximum | 64 out of 64 participants completed the study (100%) | Fatigue |
| Han et al. 2023 | 50 women previously treatment for breast cancer;  Age: Mean of 48.9 years;  BMI: NR  Stage 3-4: 17.4%; | Surgery: 100%; Radiotherapy: 91.3%; Chemotherapy: 95.7%; Hormone therapy: 54.3%; | Combined resistance and aerobic exercise vs. Usual care | **Combined resistance and aerobic exercise**  1 to 2 sessions per week for 12 weeks performing a mixed program undertaking sets at in resistance exercises & 1 to 2 sessions per week performing aerobic exercise for per session | 48 out of 50 participants completed the study (96%) | Fatigue |
| Isanejad et al. 2023 | 30 women previously treatment for breast cancer;  Age: Mean of 45.1 years;  BMI: Mean of 27.5 kg.m^-2^  Stage 3-4: 50%; | Surgery: 26.7%; Radiotherapy: 100%; Chemotherapy: 96.7%; Hormone therapy: 90%; | Aerobic exercise vs. Usual care | **Aerobic exercise**  3 sessions per week for 12 weeks performing a supervised program performing continuous treadmill aerobic for 41 min at 60% of VO2 peak or interval treadmill aerobic for 33 min at 90% of VO2 peak | 30 out of 30 participants completed the study (100%) | Lean mass;  Physical function |
| Mavropalias et al. 2023 | 106 women undergoing treatment for breast cancer;  Age: Mean of 52 years;  BMI: NR  Stage 3-4: NR | Surgery: 96.5%; Radiotherapy: 100%; Chemotherapy: 61.6%; Hormone therapy: 31.4%; | Combined resistance and aerobic exercise vs. Usual care | **Combined resistance and aerobic exercise**  2 to 3 sessions per week for 12 weeks performing a non-supervised program undertaking 1 sets in 8 to 10 resistance exercises & 5 sessions per week performing continuous aerobic exercise for 20 to 30 min per session | 89 out of 106 participants completed the study (83.9%) | Fatigue |
| Soriano-Maldonado et al. 2023 | 60 women previously treatment for breast cancer;  Age: Mean of 52.3 years;  BMI: NR  Stage 3-4: NR | Surgery: 68.3%; Radiotherapy: NR; Chemotherapy: 100%; Hormone therapy: 86.7%; | Resistance exercise vs. 10,000 steps | **Resistance exercise**  2 sessions per week for 6 weeks performing a supervised program undertaking 2 to 3 sets at in 4 resistance exercises at 70% of 1 RM | 58 out of 60 participants completed the study (96.6%) | Fatigue;  Lower-body muscle strength |
| Casanovas-Álvarez et al. 2024 | 64 women previously treatment for breast cancer;  Age: Mean of 52.0 years;  BMI: Mean of 25.2 kg.m^-2^  Stage 3-4: 35.9% | Surgery: 100%; Radiotherapy: NR; Chemotherapy: 100%; Hormone therapy: NR; | Combined resistance and aerobic exercise vs. Usual care | **Combined resistance and aerobic exercise**  2 sessions per week for 12 to 16 weeks performing a supervised program comprising of 10 resistance exercises & 2 sessions per week performing continuous aerobic exercise for 50 min per session | 61 out of 64 participants completed the study (95.3%) | Fatigue |
| Garcia-Roca et al. 2024 & Suárez-Alcázar et al. 2024 | 61 women undergoing treatment for breast cancer;  Age: Mean of 49.6 years;  BMI: Mean of 25.4 kg.m^-2^  Stage 3-4: 4.7% | Surgery: NR; Radiotherapy: NR; Chemotherapy: NR; Hormone therapy: NR; | Combined resistance and aerobic exercise vs. Usual care | **Combined resistance and aerobic exercise**  2 sessions per week for 24 weeks performing a hybrid program undertaking 2 sets of 10-12 repetitions in 11 resistance exercises & 2 sessions per week performing continuous aerobic exercise | 59 out of 61 participants completed the study (96.7%) | Fatigue;  Physical function |
| Vikmoen et al. 2024 | 40 women undergoing treatment for breast cancer;  Age: Mean of 53.2 years;  BMI: Mean of 26.1 kg.m^-2^  Stage 3-4: NR | Surgery: 100%; Radiotherapy: NR; Chemotherapy: 100%; Hormone therapy: NR; | Combined resistance and aerobic exercise vs. Usual care | **Resistance exercise**  2 sessions per week for 16 weeks performing a supervised program undertaking 1 to 3 sets in 5 resistance exercises at 6-10RMs. | 40 out of 40 participants completed the study (100%) | Lean mass |
| Bettariga et al. 2025 | 32 women previously treatment for breast cancer;  Age: Mean of 59.4 years;  BMI: Mean of 28.0 kg.m^-2^  Stage 3-4: 21.4% | Surgery: 100%; Radiotherapy: 60.5%; Chemotherapy: 71.5%; Hormone therapy: 74%; | Resistance exercise vs. Aerobic exercise | **Resistance exercise**  3 sessions per week for 12 weeks performing a supervised program undertaking 3-5 sets of 8-12 repetitions in 8 resistance exercises at 60-80% of 1-RM.  **Aerobic exercise**  3 sessions per week for 12 weeks performing supervised program with interval aerobic exercise for 5-7 bouts of 30 sec at 60–90% of estimated HRmax | 28 out of 32 participants completed the study (87.5%) | Lean mass;  Muscle strength |

1-RM, one-repetition maximum; BMI, body mass index; NR, not reported; RM, maximal repetitions.

**Table S6.** Individual risk of bias assessment for studies examining fatigue in women with breast cancer.

| **Study** | **Randomization**  **process** | **Deviations from**  **intended interventions** | **Missing**  **outcome data** | **Measurement of the outcome** | **Selection of the reported result** | **Overall Bias** |
| --- | --- | --- | --- | --- | --- | --- |
| Segal et al. 2001 | Some concerns | Some concerns | High | Low | Some concerns | High |
| Burnham et al. 2002 | Some concerns | Some concerns | Low | Low | Some concerns | High |
| Courneya et al. 2003 | Some concerns | Some concerns | Low | Low | Some concerns | High |
| Pinto et al. 2003 | High | High | Low | Low | Some concerns | High |
| Campbell et al. 2005 | Some concerns | High | Low | Low | Some concerns | High |
| Drouin et al. 2005 | Some concerns | High | Low | Low | Some concerns | High |
| Mock et al. 2005 | High | Some concerns | Low | Low | Some concerns | High |
| Pinto et al. 2005 | Some concerns | Some concerns | Low | Low | Some concerns | High |
| Battaglini et al. 2006 | Some concerns | Some concerns | Low | Low | Some concerns | High |
| Courneya et al. 2007 | Low | Some concerns | High | Low | Some concerns | Some concerns |
| Daley et al. 2007 | High | Some concerns | Low | Low | Some concerns | High |
| Mutrie et al. 2007 | Some concerns | Some concerns | Low | Low | Low | Some concerns |
| Yuen et al. 2007 | Some concerns | High | High | Low | Some concerns | High |
| Hwang et al. 2008 | Some concerns | Some concerns | Low | Low | Some concerns | High |
| Milne et al. 2008 | Low | Some concerns | Low | Low | Some concerns | Some concerns |
| Cadmus et al. 2009 | Some concerns | Some concerns | Low | Low | Some concerns | High |
| Cadmus et al. 2009 & Irwin et al. 2009 | Some concerns | Some concerns | High | Low | Some concerns | High |
| Moros et al. 2010 | High | High | Low | Low | Some concerns | High |
| Cantarero-Villanueva et al. 2011 | Some concerns | Some concerns | Low | Low | Some concerns | High |
| Winters-Stone et al. 2011 & Winters-Stone et al. 2012 | Some concerns | Some concerns | High | Low | Some concerns | High |
| Duijtis et al. 2012 | Some concerns | Some concerns | High | Low | Some concerns | High |
| Naumann et al. 2012 | High | Some concerns | Low | Low | Some concerns | High |
| Nikander et al. 2012 & Saarto et al. 2012 & Saarto et al. 2012 | Some concerns | Some concerns | Low | Low | Some concerns | Some concerns |
| Cormie et al. 2013 | High | Some concerns | Low | Low | Low | Some concerns |
| Courneya et al. 2013 | Low | Some concerns | Low | Low | Low | Some concerns |
| Ergun et al. 2013 | Some concerns | Some concerns | Low | Low | Some concerns | High |
| Hayes et al. 2013 | Some concerns | Some concerns | Low | Low | Some concerns | High |
| Kulkarni et al. 2013 | High | High | Low | Low | Some concerns | High |
| Rogers et al. 2013 | Some concerns | Some concerns | Low | Low | Low | Some concerns |
| Hornsby et al. 2014 | Some concerns | Some concerns | Low | Low | Some concerns | High |
| Husebo et al. 2014 | Low | Some concerns | High | Low | Some concerns | High |
| Rogers et al. 2014 | Some concerns | Some concerns | Low | Low | Low | Some concerns |
| Steindorf et al. 2014 & Wiskemann et al. 2017 | Some concerns | Some concerns | Low | Low | Low | Some concerns |
| Al-Majid et al. 2015 | High | Some concerns | Low | Low | Some concerns | High |
| Do et al. 2015 | High | High | High | Low | Some concerns | High |
| Do et al. 2015 [2] | High | High | High | Low | Some concerns | High |
| Naraphong et al. 2015 | Some concerns | Some concerns | Low | Low | Some concerns | High |
| Schmidt et al. 2015 | Some concerns | High | High | Low | Some concerns | High |
| Schmidt et al. 2015 [2] | Some concerns | Some concerns | Low | Low | Low | High |
| Travier et al. 2015 | Low | Some concerns | High | Low | Some concerns | High |
| van Waart et al. 2015 | Some concerns | Some concerns | Low | Low | Some concerns | High |
| Cornette et al. 2016 | Some concerns | Some concerns | High | Low | Some concerns | High |
| de Luca et al. 2016 | Some concerns | Some concerns | Low | Low | Some concerns | High |
| Galiano-Castillo et al. 2016 & Ariza-Garcia et al. 2019 | High | Some concerns | High | Low | Low | High |
| Hagstrom et al. 2016 | Some concerns | Some concerns | Low | Low | Low | Some concerns |
| Shobeiri et al. 2016 | Some concerns | Some concerns | Low | Low | Low | Some concerns |
| Waked et al. 2016 | Low | Some concerns | Low | Low | Some concerns | Some concerns |
| Campbell et al. 2018 | Some concerns | Some concerns | Low | Low | Low | Some concerns |
| de Paulo et al. 2018 & de Paulo et al. 2019 | High | Some concerns | Low | Low | Some concerns | High |
| Dieli-Conwright et al. 2018 & Dieli-Conwright et al. 2018 [2] | Low | High | Low | Low | Some concerns | High |
| Mijwel et al. 2018 & Mijwel et al. 2018 | High | Some concerns | Low | Low | Low | High |
| Reis et al. 2018 | High | High | Low | Low | Low | High |
| Scott et al. 2018 | Low | Some concerns | Some concerns | Low | Some concerns | Some concerns |
| Ammitzboll et al. 2019 | Low | Some concerns | Some concerns | Low | Low | Some concerns |
| Baglia et al. 2019 | Some concerns | Some concerns | Low | Low | Some concerns | High |
| Ceseiko et al. 2019 & Ceseiko et al. 2020 | Some concerns | Some concerns | Low | Low | Some concerns | High |
| Dong et al. 2019 & 2020 | Low | High | High | Low | Low | High |
| dos Santos et al. 2019 & Moraes et al. 2021 | High | Some concerns | Low | Low | Some concerns | High |
| Yee et al. 2019 | Some concerns | Some concerns | Low | Low | Low | Some concerns |
| Kim et al. 2020 | Some concerns | Some concerns | Low | Low | Low | Some concerns |
| Pereira-Rodriguez et al. 2020 | Some concerns | Some concerns | Low | Low | Low | Some concerns |
| Santagnello et al. 2020 | High | Some concerns | Low | Low | Low | High |
| Scott et al. 2020 | Some concerns | Some concerns | Low | Low | Some concerns | High |
| Aydin et al. 2021 | Some concerns | Some concerns | High | Low | Some concerns | High |
| Brown et al. 2021 & Brown et al. 2021 [2] | Some concerns | Some concerns | Low | Low | Some concerns | Some concerns |
| Gal et al. 2021 | Some concerns | Some concerns | Low | Low | High | High |
| Lee et al. 2021 | Some concerns | Some concerns | Low | Low | Some concerns | High |
| Mostafaei et al. 2021 | Some concerns | Some concerns | Low | Low | Low | Some concerns |
| Bringel et al. 2022 | High | High | Some concerns | Low | Low | High |
| Knoerl et al. 2022 | Some concerns | High | High | Low | Some concerns | High |
| Sturgeon et al. 2022 | High | Some concerns | Low | Low | Some concerns | High |
| Wonders et al. 2022 | Low | Some concerns | Low | Low | Some concerns | Some concerns |
| Adams-Campbell et al. 2023 | High | Some concerns | Low | Low | Low | High |
| Antunes et al. 2023 | High | Some concerns | Low | Low | Low | High |
| Darvishi et al. 2023 | High | Some concerns | High | Low | Low | High |
| Han et al. 2023 | Some concerns | High | Low | Low | Some concerns | High |
| Soriano-Maldonado et al. 2023 | Some concerns | Some concerns | Low | Low | Low | Some concerns |
| Mavropalias et al. 2023 | Some concerns | High | Low | Low | Low | High |
| Casanovas-Álvarez et al. 2024 | High | Low | Low | Low | Low | High |
| Garcia-Roca et al. 2024 & Suárez-Alcázar et al. 2024 | High | Low | Low | High | High | High |

**Table S7.** Individual risk of bias assessment for studies examining lean mass in women with breast cancer.

| **Study** | **Randomization**  **process** | **Deviations from**  **intended interventions** | **Missing**  **outcome data** | **Measurement of the outcome** | **Selection of the reported result** | **Overall Bias** |
| --- | --- | --- | --- | --- | --- | --- |
| Schmitz et al. 2005 & Ahmed et al. 2006 | Some concerns | Some concerns | High | Low | Some concerns | High |
| Herrero et al. 2006 | Some concerns | Some concerns | High | Low | Some concerns | High |
| Courneya et al. 2007 | Low | Some concerns | High | Low | Some concerns | High |
| Matthews et al. 2007 | Some concerns | Some concerns | Low | Low | Some concerns | High |
| Cadmus et al. 2009 & Irwin et al. 2009 | High | Some concerns | High | Low | Some concerns | High |
| Schmitz et al. 2009 | Some concerns | Some concerns | Low | Low | Some concerns | High |
| Schmitz et al. 2010 | Low | Some concerns | Low | Low | Some concerns | Some concerns |
| DeNysschen et al. 2011 | High | Some concerns | Low | Low | Some concerns | High |
| Winters-Stone et al. 2011 & Winters-Stone et al. 2012 | High | Some concerns | High | Low | Low | High |
| Nikander et al. 2012 & Saarto et al. & 2012 & Saarto et al. 2012 | Some concerns | Some concerns | Low | Low | Some concerns | High |
| Courneya et al. 2013 | Some concerns | Some concerns | Low | Low | Low | Some concerns |
| Fernandez-Lao 2013 | Some concerns | Some concerns | High | Low | Some concerns | High |
| Bruno et al. 2016 | High | Some concerns | Low | Low | Some concerns | High |
| Buchan et al. 2016 | Some concerns | Some concerns | Low | Low | Some concerns | High |
| Galiano-Castillo et al. 2016 & Ariza-Garcia et al. 2019 | Some concerns | Some concerns | High | Low | Low | High |
| Thomas et al. 2017 | High | Some concerns | High | Low | High | High |
| de Paulo et al. 2018 & de Paulo et al. 2019 | High | Some concerns | Low | Low | Some concerns | High |
| Dieli-Conwright et al. 2018 & Dieli-Conwright et al. 2018 [2] | Low | High | Low | Low | Some concerns | High |
| Dieli-Conwright et al. 2018 [3] | Some concerns | High | Low | Low | Some concerns | High |
| Uludag et al. 2018 | High | Some concerns | Low | Low | Some concerns | High |
| dos Santos et al. 2019 & Moraes et al. 2021 | High | Some concerns | Low | Low | Some concerns | High |
| McNeil et al. 2019 | High | Some concerns | Low | Low | Low | High |
| Santagnello et al. 2020 | Some concerns | Some concerns | Low | Low | Low | Some concerns |
| Brown et al. 2021 & Brown et al. 2021 [2] | Some concerns | Some concerns | Low | Low | Low | Some concerns |
| Moghadam et al. 2021 | Some concerns | High | Low | Low | Some concerns | High |
| Samhan et al. 2021 | Some concerns | Some concerns | Low | Low | Some concerns | High |
| Lee et al. 2022 | Some concerns | High | Some concerns | Low | Some concerns | High |
| Isanejad et al. 2023 | High | Some concerns | Low | Low | Some concerns | High |
| Vikmoen et al. 2024 | Low | Some concerns | Low | Low | Low | Some concerns |
| Bettariga et al. 2025 | Some concerns | Some concerns | Low | Low | Low | High |

**Table S8.** Individual risk of bias assessment for studies examining physical function in women with breast cancer.

| **Study** | **Randomization**  **process** | **Deviations from**  **intended interventions** | **Missing**  **outcome data** | **Measurement of the outcome** | **Selection of the reported result** | **Overall Bias** |
| --- | --- | --- | --- | --- | --- | --- |
| Nieman et al. 1995 | High | High | High | Low | Some concerns | High |
| Campbell et al. 2005 | Some concerns | High | Low | Low | Some concerns | High |
| Herrero et al. 2006 | Low | Some concerns | High | Low | Some concerns | High |
| Mutrie et al. 2007 | Some concerns | Some concerns | Low | Low | Low | Some concerns |
| Schwartz et al. 2007 | Some concerns | Some concerns | Low | Low | Some concerns | High |
| Yuen et al. 2007 | Some concerns | High | High | Low | Some concerns | High |
| Portela et al. 2008 | Some concerns | High | Low | Low | Some concerns | High |
| Winters-Stone et al. 2011 & Winters-Stone et al. 2012 | Some concerns | Some concerns | High | Low | Some concerns | High |
| Anderson et al. 2012 | Some concerns | Some concerns | Low | Low | Some concerns | High |
| Milecki et al. 2013 | Some concerns | High | High | Low | Some concerns | High |
| Husebo et al. 2014 | Low | Some concerns | High | Low | Some concerns | High |
| Murtezani et al. 2014 | Some concerns | High | Low | Low | Some concerns | High |
| van Waart et al. 2015 | Some concerns | Some concerns | Low | Low | Some concerns | Some concerns |
| Buchan et al. 2016 | Some concerns | Some concerns | Low | Low | Some concerns | High |
| Cornette et al. 2016 | Some concerns | Some concerns | High | Low | Some concerns | High |
| Galiano-Castillo et al. 2016 & Ariza-Garcia et al. 2019 | Some concerns | Some concerns | High | Low | Low | High |
| Scott et al. 2018 | Low | Some concerns | High | Low | Some concerns | High |
| Ceseiko et al. 2019 & Ceseiko et al. 2020 | Some concerns | Some concerns | Low | Low | Some concerns | High |
| Dong et al. 2019 & 2020 | Low | High | High | Low | Low | High |
| Hiraoui et al. 2019 & Hiraoui et al. 2023 | Some concerns | High | Low | Low | Some concerns | High |
| Souza Filho et al. 2019 | Low | Some concerns | Low | Low | Some concerns | Some concerns |
| Yee et al. 2019 | Some concerns | Some concerns | Low | Low | Low | Some concerns |
| Hojan et al. 2020 | Low | High | High | Low | Some concerns | High |
| Nouri et al. 2020 | Some concerns | High | High | Low | Low | High |
| Pereira-Rodriguez et al. 2020 | Some concerns | Some concerns | Low | Low | Low | Some concerns |
| Santagnello et al. 2020 | Some concerns | Some concerns | Low | Low | High | High |
| Lee et al. 2021 | Some concerns | Some concerns | Low | Low | Some concerns | High |
| Ortiz et al. 2021 | Some concerns | Some concerns | High | Low | Some concerns | High |
| Charati et al. 2022 | Some concerns | Some concerns | Low | Low | Low | Some concerns |
| Lee et al. 2022 | Some concerns | High | High | Low | Some concerns | High |
| Owusu et al. 2022 | Low | Some concerns | Low | Low | Low | Some concerns |
| Winters-Stone et al. 2022 | Some concerns | Some concerns | High | Low | Low | High |
| Antunes et al. 2023 | Some concerns | Some concerns | Low | Low | Low | Some concerns |
| Isanejad et al. 2023 | High | Some concerns | Low | Low | Some concerns | High |
| Garcia-Roca et al. 2024 & Suárez-Alcázar et al. 2024 | High | Low | Low | High | High | High |

**Table S9.** Individual risk of bias assessment for studies examining muscle strength in women with breast cancer.

| **Study** | **Randomization**  **process** | **Deviations from**  **intended interventions** | **Missing**  **outcome data** | **Measurement of the outcome** | **Selection of the reported result** | **Overall Bias** |
| --- | --- | --- | --- | --- | --- | --- |
| Nieman et al. 1995 | Some concerns | High | High | Low | Some concerns | High |
| Schmitz et al. 2005 & Ahmed et al. 2006 | Some concerns | Some concerns | High | Low | Some concerns | High |
| Courneya et al. 2007 | Low | Some concerns | Low | Low | Some concerns | Some concerns |
| Nikander et al. 2007 | Some concerns | Some concerns | Low | Low | Some concerns | High |
| Schwartz et al. 2007 | Some concerns | High | Low | Low | Some concerns | High |
| Milne et al. 2008 | Low | Some concerns | Low | Low | Some concerns | Some concerns |
| Schmitz et al. 2009 | Low | Some concerns | Low | Low | Some concerns | Some concerns |
| Schmitz et al. 2010 | Low | Some concerns | Low | Low | Some concerns | Some concerns |
| Winters-Stone et al. 2011 & Winters-Stone et al. 2012 | Some concerns | Some concerns | High | Low | Some concerns | High |
| Naumann et al. 2012 | High | Some concerns | Low | Low | Some concerns | High |
| Nikander et al. 2012 & Saarto et al. & 2012 & Saarto et al. 2012 | Some concerns | High | High | Low | Some concerns | High |
| Cormie et al. 2013 | High | Some concerns | Low | Low | Low | High |
| Courneya et al. 2013 | Low | Some concerns | Low | Low | Low | Some concerns |
| Steindorf et al. 2014 & Wiskemann et al. 2017 | Some concerns | Some concerns | Low | Low | Low | Some concerns |
| Do et al. 2015 | Some concerns | High | High | Low | Some concerns | High |
| Schmidt et al. 2015 | Some concerns | Some concerns | High | Low | Some concerns | High |
| Travier et al. 2015 | Low | Some concerns | High | Low | Some concerns | High |
| van Waart et al. 2015 | Some concerns | Some concerns | Low | Low | Some concerns | High |
| Cornette et al. 2016 | Some concerns | Some concerns | High | Low | Some concerns | High |
| de Luca et al. 2016 | Some concerns | Some concerns | Low | Low | Some concerns | High |
| Dolan et al. 2016 | High | High | Low | Low | Some concerns | High |
| Hagstrom et al. 2016 | Some concerns | Some concerns | Low | Low | Low | Some concerns |
| Dieli-Conwright et al. 2018 & Dieli-Conwright et al. 2018 [2] | Low | High | Low | Low | Some concerns | High |
| Mijwel et al. 2018 & Mijwel et al. 2018 | High | Some concerns | Low | Low | Low | High |
| Ceseiko et al. 2019 & Ceseiko et al. 2020 | Some concerns | Some concerns | Low | Low | Some concerns | High |
| dos Santos et al. 2019 & Moraes et al. 2021 | Some concerns | Some concerns | Low | Low | Low | Some concerns |
| Hiraoui et al. 2019 & Hiraoui et al. 2023 | Some concerns | High | Low | Low | Low | High |
| Santagnello et al. 2020 | Some concerns | Some concerns | Low | Low | Low | Some concerns |
| Moghadam et al. 2021 | Some concerns | High | Low | Low | Some concerns | High |
| Owusu et al. 2022 | Low | Some concerns | Low | Low | Some concerns | Some concerns |
| Winters-Stone et al. 2022 | Some concerns | Some concerns | High | Low | Low | High |
| Damato et al. 2023 | Some concerns | High | Low | Low | Some concerns | High |
| Soriano-Maldonado et al. 2023 | Some concerns | Some concerns | Low | Low | Low | Some concerns |
| Bettariga et al. 2025 | High | Some concerns | Low | Low | Low | High |

**Table S10.** Side-split analyses from network meta-analysis on fatigue.

| **Comparisons** | **Direct comparison**  **SMD (95% CI)** | **Indirect comparison**  **SMD (95% CI)** | **Difference in estimates**  **SMD (95% CI)** | **p-value** |
| --- | --- | --- | --- | --- |
| **Aerobic exercise vs. Control** | -0.31 (-0.54 to -0.07) | -0.50 (-1.24 to 0.25) | 0.19 (-0.59 to 0.97) | 0.634 |
| **Aerobic exercise vs. Resistance exercise** | 0.19 (-0.51 to 0.89) | 0.20 (-0.23 to 0.62) | -0.01 (-0.82 to 0.81) | 0.989 |
| **Aerobic exercise vs. Combined resistance and aerobic exercise** | 0.12 (-0.52 to 0.77) | 0.15 (-0.16 to 0.46) | 0.03 (-0.74 to 0.68) | 0.939 |
| **Resistance exercise vs. Control** | -0.50 (-0.82 to -0.18) | -0.76 (-2.06 to 0.54) | -0.26 (-1.08 to 1.59) | 0.705 |
| **Combined resistance and aerobic exercise vs. Control** | -0.47 (-0.65 to -0.29) | -0.41 (-1.32 to 0.50) | -0.06 (-0.99 to 0.86) | 0.895 |
| **Combined resistance and aerobic exercise vs. Resistance exercise** | - | 0.05 (-0.31 to 0.41) | - | - |


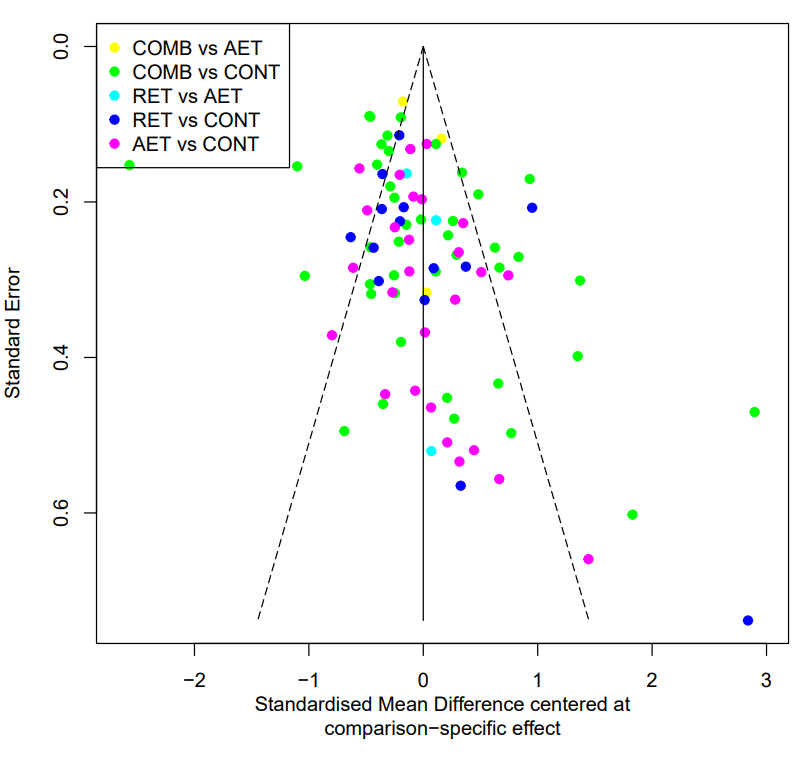


**Figure S1.** Adjusted-funnel plots for network meta-analysis on the effects of exercise programs on fatigue.

**Table S11**. Side-split analyses from network meta-analysis on lean mass.

| **Comparisons** | **Direct comparison**  **SMD (95% CI)** | **Indirect comparison**  **SMD (95% CI)** | **Difference in estimates**  **SMD (95% CI)** | **p-value** |
| --- | --- | --- | --- | --- |
| **Aerobic exercise vs. Control** | 0.34 (-0.10 to 0.77) | 0.52 (0.08 to 0.95) | -0.18 (-0.80 to 0.44) | 0.568 |
| **Aerobic exercise vs. Resistance exercise** | -0.12 (-0.57 to 0.34) | -0.42 (-0.91 to 0.07) | 0.30 (-0.37 to 0.97) | 0.376 |
| **Aerobic exercise vs. Combined resistance and aerobic exercise** | -0.20 (-0.76 to 0.36) | 0.01 (-0.45 to 0.46) | -0.21 (-0.93 to 0.52) | 0.575 |
| **Resistance exercise vs. Control** | 0.75 (0.48 to 1.02) | 0.28 (-0.40 to 0.96) | 0.47 (-0.27 to 1.20) | 0.213 |
| **Combined resistance and aerobic exercise vs. Control** | 0.47 (0.18 to 0.75) | 0.67 (0.01 to 1.34) | -0.21 (-0.93 to 0.52) | 0.575 |
| **Combined resistance and aerobic exercise vs. Resistance exercise** | - | -0.18 (-0.53 to 0.17) | - | - |


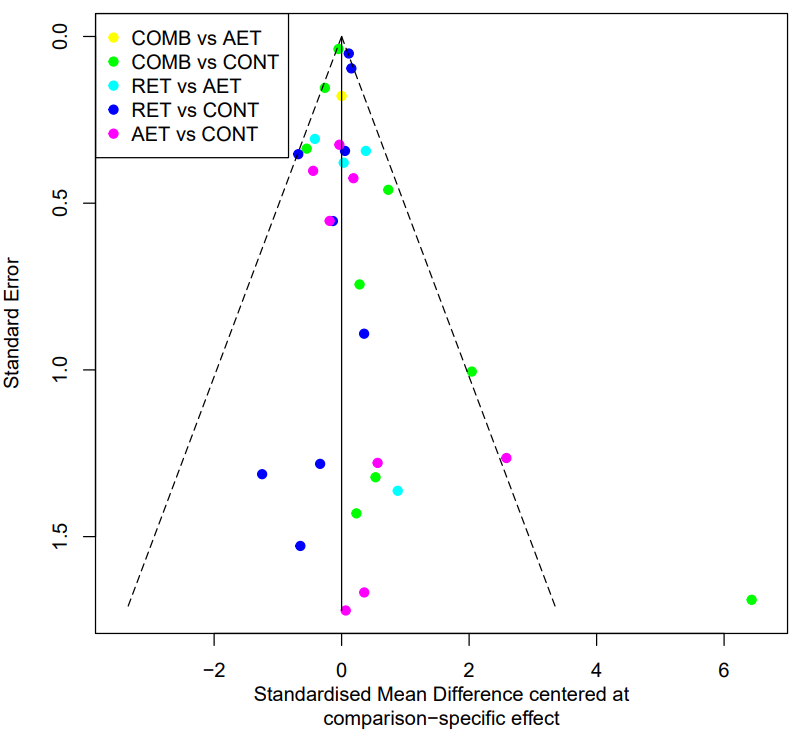


**Figure S2**. Adjusted-funnel plots for network meta-analysis on the effects of exercise programs on lean mass.


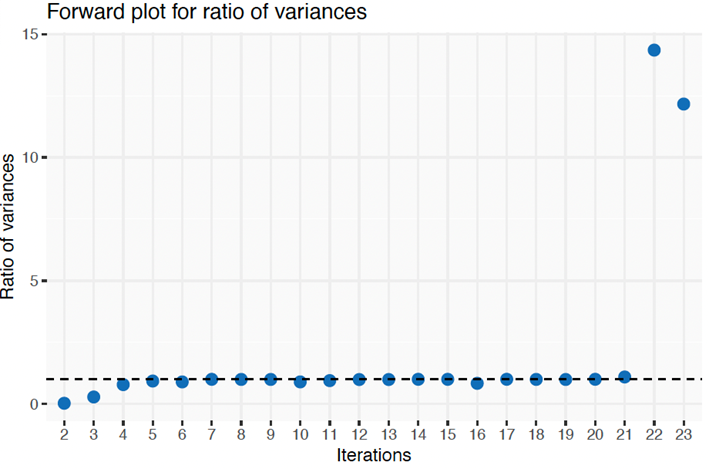


**Figure S3**. Extreme-study effects explored with ratio of variance derived from the forward search algorithm for lean mass. Iteration 22 correspond to the study of Samhan et al. 2021 and iteration 23 correspond to the study of Dieli-Conwright et al. & Dieli-Conwright et al. [2] 2018 & 2018.

**Table S12.** Sensitivity analyses by omitting outliers from network meta-analysis on lean mass.

| **Comparisons** | **SMD (95% CI)** | **P-value** | **I^2^** | **P-score** |
| --- | --- | --- | --- | --- |
|  | **k= 27** | | | |
| AE vs. CTR | 0.39 (0.14 to 0.64) | 0.002 | 12% | RE: 99.9%  COMB: 53.0%  AE: 47.0% |
| RE vs. CTR | 0.78 (0.63 to 0.93) | <0.001 |  |  |
| COMB vs. CTR | 0.42 (0.27 to 0.58) | <0.001 |  |  |
| AE vs. RE | -0.39 (-0.65 to -0.13) | 0.004 |  |  |
| AE vs. COMB | -0.03 (-0.29 to 0.23) | 0.818 |  |  |
| RE vs. COMB | 0.36 (0.15 to 0.57) | <0.001 |  |  |

95% CI, 95% confidence interval; AE, aerobic exercise; COMB, combined resistance and aerobic exercise; k, Number of comparisons; RE, resistance exercise; SMD, standardised mean difference.

**Table S13.** Network meta-analysis results for timed-up and go.

| **Comparisons** | **SMD (95% CI)** | **P-value** | **I^2^** | **P-score** |
| --- | --- | --- | --- | --- |
| **Timed-up and go** | **k= 4** | | | |
| AE vs. CTR | -0.04 (-0.49 to 0.41) | 0.864 | 14% | RE: 84.5%  COMB: 76.6%  AE: 23.0% |
| RE vs. CTR | -0.69 (-1.27 to -0.11) | 0.020 |  |  |
| COMB vs. CTR | -0.61 (-1.28 to 0.05) | 0.070 |  |  |
| AE vs. RE | 0.65 (-0.09 to 1.39) | 0.083 |  |  |
| AE vs. COMB | 0.57 (-0.23 to 1.38) | 0.161 |  |  |
| RE vs. COMB | -0.08 (-0.76 to 0.60) | 0.826 |  |  |

95% CI, 95% confidence interval; AE, aerobic exercise; COMB, combined resistance and aerobic exercise; k, Number of comparisons; RE, resistance exercise; SMD, standardised mean difference.

**Table S14.** Network meta-analysis results for 30-sec chair rise.

| **Comparisons** | **SMD (95% CI)** | **P-value** | **I^2^** | **P-score** |
| --- | --- | --- | --- | --- |
| **30-sec chair rise** | **k= 12** | | | |
| AE vs. CTR | 0.20 (-0.60 to 0.99) | 0.625 | 83% | RE: 97.7%  COMB: 64.8%  AE: 27.0% |
| RE vs. CTR | 1.54 (0.80 to 2.28) | <0.001 |  |  |
| COMB vs. CTR | 0.80 (0.21 to 1.39) | 0.008 |  |  |
| AE vs. RE | -1.34 (-2.42 to -0.26) | 0.015 |  |  |
| AE vs. COMB | -0.60 (-1.59 to 0.38) | 0.232 |  |  |
| RE vs. COMB | 0.74 (-0.21 to 1.69) | 0.125 |  |  |

95% CI, 95% confidence interval; AE, aerobic exercise; COMB, combined resistance and aerobic exercise; k, Number of comparisons; RE, resistance exercise; SMD, standardised mean difference.

**Table S15.** Network meta-analysis results for sit-to-stand test.

| **Comparisons** | **SMD (95% CI)** | **P-value** | **I^2^** | **P-score** |
| --- | --- | --- | --- | --- |
| **Sit-to-stand test** | **k= 5** | | | |
| AE vs. CTR | -0.25 (-2.16 to 1.65) | 0.795 | 89% | RE: 81.1%  COMB: 65.2%  AE: 35.3% |
| RE vs. CTR | -1.23 (-2.54 to 0.09) | 0.067 |  |  |
| COMB vs. CTR | -0.91 (-2.41 to 0.60) | 0.240 |  |  |
| AE vs. RE | 0.98 (-0.93 to 2.88) | 0.315 |  |  |
| AE vs. COMB | 0.65 (-1.78 to 3.08) | 0.599 |  |  |
| RE vs. COMB | -0.32 (-2.32 to 1.68) | 0.752 |  |  |

95% CI, 95% confidence interval; AE, aerobic exercise; COMB, combined resistance and aerobic exercise; k, Number of comparisons; RE, resistance exercise; SMD, standardised mean difference.

**Table S16.** Network meta-analysis results for walking distance tests.

| **Comparisons** | **SMD (95% CI)** | **P-value** | **I^2^** | **P-score** |
| --- | --- | --- | --- | --- |
| **Walking distance tests** | **k= 26** | | | |
| AE vs. CTR | 0.74 (0.20 to 1.29) | 0.008 | 88% | RE: 84.8%  COMB: 68.1%  AE: 46.9% |
| RE vs. CTR | 1.14 (0.46 to 1.82) | 0.001 |  |  |
| COMB vs. CTR | 0.95 (0.53 to 1.37) | <0.001 |  |  |
| AE vs. RE | -0.40 (-1.10 to 0.31) | 0.267 |  |  |
| AE vs. COMB | -0.21 (-0.90 to 0.48) | 0.556 |  |  |
| RE vs. COMB | 0.19 (-0.61 to 0.99) | 0.643 |  |  |

95% CI, 95% confidence interval; AE, aerobic exercise; COMB, combined resistance and aerobic exercise; k, Number of comparisons; RE, resistance exercise; SMD, standardised mean difference.


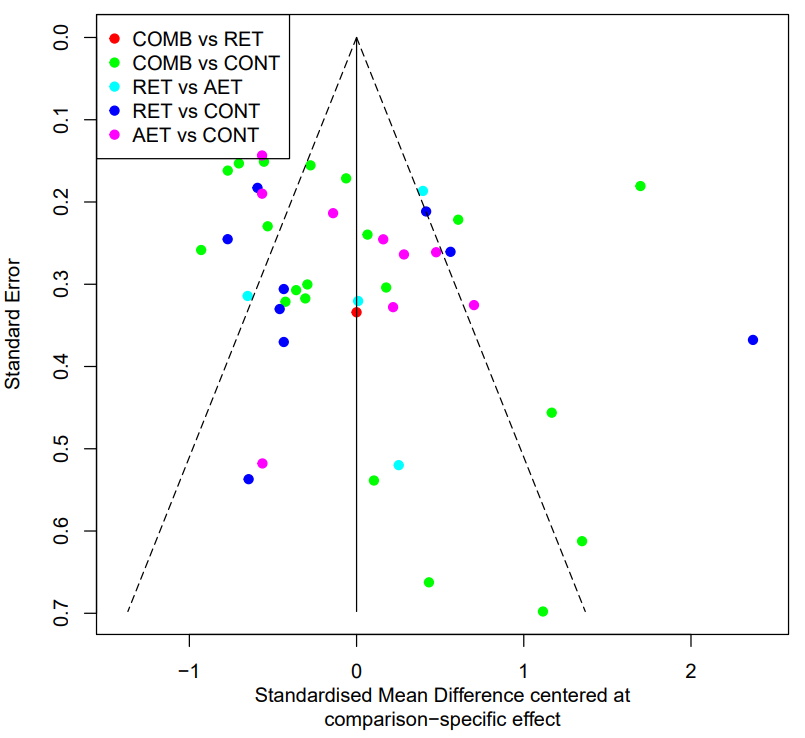


**Figure S4**. Adjusted-funnel plots for network meta-analysis on the effects of exercise programs on physical function outcomes.

**Table S17.** Side-split analyses from network meta-analysis on physical function.

| **Comparisons** | **Direct comparison**  **SMD (95% CI)** | **Indirect comparison**  **SMD (95% CI)** | **Difference in estimates**  **SMD (95% CI)** | **p-value** |
| --- | --- | --- | --- | --- |
| **Aerobic exercise vs. Control** | 0.48 (0.01 to 0.95) | 1.46 (0.15 to 2.77) | -0.98 (-2.37 to 0.41) | 0.167 |
| **Aerobic exercise vs. Resistance exercise** | 0.02 (-0.71 to 0.74) | -0.60 (-1.41 to 0.20) | 0.62 (-0.46 to 1.70) | 0.261 |
| **Aerobic exercise vs. Combined resistance and aerobic exercise** | - | -0.31 (-0.85 to 0.24) | - | - |
| **Resistance exercise vs. Control** | 0.88 (0.40 to 1.36) | 0.72 (-0.45 to 1.89) | 0.16 (-1.11 to 1.42) | 0.810 |
| **Combined resistance and aerobic exercise vs. Control** | 0.89 (0.56 to 1.21) | 1.52 (-1.18 to 4.23) | -0.63 (-3.36 to 2.09) | 0.648 |
| **Combined resistance and aerobic exercise vs. Resistance exercise** | 0.10 (-1.36 to 1.55) | 0.03 (-0.54 to 0.61) | 0.06 (-1.50 to 1.63) | 0.937 |

**Table S18.** Sensitivity analyses by omitting outliers from network meta-analysis on physical function outcomes.

| **Comparisons** | **SMD (95% CI)** | **P-value** | **I^2^** | **P-score** |
| --- | --- | --- | --- | --- |
|  | **k= 33** | | | |
| AE vs. CTR | 0.52 (0.21 to 0.83) | 0.001 | 75% | COMB: 85.8%  RE: 66.1%  AE: 48.1% |
| RE vs. CTR | 0.62 (0.29 to 0.95) | <0.001 |  |  |
| COMB vs. CTR | 0.73 (0.50 to 0.97) | <0.001 |  |  |
| AE vs. RE | -0.10 (-0.49 to 0.29) | 0.608 |  |  |
| AE vs. COMB | -0.22 (-0.60 to 0.17) | 0.277 |  |  |
| RE vs. COMB | -0.11 (-0.51 to 0.28) | 0.577 |  |  |

95% CI, 95% confidence interval; AE, aerobic exercise; COMB, combined resistance and aerobic exercise; k, Number of comparisons; RE, resistance exercise; SMD, standardised mean difference.

**Table S19.** Network meta-analysis results for leg press 1-RM.

| **Comparisons** | **SMD (95% CI)** | **P-value** | **I^2^** | **P-score** |
| --- | --- | --- | --- | --- |
|  | **k= 18** | | | |
| AE vs. CTR | 0.54 (-0.10 to 1.18) | 0.097 | 85% | RE: 97.8%  COMB: 53.2%  AE: 46.3% |
| RE vs. CTR | 1.27 (0.83 to 1.71) | <0.001 |  |  |
| COMB vs. CTR | 0.62 (-0.04 to 1.29) | 0.065 |  |  |
| AE vs. RE | -0.73 (-1.40 to -0.06) | 0.032 |  |  |
| AE vs. COMB | -0.08 (-0.89 to 0.73) | 0.843 |  |  |
| RE vs. COMB | 0.65 (-0.12 to 1.42) | 0.099 |  |  |

95% CI, 95% confidence interval; AE, aerobic exercise; COMB, combined resistance and aerobic exercise; k, Number of comparisons; RE, resistance exercise; SMD, standardised mean difference.

**Table S20.** Network meta-analysis results for leg extension 1-RM.

| **Comparisons** | **SMD (95% CI)** | **P-value** | **I^2^** | **P-score** |
| --- | --- | --- | --- | --- |
|  | **k= 5** | | | |
| AE vs. CTR | 0.29 (-0.86 to 1.44) | 0.621 | 87% | RE: 95.3%  COMB: 54.9%  AE: 34.4% |
| RE vs. CTR | 0.60 (-0.55 to 1.75) | 0.305 |  |  |
| COMB vs. CTR | 1.63 (0.60 to 2.67) | 0.002 |  |  |
| AE vs. RE | -0.31 (-1.46 to 0.84) | 0.595 |  |  |
| AE vs. COMB | -1.34 (-2.89 to 0.20) | 0.089 |  |  |
| RE vs. COMB | -1.03 (-2.58 to 0.51) | 0.192 |  |  |

95% CI, 95% confidence interval; AE, aerobic exercise; COMB, combined resistance and aerobic exercise; k, Number of comparisons; RE, resistance exercise; SMD, standardised mean difference.

**Table S21.** Network meta-analysis results for isometric knee extension.

| **Comparisons** | **SMD (95% CI)** | **P-value** | **I^2^** | **P-score** |
| --- | --- | --- | --- | --- |
|  | **k= 9** | | | |
| AE vs. CTR | 0.26 (-0.41 to 0.94) | 0.444 | 69% | RE: 95.3%  COMB: 54.9%  AE: 34.4% |
| RE vs. CTR | 0.59 (0.03 to 1.14) | 0.039 |  |  |
| COMB vs. CTR | 0.26 (-0.05 to 0.58) | 0.105 |  |  |
| AE vs. RE | -0.32 (-1.20 to 0.55) | 0.471 |  |  |
| AE vs. COMB | 0.00 (-0.67 to 0.67) | 0.996 |  |  |
| RE vs. COMB | 0.32 (-0.32 to 0.96) | 0.321 |  |  |

95% CI, 95% confidence interval; AE, aerobic exercise; COMB, combined resistance and aerobic exercise; k, Number of comparisons; RE, resistance exercise; SMD, standardised mean difference.


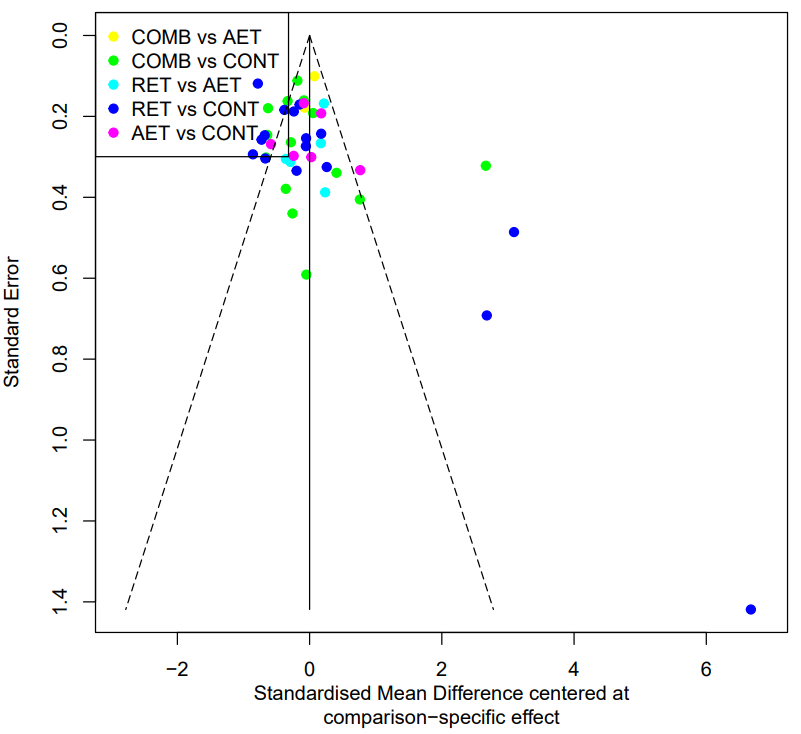


**Figure S5.** Adjusted-funnel plots for network meta-analysis on the effects of exercise programs on muscle strength outcomes.

**Table S22.** Side-split analyses from network meta-analysis on lean mass.

| **Comparisons** | **Direct comparison**  **SMD (95% CI)** | **Indirect comparison**  **SMD (95% CI)** | **Difference in estimates**  **SMD (95% CI)** | **p-value** |
| --- | --- | --- | --- | --- |
| **Aerobic exercise vs. Control** | 0.32 (-0.17 to 0.81) | 0.71 (-0.01 to 1.44) | -0.39 (-1.27 to 0.49) | 0.383 |
| **Aerobic exercise vs. Resistance exercise** | -0.37 (-0.92 to 0.18) | -0.91 (-1.63 to -0.19) | 0.54 (-0.37 to 1.45) | 0.242 |
| **Aerobic exercise vs. Combined resistance and aerobic exercise** | -0.29 (-1.09 to 0.50) | -0.06 (-0.64 to 0.53) | -0.24 (-1.23 to 0.75) | 0.638 |
| **Resistance exercise vs. Control** | 1.00 (0.69 to 1.32) | 1.21 (-0.09 to 2.50) | -0.21 (-1.54 to 1.13) | 0.761 |
| **Combined resistance and aerobic exercise vs. Control** | 0.56 (0.23 to 0.89) | 0.86 (-0.26 to 1.98) | -0.3 (-1.47 to 0.87) | 0.619 |
| **Resistance exercise vs. Combined resistance and aerobic exercise** | - | -0.43 (-0.86 to 0.00) | - | - |

**Table S23.** Sensitivity analyses by omitting outliers from network meta-analysis on muscle strength outcomes.

| **Comparisons** | **SMD (95% CI)** | **P-value** | **I^2^** | **P-score** |
| --- | --- | --- | --- | --- |
|  | **k= 31** | | | |
| AE vs. CTR | 0.31 (0.02 to 0.60) | 0.038 | 71% | RE: 99.7%  COMB: 56.2%  AE: 43.4% |
| RE vs. CTR | 0.78 (0.56 to 1.01) | <0.001 |  |  |
| COMB vs. CTR | 0.39 (0.16 to 0.62) | 0.001 |  |  |
| AE vs. RE | -0.48 (-0.79 to -0.16) | 0.003 |  |  |
| AE vs. COMB | -0.08 (-0.41 to 0.25) | 0.639 |  |  |
| RE vs. COMB | 0.40 (0.08 to 0.71) | 0.014 |  |  |

95% CI, 95% confidence interval; AE, aerobic exercise; COMB, combined resistance and aerobic exercise; k, Number of comparisons; RE, resistance exercise; SMD, standardised mean difference.
